# Comparative effects of exercise-based interventions on jump, linear sprint, and change-of-direction performance in female adolescent team-sport athletes: a systematic review and network meta-analysis

**DOI:** 10.3389/fphys.2026.1867361

**Published:** 2026-07-01

**Authors:** Zhengliang Liu, Xianfeng Yue, Peng Su

**Affiliations:** 1 School of Physical Education, Henan Normal University, Xinxiang, China; 2 School of Physical Education, Guangdong University of Technology, Guangzhou, China

**Keywords:** exercise intervention, female adolescent athletes, network meta-analysis, physical performance, team-sport athletes

## Abstract

**Background:**

As the pace and physical demands of female adolescent team sports continue to rise, jumping, sprinting, and change-of-direction (COD) ability have become key markers of performance. However, current evidence is scattered, and most previous reviews used pairwise meta-analysis, which cannot compare several training methods at once. Therefore, this study used a network meta-analysis (NMA) to compare the effects of different exercise interventions on jumping, straight-line sprinting, and COD performance in female adolescent team-sport athletes.

**Methods:**

PubMed, Web of Science, Cochrane, Embase, SPORTDiscus, CNKI, WanFang, and VIP were searched from inception to March 18, 2026. Eligible studies were RCTs involving healthy female adolescent team-sport athletes. A frequentist random-effects NMA was conducted to calculate mean differences (MDs) with 95% confidence intervals (CIs). Interventions were ranked in combination with SUCRA values. Risk of bias, consistency, heterogeneity, and certainty of evidence were assessed. Sensitivity and subgroup analyses were also performed.

**Results:**

Forty-seven RCTs involving 1,433 athletes were included, covering eight intervention nodes. Overall, CT and PT showed the most consistent benefits for jumping and sprinting performance. For jumping, CT ranked first for CMJ (MD 6.62 cm, 95% CI 3.31 to 9.94; SUCRA 94.2%; moderate-certainty evidence) and SJ (MD 3.18 cm, 95% CI 2.51 to 3.85; SUCRA 82.0%). PT showed the greatest benefit for SLJ (MD 11.93 cm, 95% CI 1.35 to 22.52; SUCRA 64.0%). For sprinting, CT and PT performed best in the 20 m sprint. CT showed an MD of −0.21 s (95% CI −0.34 to −0.07; SUCRA 78.8%), while PT showed an MD of −0.20 s (95% CI −0.32 to −0.08; SUCRA 76.5%; moderate-certainty evidence). PT ranked first for the 30 m sprint (MD −0.67 s, 95% CI −1.03 to −0.30; SUCRA 93.2%). Both PT and CT also produced clear improvements in the 10 m sprint. For COD performance, NMT ranked first in the T-test (MD −1.14 s, 95% CI −1.46 to −0.82; SUCRA 88.4%), PT ranked first in the modified T-test (MD −0.94 s, 95% CI −1.48 to −0.40; SUCRA 95.8%), and CT ranked first in the Illinois test (MD −0.94 s, 95% CI −1.29 to −0.60; SUCRA 91.0%). Sensitivity analyses supported the stability of the main findings. Subgroup analyses suggested that interventions lasting more than 8 weeks and training programs matched to sport-specific movement patterns produced larger gains.

**Conclusions:**

Overall, CT and PT may be more effective for improving jump and linear sprint performance. In contrast, the best approach for improving change-of-direction ability appears to depend on the demands of the specific task. However, the current evidence is generally of low to moderate certainty, highlighting the need for further high-quality studies to confirm these findings.

**Systematic review registration:**

https://www.crd.york.ac.uk/prospero/, identifier CRD420261358990.

## 1 Introduction

In recent years, women’s team sports have developed rapidly worldwide, with clear increases in competitive standard, game pace, and physical intensity across football, basketball, volleyball, handball, and rugby (World Rugby, 2026). These changes have placed greater demands on athletes’ neuromuscular performance. Research shows that team sports are intermittent and high-intensity in nature, involving frequent changes in movement direction. In this context, performance depends less on aerobic capacity alone and more on the ability to produce powerful actions repeatedly and effectively over short periods (Luo et al., 2025).

Evidence from time–motion analysis and GPS tracking shows that jumping, linear sprinting, and change-of-direction ability are key physical qualities in team sports (Martínez-Hernández and Jones, 2024). These abilities directly influence important match actions, including winning possession, creating space, transitioning quickly between attack and defense, and making defensive stops. Although related, each quality reflects a different physical demand. Vertical jumping mainly indicates lower-body explosive power, rate of force development, and neuromuscular coordination (Nimphius et al., 2018). Linear sprinting reflects the ability to generate horizontal force over short distances, especially during the acceleration phase (Luo et al., 2025). Change of direction is more complex, requiring rapid deceleration, control and redirection of body mass, and effective re-acceleration, which places greater demands on eccentric strength and dynamic control (Nimphius et al., 2018). While these three abilities share some common lower-limb strength characteristics, their underlying control mechanisms differ. As a result, each often requires specific and targeted training to be improved effectively (Salaj and Markovic, 2011).

Female adolescent team-sport athletes are a distinct group that warrants specific research attention. Puberty is a crucial period for physical growth and the refinement of movement skills. In girls, peak height velocity usually occurs at around 11–12 years of age, approximately 1.5 to 2 years earlier than in boys (Cossio-Bolaños et al., 2021). During this stage, rapid bone growth may outpace the adaptation of muscles, tendons, and other soft tissues, which can temporarily reduce coordination, proprioception, and movement control—often described as adolescent awkwardness (Retzepis et al., 2025). At the same time, hormonal changes and increases in body fat may reduce relative strength and make bodyweight-dependent actions, such as jumping and sprinting, more difficult (Carmichael et al., 2021; Zhao et al., 2025). Female adolescents also tend to show movement patterns linked to greater injury risk, including a larger Q-angle, lower hamstring-to-quadriceps strength ratios, and a greater tendency toward knee valgus during landing and change-of-direction tasks (Tazabek et al., 2024). Together, these factors increase their susceptibility to non-contact injuries during high-intensity jumping, deceleration, and directional changes, particularly anterior cruciate ligament injuries, which occur more often in females than in age-matched males (Lephart et al., 2005). For this reason, well-designed interventions targeting jumping, sprinting, and change-of-direction performance are important not only for improving athletic ability, but also for reducing injury risk and supporting long-term development in female adolescent team-sport athletes (Luo et al., 2025; Zhao et al., 2025).

Current interventions targeting these key physical abilities mainly include plyometric training, strength training, neuromuscular training, and repeated sprint or change-of-direction training. Plyometric training uses the stretch-shortening cycle to improve rapid force production, which can enhance jumping, short-distance acceleration, and movement efficiency (Lin et al., 2025; Zhao et al., 2025). Strength training supports jumping, sprinting, and change-of-direction performance by increasing lower-limb strength and improving coordination. It may also help correct common weaknesses in female adolescents, particularly limited posterior chain strength and a low hamstring-to-quadriceps ratio (Lephart et al., 2005; Sánchez Pastor et al., 2023; Mănescu, 2025). Neuromuscular training focuses on combining strength, balance, core stability, body awareness, and movement control. Structured programs such as FIFA 11+ have been shown to improve high-risk movement patterns and reduce lower-limb injury rates (Lephart et al., 2005; Chen et al., 2025a; Kambitta Valappil et al., 2026; Wan et al., 2025). Repeated sprint and sport-specific change-of-direction training are more closely matched to the demands of team sports and may be especially effective for improving short sprint performance, complex directional changes, and movement efficiency under fatigue (Nygaard Falch et al., 2019; Chen et al., 2025b; Kong et al., 2025). Overall, these interventions may all benefit female adolescent team-sport athletes, but they differ in their training focus, underlying effects, and practical outcomes (Luo et al., 2025).

Although previous studies have shown that several training approaches can be effective, the current evidence base still has two important limitations. First, female adolescents remain underrepresented in this area of research, and many training recommendations are still drawn from studies of male or adult athletes, which reduces their relevance to this population (Lin et al., 2025; Luo et al., 2025; Zhao et al., 2025). Second, most existing reviews use traditional pairwise meta-analysis, which usually compares only one intervention with a control group. This makes it difficult to evaluate several training approaches within a single framework or to determine which method is likely to be most effective (Christofilos et al., 2022). In practice, however, coaches need more than evidence that an intervention works; they need to know which approach is most appropriate for improving a specific outcome (Fernandes et al., 2024; Curteis et al., 2025). Network meta-analysis helps address this need by combining direct and indirect evidence, allowing multiple interventions to be compared at the same time and their relative effectiveness to be ranked (Su et al., 2020; Christofilos et al., 2022; Curteis et al., 2025). Accordingly, this study used a systematic review and network meta-analysis to compare the effects of different exercise interventions on jumping, linear sprint, and change-of-direction performance in female adolescent team-sport athletes, with the aim of providing more targeted evidence to guide training prescription and evidence-based practice in this population.

## 2 Methods

This study was designed as a systematic review and network meta-analysis and was reported in accordance with PRISMA 2020 and the PRISMA extension for network meta-analysis (PRISMA-NMA). The protocol was registered in PROSPERO (CRD420261358990).

### 2.1 Search strategy

We systematically searched PubMed, Web of Science, Cochrane Library, Embase, SPORTDiscus, and the Chinese databases CNKI, Wanfang, and VIP for randomized controlled trials examining the effects of exercise interventions on the performance of female adolescent team-sport athletes. The search covered all records from database inception to March 18, 2026. To ensure completeness, we also screened the reference lists of relevant systematic reviews for additional eligible studies. The full search strategies for each database are provided in Appendix 1. To support consistent classification and comparison of the interventions included in this review, all training modalities were defined in advance; detailed definitions are presented in **Supplementary Appendix 2** (**Supplementary Table 2.1**).

### 2.2 Eligibility criteria

Inclusion criteria:

1. Participants (P): Healthy adolescent female athletes participating in team sports, such as basketball, soccer, volleyball, handball, or rugby.
2. Interventions (I): Clearly defined exercise training programs, including high-intensity interval training (HIIT), small-sided games (SSG), complex training (CT), plyometric training (PT), strength training (ST), or neuromuscular training (NMT). Detailed definitions are provided in Appendix 2, **Supplementary Table 2.1**.
3. Comparators (C): A control condition (CON) or another exercise intervention that met the inclusion criteria. Detailed definitions are provided in Appendix 2, **Supplementary Table 2.1**.
4. Outcomes (O): At least one extractable outcome related to jumping performance, linear sprint performance, or change-of-direction ability. Jumping performance outcomes included the countermovement jump (CMJ), squat jump (SJ), and standing long jump (SLJ). Linear sprint performance outcomes included 10-m, 20-m, and 30-m sprint tests. Change-of-direction ability outcomes included the T-test, modified T-test (Mod T-test), and Illinois Agility Test (Illinois).
5. Study design (S): Randomized controlled trials, including both two-arm and multi-arm designs.

Exclusion criteria:

1. Participants (P): Participants with health conditions, injuries, or specific diseases, or samples that did not consist of adolescent female team-sport athletes.
2. Interventions (I): Exercise interventions lasting less than four weeks.
3. Comparators (C): Absence of an appropriate active comparison group.
4. Outcomes (O): No extractable data on jumping performance, linear sprint performance, or change-of-direction ability.
5. Study design (S): Conference abstracts, crossover trials, and non-randomized controlled studies.

### 2.3 Study selection

Records identified from the eight databases were imported into EndNote 20.4.1, merged, and deduplicated. Two reviewers then independently screened titles and abstracts using the predefined eligibility criteria. Articles considered potentially relevant underwent full-text review, which was also conducted independently by the same two reviewers. Studies were included in the network meta-analysis only after full agreement on eligibility. Any disagreements were resolved through discussion, with a third senior reviewer making the final decision when needed.

### 2.4 Data extraction

Data were extracted independently by two reviewers using a standardized extraction form, and all entries were checked by a third reviewer. Extracted information included study characteristics (author, publication year, and country or region), participant characteristics (sample size, age, body mass, and height), sport type, intervention duration, weekly training frequency, session length, and details of the intervention and comparator conditions. Outcome data were collected for jump performance, including countermovement jump (CMJ), squat jump (SJ), and standing long jump (SLJ), reported in cm; linear sprint performance, including 10-m, 20-m, and 30-m sprint times, reported in s; and change-of-direction performance, including the T-test, modified T-test (Mod T-test), and Illinois agility test, also reported in s.

### 2.5 Assessment of evidence quality

We assessed the risk of bias in the included trials using version 2 of the Cochrane Risk of Bias tool (RoB 2). This tool examines five domains: the randomization process, deviations from intended interventions, missing outcome data, outcome measurement, and selective reporting. A study was rated as having a low overall risk of bias when all domains were judged to be low risk. It was rated as having some concerns when at least one domain raised concerns, but none was considered high risk. An overall high risk of bias was assigned when at least one domain was rated as high risk.

Because of the nature of exercise interventions, strict double blinding is difficult to achieve in most trials. As a result, deviations from intended interventions may be an important source of uncertainty in this review. To further assess the certainty of evidence in the network meta-analysis, we also applied the CINeMA framework. This approach evaluates six key areas: within-study bias, reporting bias, indirectness, imprecision, heterogeneity, and incoherence.

### 2.6 Statistical analysis

All analyses were conducted in Stata 18.0 (StataCorp, USA) using a frequentist network meta-analysis approach with the mvmeta command. Because all outcomes were continuous, treatment effects were summarized as mean differences (MDs) with 95% confidence intervals (CIs). To ensure valid comparisons, separate networks were built only for outcomes that shared the same definition, reflected the same construct, and could be expressed in a common unit. Accordingly, countermovement jump (CMJ), squat jump (SJ), and standing long jump (SLJ) were analyzed separately. When studies reported the same outcome in different units, values were converted during data extraction before analysis, such as converting meters to centimeters and standardizing time measures to seconds. For sprint and change-of-direction outcomes, values were not multiplied by −1 to reverse the effect direction; therefore, a negative mean difference (MD) indicates a shorter completion time and improved performance. Outcomes were not pooled when clear differences in measurement methods or assessment tools made direct comparison inappropriate. For studies that did not report standard deviations (SDs), missing values were calculated according to Section 6.5.2.3 of Chapter 6 of the *Cochrane Handbook for Systematic Reviews of Interventions*. When standard errors (SEs) were provided, SDs were calculated as SD = SE × √n. When 95% confidence intervals (CIs) were reported, SEs were first calculated as SE = (upper limit − lower limit)/(2 × t0.975, df), and SDs were then obtained using SD = SE × √n. When only P values or t values were available, the corresponding t value was identified using the P value and degrees of freedom, and the SE of the mean difference was calculated as SE = |MD|/t. For independent two-group designs, the pooled SD was then calculated as SDpooled = SE/√(1/n1 + 1/n2) (Higgins et al., 2024).

A random-effects model was used to account for differences across studies in both design and participant or intervention characteristics. Agreement between direct and indirect evidence was assessed at two levels. Overall consistency across the network was examined using the design-by-treatment interaction model, while local consistency was evaluated with the node-splitting method by comparing direct and indirect estimates within closed loops. Network heterogeneity was assessed using the global τ² statistic. Values near 0.04, 0.14, and 0.40 were interpreted as low, moderate, and high heterogeneity, respectively (Rhodes et al., 2015).

The relative effectiveness of each exercise intervention was ranked using SUCRA. To avoid relying on rankings alone, we interpreted the SUCRA results alongside direct comparison evidence, effect sizes, 95% confidence intervals, and CINeMA ratings of evidence certainty. Network plots were used to show the structure of available comparisons, and comparison-adjusted funnel plots were generated to assess possible small-study effects and publication bias. To test the robustness of the findings, sensitivity analyses were performed after excluding studies at high risk of bias. Prespecified subgroup analyses were also conducted based on intervention duration (≤8 weeks vs. >8 weeks) and the biomechanical demands of the sport (jump- and landing-dominant sports vs. sports dominated by multidirectional movement and change of direction).

## 3 Results

### 3.1 Study selection and characteristics

**Figure 1:**
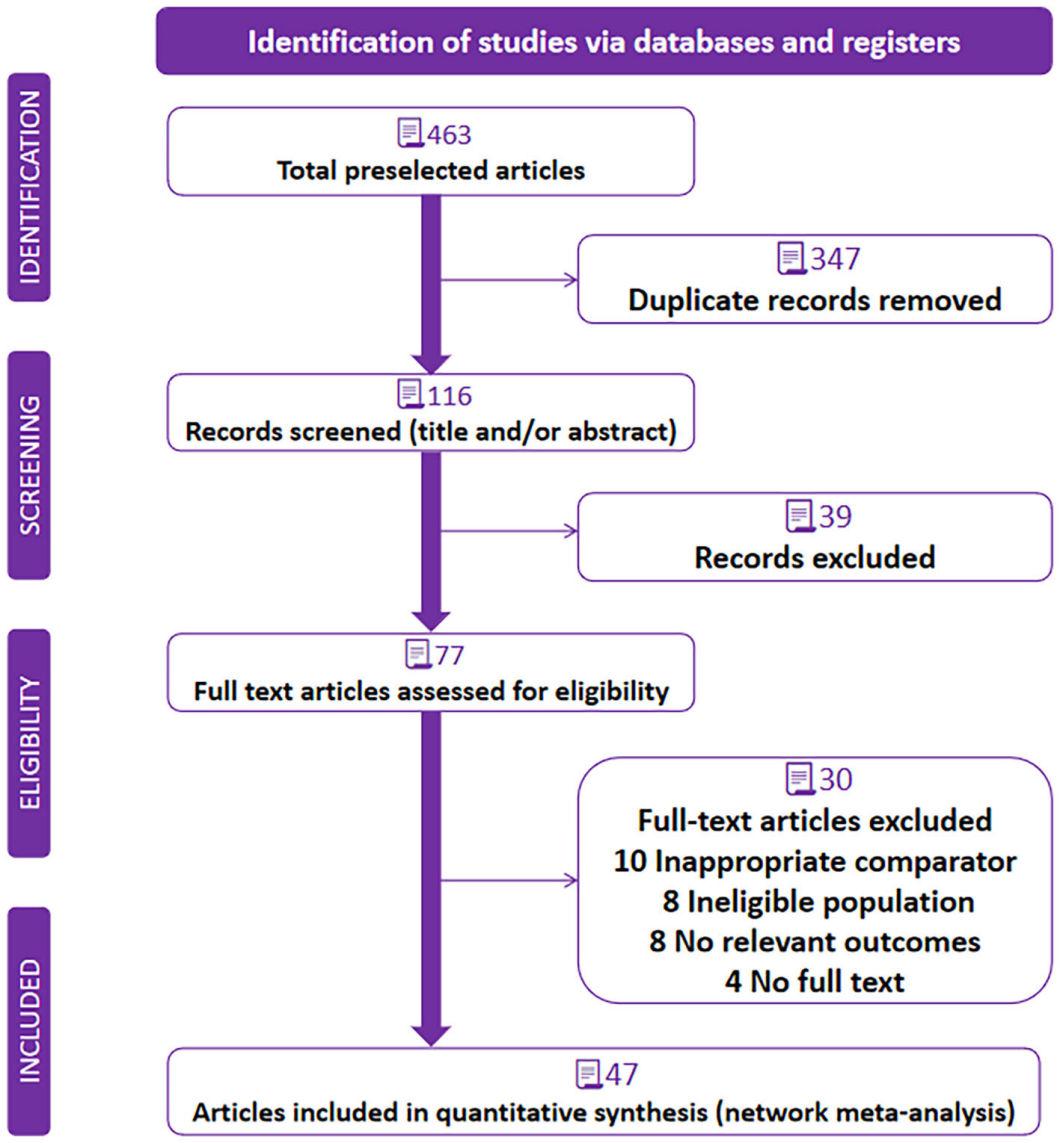
PRISMA flow diagram of study identification, screening, eligibility assessment, and inclusion.

A total of 463 records were identified through the database search. After removing 347 duplicates, 116 articles remained for title and abstract screening, and 39 were excluded at this stage. The full texts of the remaining 77 articles were then reviewed in detail. Ultimately, 47 randomized controlled trials involving 1,433 adolescent female team-sport athletes met the inclusion criteria (**Figure 1**).

The included studies were conducted in 17 countries and regions and covered five main team sports: basketball, soccer, volleyball, handball, and rugby. Intervention periods ranged from 5 to 20 weeks. The athletes had a mean age of 14.97 years (SD = 2.07) and a mean body mass of 56.98 kg (SD = 9.71). Across the 47 studies, the network included eight intervention nodes: HIIT, SSG, NMT, PT, CT, ST, HIIT+SSG, and CON (**Table 1**).

**Table 1:** Baseline characteristics of the included studies.

Study	Study details						Subject details			Outcome
	Country	Sample size	Sport	Duration (weeks)	Frequency (times per week)	Session duration (min)	Age (years)	Weight (kg)	Height (cm)	
(Jurišić et al., 2021)	Serbia	HIIT:12 SSG:12	Handball	8	2	90	16.20 ± 1.28 16.06 ± 0.80	62.46 ± 7.86 61.27 ± 3.68	168.00 ± 6.80 164.00 ± 4.70	1,2,4,5
(Zhou et al., 2025)	China	HIIT+SSG:27 CON:27	Soccer	6	2	NR	12.18 ± 1.25 12.30 ± 1.48	56.96 ± 8.28 52.69 ± 8.23	157.83 ± 7.50 156.44 ± 5.12	6
(Aschendorf et al., 2019)	Germany	HIIT:11 CON:14	Basketball	5	2	25	15.10 ± 1.10	61.00 ± 6.00	170.00 ± 5.17	1,2,3
(Wen et al., 2024)	China	SSG:16 HIIT:16 CON:16	Soccer	8	2	NR	17.20 ± 1.20 17.10 ± 0.80 17.00 ± 1.10	54.40 ± 6.50 55.50 ± 6.90 56.00 ± 6.00	163.00 ± 6.00 163.00 ± 4.00 162.00 ± 4.00	1
(Nayıroğlu et al., 2022)	Türkiye	SSG:12 HIIT:12	Soccer	8	3	15-20	18.80 ± 2.70 18.50± 2.10	54.00 ± 7.30 54.80 ± 8.70	163.60 ± 5.20 161.30 ± 4.70	1,4
(Xu, 2024)	China	NMT:17 CON:17	Volleyball	8	3-4	60	15.52 ± 0.34 15.53 ± 0.37	65.38 ± 2.04 63.60 ± 1.21	177.01 ± 0.99 176.79 ± 1.06	6
(Pang, 2024)	China	PT:9 ST:9	Basketball	12	3	45	13.89 ± 0.60 13.78 ± 0.66	49.67 ± 3.80 49.22 ± 5.24	165.78 ± 4.08 166.00 ± 3.50	5,8
(Wang and Zuo, 2023)	China	PT:20 ST:10	Rugby	8	3	NR	17.05 ± 1.39 16.80 ± 1.47	57.15 ± 4.87 58.65 ± 5.61	166.60 ± 3.88 166.90 ± 4.70	3,6
(Ma, 2021)	China	CT:10 ST:10	Basketball	12	3	70	16.30 ± 0.67 16.20 ± 0.78	67.8 ± 10.5 73.3 ± 13.4	175.00 ± 6.96 174.00 ± 8.23	1,3,8
(Deng, 2020)	China	PT:10 CON:10	Volleyball	12	3	30	NR	55.10 ± 6.38 56.90 ± 6.41	173.70 ± 7.46 173.20 ± 6.71	3
(Chaabene et al., 2021b)	Tunisia	NMT:11 CT:12	Handball	8	2	45	16.80 ± 0.30 16.90 ± 0.20	63.30 ± 5.10 63.70 ± 5.00	164.00 ± 6.90 164.80 ± 6.30	1,3,4,5,8
(Gaamouri et al., 2023a)	Tunisia	ST:17 CON:17	Handball	10	2	NR	15.70 ± 0.20 15.80 ± 0.20	63.40 ± 3.80 63.00 ± 3.80	169.00 ± 4.20 167.00 ± 3.50	1,2,3,7
(Hammami et al., 2019a)	Tunisia	CT:14 CON:14	Handball	10	2	40	16.60 ± 0.30 16.60 ± 0.30	60.80 ± 4.70 60.40 ± 4.30	163.00 ± 4.00 164.00 ± 4.00	1,2,4,5,6,7,9
(Hammami et al., 2022b)	Tunisia	ST:13 CON:13	Handball	10	2	35	15.70 ± 0.20 15.80 ± 0.20	64.00 ± 3.00 64.00 ± 4.00	170.00 ± 4.00 167.00 ± 4.00	1,2,4,5,6,8,9
(Hammami and Zmijewski, 2024)	Tunisia	CT:12 ST:12 CON:12	Handball	10	2	45	16.20 ± 0.30 16.20 ± 0.40 16.30 ± 0.30	64.30 ± 4.00 63.80 ± 3.30 63.50 ± 4.40	167.10 ± 3.70 165.50 ± 3.20 167.60 ± 3.60	1,2,3,4,5,9
(Gaamouri et al., 2024)	Tunisia	CT:16 CON:14	Handball	10	2	45	15.80 ± 0.30 15.80 ± 0.20	64.10 ± 3.50 63.40 ± 4.10	167.00 ± 3.40 167.00 ± 3.20	2,3,7
(Meszler and Váczi, 2019)	Hungary	PT:9 CON:9	Basketball	7	2	20	15.80 ± 1.20 15.70 ± 1.30	63.50 ± 8.60 66.10 ± 8.90	176.40 ± 8.60 177.50 ± 7.40	1,8
(Pardos-Mainer et al., 2020)	Spain	CT:19 CON:18	Soccer	8	2	35	16.20 ± 0.90 15.60 ± 0.90	55.90 ± 5.50 54.10 ± 8.80	159.80 ± 5.40 159.70 ± 4.90	1,3,4,5,6
(Váczi et al., 2022)	Hungary	ST:13 CON:10	Handball	20	1-2	NR	11.30 ± 0.50 10.90 ± 0.50	41.50 ± 7.00 41.10 ± 5.60	150.00 ± 6.60 146.40 ± 3.40	1
(Ortega et al., 2020)	Colombia	ST:28 CON:18	Soccer	12	3	NR	13.60 ± 1.20 13.60 ± 1.20	47.50 ± 5.60 42.50 ± 5.90	158.30 ± 6.70 153.00 ± 5.80	1,2,6
(Hammami et al., 2022a)	Tunisia	CT:15 CON:15	Handball	10	2	45	15.70 ± 0.30 15.70 ± 0.20	64.10 ± 3.60 63.30 ± 3.90	166.50 ± 3.50 167.00 ± 4.10	1,2,4,5,6,9
(Genc et al., 2019)	Türkiye	ST:10 CON:10	Handball	8	3	60	17.80 ± 1.40 17.60 ± 1.89	60.03 ± 7.90 63.30 ± 6.30	153.20 ± 5.80 166.60 ± 5.80	1,3,4,6
(Fort et al., 2012)	Spain	ST:12 CON:11	Basketball	15	10	7-10.5	15.80 ± 1.00 15.80 ± 1.30	71.70 ± 7.50 70.30 ± 9.80	182.00 ± 0.60 182.00 ± 0.60	1
(Mathisen and Danielsen, 2014)	Norway	HIIT:13 CON:13	Soccer	8	1	60	13.60 ± 0.20 13.70 ± 0.30	52.50 ± 9.30 53.80 ± 7.80	157.60 ± 3.80 159.60 ± 5.70	4,5
(Bouteraa et al., 2020)	Tunisia	CT:16 CON:10	Basketball	8	2	45	16.40 ± 0.50 16.50 ± 0.50	56.60 ± 8.30 55.60 ± 7.00	168.00 ± 5.00 168.00 ± 8.00	1,2,4,5,9
(Noutsos et al., 2024)	Greece	PT:27 CON:12	Handball	10	2	12-24	12.90 ± 0.55 12.80 ± 0.50	54.61 ± 7.79 64.90 ± 6.00	159.88 ± 4.77 162.80 ± 2.60	1,3,4,5,7
(Gaamouri et al., 2023b)	Tunisia	PT:14 CON:14	Handball	10	2	NR	15.70 ± 0.20 15.80 ± 0.20	63.80 ± 3.30 63.30 ± 4.10	165.00 ± 3.00 167.00 ± 3.00	1,2,3,7
(Falch et al., 2022)	Norway	ST:11 PT:10	Handball	8	1-2	NR	17.50 ± 2.30 17.10 ± 2.40	65.80 ± 5.90 67.10 ± 9.30	169.20 ± 5.40 173.10 ± 6.60	4,5,6
(Idrizovic et al., 2018)	Kosovo	PT:13 SSG:17 CON:17	Volleyball	12	2	40-60	16.60 ± 0.60 16.60 ± 0.60 16.60 ± 0.60	62.70 ± 7.70 57.00 ± 9.50 56.60 ± 9.50	174.10 ± 3.60 173.90 ± 5.10 174.40 ± 5.30	1,5
(Pereira et al., 2015)	Portugal	PT:10 CON:10	Volleyball	8	2	20	14.00 ± 0.00 13.80 ± 0.40	52.00 ± 7.00 53.50 ± 4.70	160.00 ± 10.00 160.00 ± 10.00	1
(Martel et al., 2005)	United States	PT:10 CON:9	Volleyball	6	2	45	15.00 ± 1.00 14.00 ± 1.00	64.00 ± 13.00 57.00 ± 8.00	167.00 ± 9.00 164.00 ± 8.00	1
(Chaabene et al., 2021a)	Tunisia	PT:12 CON:11	Handball	8	2	25-30	15.90 ± 0.20 15.90 ± 0.30	62.80 ± 7.10 63.70 ± 5.80	164.00 ± 0.10 165.00 ± 0.10	1,4,5,8
(Hammami et al., 2020)	Tunisia	PT:17 CON:17	Handball	10	2	35	15.80 ± 0.20 15.80 ± 0.20	64.20 ± 3.30 63.00 ± 3.80	166.00 ± 3.00 167.00 ± 4.00	1,2,4,5,6,9
(Ozbar et al., 2014)	Türkiye	PT:9 CON:9	Soccer	8	1	60	18.30 ± 2.60 18.00 ± 2.00	58.80 ± 7.80 54.40 ± 6.10	163.10 ± 5.30 159.40 ± 5.10	1,3,5
(Rubley et al., 2011)	United States	PT:10 CON:10	Soccer	14	1	NR	13.40 ± 0.50 13.40 ± 0.50	50.84 ± 5.10 50.84 ± 5.10	162.50 ± 5.67 162.50 ± 5.67	1
(Idrıss et al., 2022)	Algeria	PT:11 CON:11	Soccer	10	3	15-18	15.16 ± 0.93 15.08 ± 0.19	50.33 ± 6.11 49.66 ± 6.02	153.00 ± 6.47 151.50 ± 5.57	1,2
(Haghighi et al., 2024)	Iran	PT:8 HIIT:8 CON:8	Basketball	6	2	30-60	14.60 ± 1.50 15.10 ± 1.60 15.10 ± 1.80	61.70 ± 10.30 53.50 ± 3.00 56.70 ± 13.60	168.30 ± 8.70 167.00 ± 5.50 165.80 ± 9.70	5
(Rojano Ortega et al., 2022)	Spain	PT:14 CON:14	Volleyball	7	2	45	16.07 ± 1.07 15.71 ± 0.73	67.82 ± 5.53 61.30 ± 7.23	166.79 ± 1.89 165.57 ± 4.86	1
(Attene et al., 2015)	Italy	PT:18 CON:18	Basketball	6	2	20	14.83 ± 0.92 15.20 ± 0.92	51.89 ± 9.69 57.50 ± 5.70	163.00 ± 8.62 165.00 ± 5.70	1,2
(Paes et al., 2022)	Italy	PT:11 CON:10	Basketball	6	2	30-60	14.45 ± 0.69 15.30 ± 1.16	53.72 ± 9.01 59.98 ± 16.74	160.00 ± 7.00 163.00 ± 8.00	5,9
(Hammami et al., 2019b)	Tunisia	PT:21 CON:20	Handball	9	2	17	13.50 ± 0.30 13.30 ± 0.30	42.60 ± 4.60 42.30 ± 4.50	142.00 ± 4.00 143.00 ± 4.00	1,2,4,5,6,7,9
(Trajković and Bogataj, 2020)	Serbia	NMT:32 CON:34	Volleyball	8	2	30	11.12 ± 0.68 10.96 ± 0.75	47.83 ± 8.97 48.59 ± 13.46	158.28 ± 8.07 157.37 ± 10.21	1,4,7
(Hou, 2022)	China	NMT:16 PT:16	Volleyball	12	3	90	14-16 14-16	61.50 ± 6.22 59.25 ± 4.71	173.25 ± 5.63 171.31 ± 3.82	3
(Shui and Fu, 2018)	China	NMT:9 ST:9	Soccer	6	3	90	17.43 ± 0.96 17.45 ± 0.83	48.72 ± 7.97 49.88 ± 7.11	162.52 ± 4.43 161.89 ± 3.67	1,6
(Lindblom et al., 2012)	Sweden	NMT:28 CON:24	Soccer	11	2	15	14.20 ± 0.70 14.20 ± 1.10	53.90 ± 8.60 51.60 ± 7.40	165.00 ± 6.50 164.20 ± 6.10	1,4,5,9
(Haklı et al., 2026)	Türkiye	NMT:20 CON:20	Basketball	8	2	25	15.23 ± 1.11 14.77 ± 0.65	60.60 ± 7.57 58.73 ± 9.41	173.00 ± 6.00 169.00 ± 5.00	1
(Gavala et al., 2023)	Greece	NMT:34 CON:27	Volleyball	12	2	40-50	12.08 ± 0.11 11.85 ± 0.95	48.56 ± 0.90 49.11 ± 0.80	158.00 ± 7.00 156.00 ± 7.00	1,4,8

HIIT denotes high-intensity interval training; SSG, small-sided games; CT, complex training; PT, plyometric training; ST, strength training; NMT, neuromuscular training; NR, Not Reported. The outcome measures were coded as follows: CMJ = 1, SJ = 2, SLJ = 3, Sprint 10 = 4, Sprint 20 = 5, Sprint 30 = 6, Modified T-test = 7, T-test = 8, and Illinois = 9.

### 3.2 Risk of bias, confidence in the evidence, and consistency

**Figure 2:**
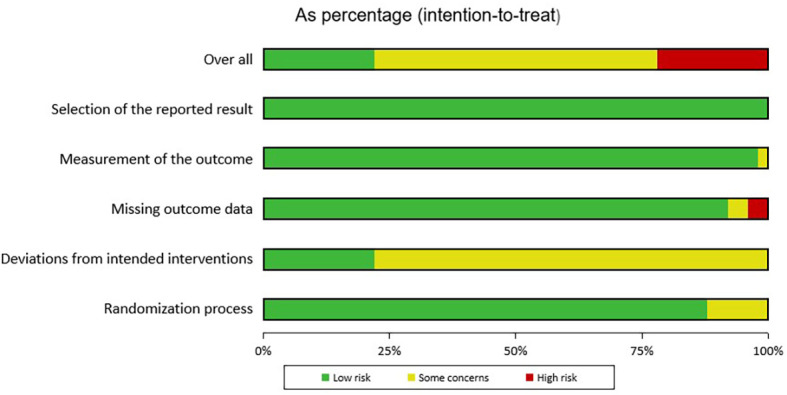
Risk-of-bias summary of the included randomized controlled trials.

The risk-of-bias assessments are provided in Appendix 3. Because exercise interventions are difficult to blind, most studies could not fully blind participants, trainers, or assessors, which is a common limitation in this field. Among the 47 trials, 41 (87.2%) were rated as low risk for the randomization process, 11 (23.4%) for deviations from intended interventions, 43 (91.4%) for missing outcome data, and 46 (97.8%) for outcome measurement. All studies were judged to be at low risk of selective reporting. Overall, 11 studies (23.4%) were classified as high risk of bias, 25 (53.2%) raised some concerns, and 11 (23.4%) were rated as low risk (**Figure 2**).

The consistency analyses showed no significant global inconsistency for any outcome, although the modified T-test was close to the threshold for significance (*p* = 0.0569). Node-splitting analyses also showed good agreement between direct and indirect evidence in most comparisons (**Supplementary Appendix 4**, **Supplementary Tables S4.1–S4.10**). The global τ² results indicated that heterogeneity differed across outcomes. CMJ (τ² = 0.7401) and SLJ (τ² = 1.1073) showed high heterogeneity, while Sprint 30 m (τ² = 0.1019) and the T-test (τ² = 0.0862) showed moderate heterogeneity. Low heterogeneity was observed for the remaining outcomes: SJ (τ² = 0.0113), Sprint 10 m (τ² = 0.0035), Sprint 20 m (τ² = 0.0239), the modified T-test (τ² = 0.0338), and the Illinois Agility Test (τ² < 0.04). (**Supplementary Appendix 4**, **Supplementary Table 4.1**).

The CINeMA assessment further showed that most comparisons for CMJ and 20-m sprint were supported by low to very low certainty evidence. In contrast, the certainty of evidence for the T-test ranged from low to high, and some direct comparisons reached moderate or high certainty. Overall, the evidence across the network was mainly of low to moderate certainty (**Supplementary Appendix 8**).

### 3.3 Jump performance

**Figure 3:**
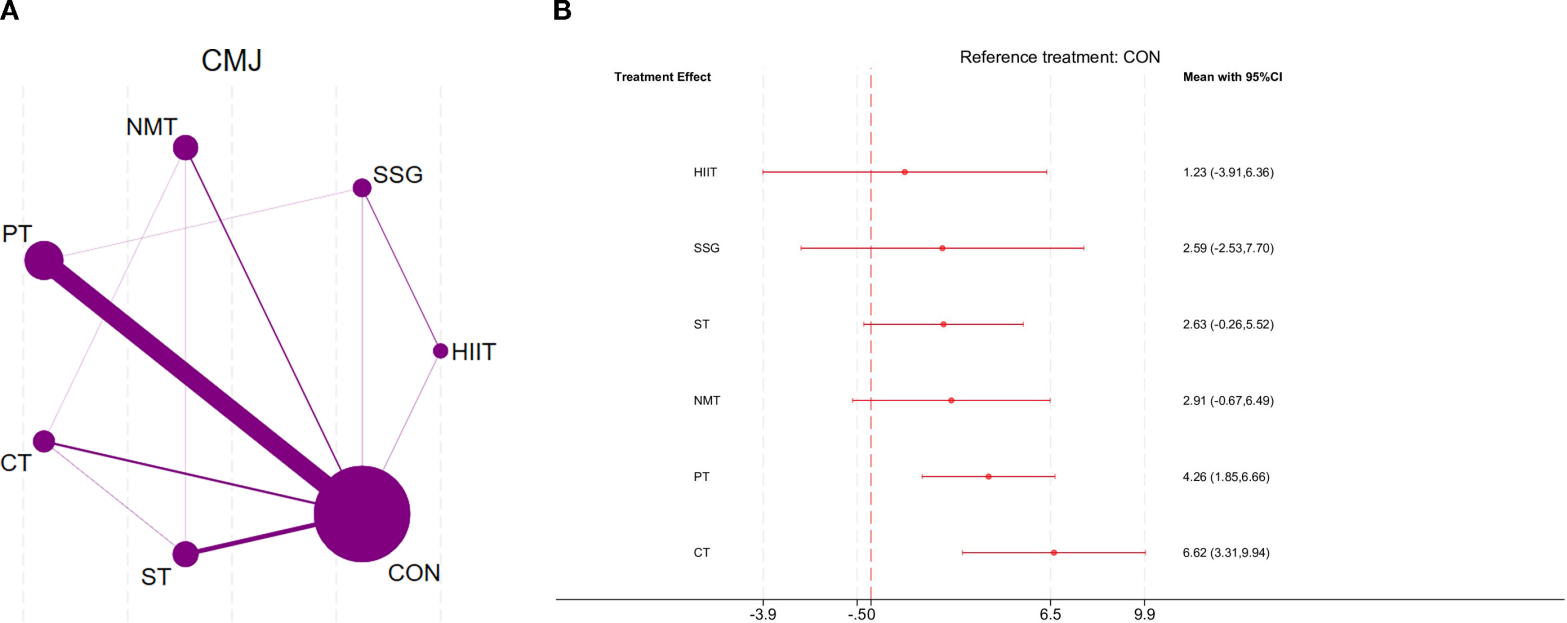
Network and forest plots for the CMJ outcome. **(A)** The network plot shows the direct comparisons among training interventions for the CMJ outcome, including CON, PT, CT, ST, NMT, SSG, and HIIT. Node size is proportional to the total number of participants in each intervention, and line thickness reflects the number of participants included in each direct comparison. **(B)** The forest plot presents the effect estimates and 95% confidence intervals for each intervention versus CON in the network meta-analysis. Effect estimates greater than 0 favor the intervention listed on the left. The interventions are presented in sequence to make the results easier to interpret.

Across the three jump outcomes, CT and PT consistently produced the largest improvements. For countermovement jump (CMJ; 36 RCTs, n = 1102), the network included seven intervention nodes, with CON as the central comparator. The strongest direct evidence came from comparisons between PT and CON. All active interventions tended to improve CMJ performance compared with CON, but only PT and CT reached statistical significance (**Figure 3**). CT showed the greatest effect (MD = 6.62 cm, 95% CI: 3.31 to 9.94; SUCRA = 94.2%), followed by PT (MD = 4.26 cm, 95% CI: 1.85 to 6.66; SUCRA = 70.5%). Both findings were supported by moderate-certainty evidence (**Figure 3**; **Supplementary Appendix 6**, **Supplementary Figure 6.1**). Direct comparisons further showed that CT was superior to ST (MD = 4.00 cm, 95% CI: 0.05 to 7.94) (Appendix 7, **Supplementary Table 7.1**). Most other CMJ comparisons were rated as low or very low certainty, mainly because of imprecision and variation between studies (**Supplementary Appendix 8**, **Supplementary Table 8.2**). A similar pattern was observed for squat jump (SJ; 15 RCTs, n = 465). The network included six intervention nodes, again with CON as the main comparator. CT produced the largest improvement (MD = 3.18 cm, 95% CI: 2.51 to 3.85; SUCRA = 82.0%), followed by PT (MD = 2.83 cm, 95% CI: 2.07 to 3.59; SUCRA = 68.0%) and ST (MD = 2.02 cm, 95% CI: 1.39 to 2.66; SUCRA = 38.3%) (**Supplementary Appendix 5**, **Supplementary Figure 5.1**; **Supplementary Appendix 6**, **Supplementary Figure 6.2**). In direct comparisons, CT was significantly more effective than ST (MD = 1.16 cm, 95% CI: 0.29 to 2.03), while differences among the other active interventions were not statistically significant (**Supplementary Appendix 7**, **Supplementary Table 7.2**). For standing long jump (SLJ; 14 RCTs, n = 391), the network included six intervention nodes. All active interventions showed a favorable direction of effect compared with CON (**Supplementary Appendix 5**, **Supplementary Figure 5.2**). However, only PT produced a statistically significant improvement in SLJ performance (MD = 11.93 cm, 95% CI: 1.35 to 22.52; SUCRA = 64.0%). Although NMT ranked highest, its effect was not statistically significant (Appendix 5, **Supplementary Figure 5.2**; **Supplementary Appendix 6**, **Supplementary Figure 6.3**). No significant differences were found between active interventions, suggesting that their relative effects on SLJ were broadly comparable (**Supplementary Appendix 7**, **Supplementary Table 7.3**).

### 3.4 Linear sprint performance

**Figure 4:**
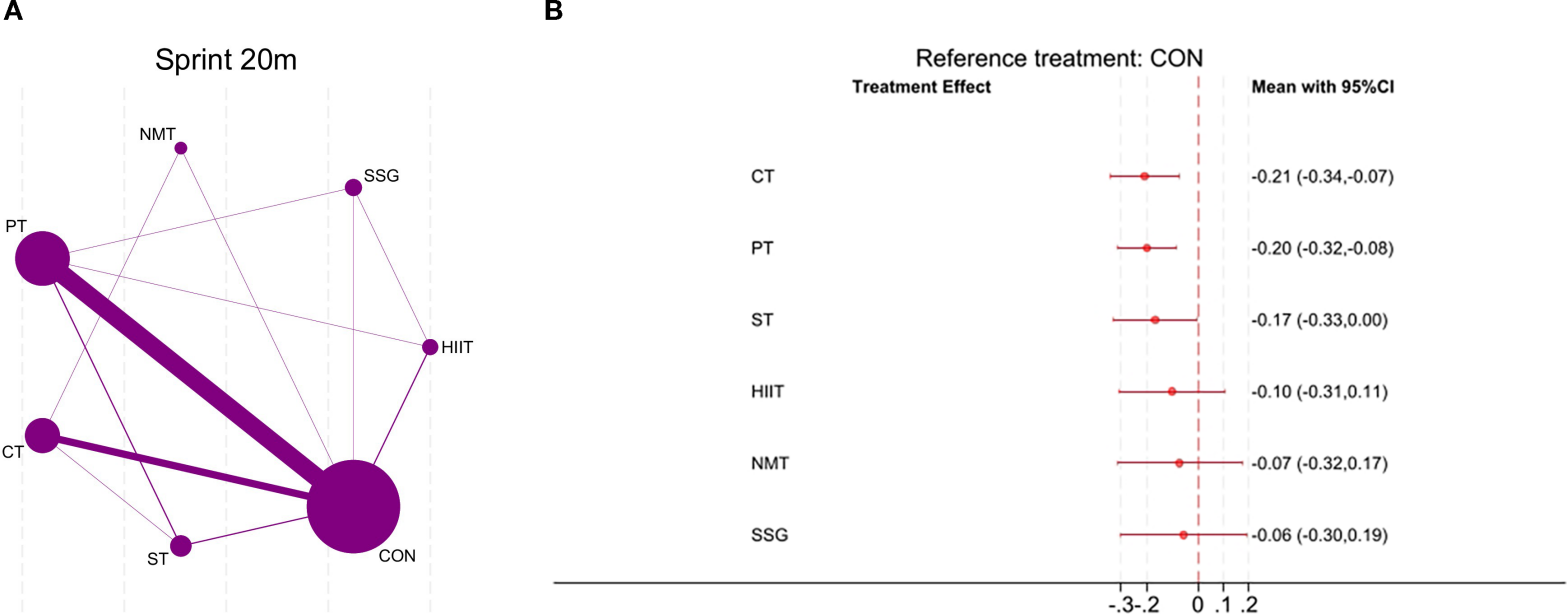
Network and forest plots for the 20-m sprint outcome. **(A)** The network plot shows the direct comparisons among training interventions for the 20-m sprint outcome, including CON, PT, CT, ST, NMT, SSG, and HIIT. Node size reflects the total number of participants assigned to each intervention, and line thickness indicates the number of participants included in each direct comparison. **(B)** The forest plot shows the effect estimates and 95% confidence intervals for each intervention versus CON in the network meta-analysis. Effect estimates below 0 favor the intervention listed on the left. Interventions are presented in sequence to support clear interpretation of their relative effects.

Across the three linear sprint outcomes, PT and CT showed the most consistent improvements. For the 20 m sprint (20 RCTs, n = 579), the evidence network included seven intervention nodes, with CON as the main comparator. The strongest direct evidence came from PT versus CON. All exercise interventions tended to reduce sprint time, but only CT and PT reached statistical significance (**Figure 4**). CT produced the largest improvement (MD = −0.21 s, 95% CI: −0.34 to −0.07; SUCRA = 78.8%; low-certainty evidence), followed closely by PT (MD = −0.20 s, 95% CI: −0.32 to −0.08; SUCRA = 76.5%; moderate-certainty evidence). Although ST ranked third, its effect was not statistically significant (**Figure 4**; **Supplementary Appendix 6**, **Supplementary Figure 6.5**). Direct comparisons showed no significant differences between exercise interventions (**Supplementary Appendix 7**, **Supplementary Table 7.5**). Most comparisons were rated as low or very low certainty, mainly because of imprecision and differences between studies (**Supplementary Appendix 8**, **Supplementary Table 8.3**). For the 10 m sprint (19 RCTs, n = 622), the network included seven intervention nodes, with PT and CT as key comparators. SSG ranked highest, but its effect was not statistically significant. PT ranked second (MD = −0.10 s, 95% CI: −0.16 to −0.03; SUCRA = 67.7%), followed closely by CT (MD = −0.10 s, 95% CI: −0.15 to −0.04; SUCRA = 67.4%) (**Supplementary Appendix 5**, **Supplementary Figure 5.3**; **Supplementary Appendix 6**, **Supplementary Figure 6.4**). No significant differences were found between exercise interventions in direct comparisons (**Supplementary Appendix 7**, **Supplementary Table 7.4**). For the 30 m sprint (13 RCTs, n = 419), the network included six intervention nodes. CT versus CON was the most frequent direct comparison. However, PT produced the largest improvement in sprint time (MD = −0.67 s, 95% CI: −1.03 to −0.30; SUCRA = 93.2%), followed by ST (MD = −0.40 s, 95% CI: −0.72 to −0.09; SUCRA = 64.2%). CT ranked third, but its effect was not statistically significant compared with CON (**Supplementary Appendix 5**, **Supplementary Figure 5.4**; **Supplementary Appendix 6**, **Supplementary Figure 6.6**). As with the 10 m and 20 m outcomes, direct comparisons showed no significant differences between exercise interventions (**Supplementary Appendix 7**, **Supplementary Table 7.6**).

### 3.5 Change-of-direction performance

**Figure 5:**
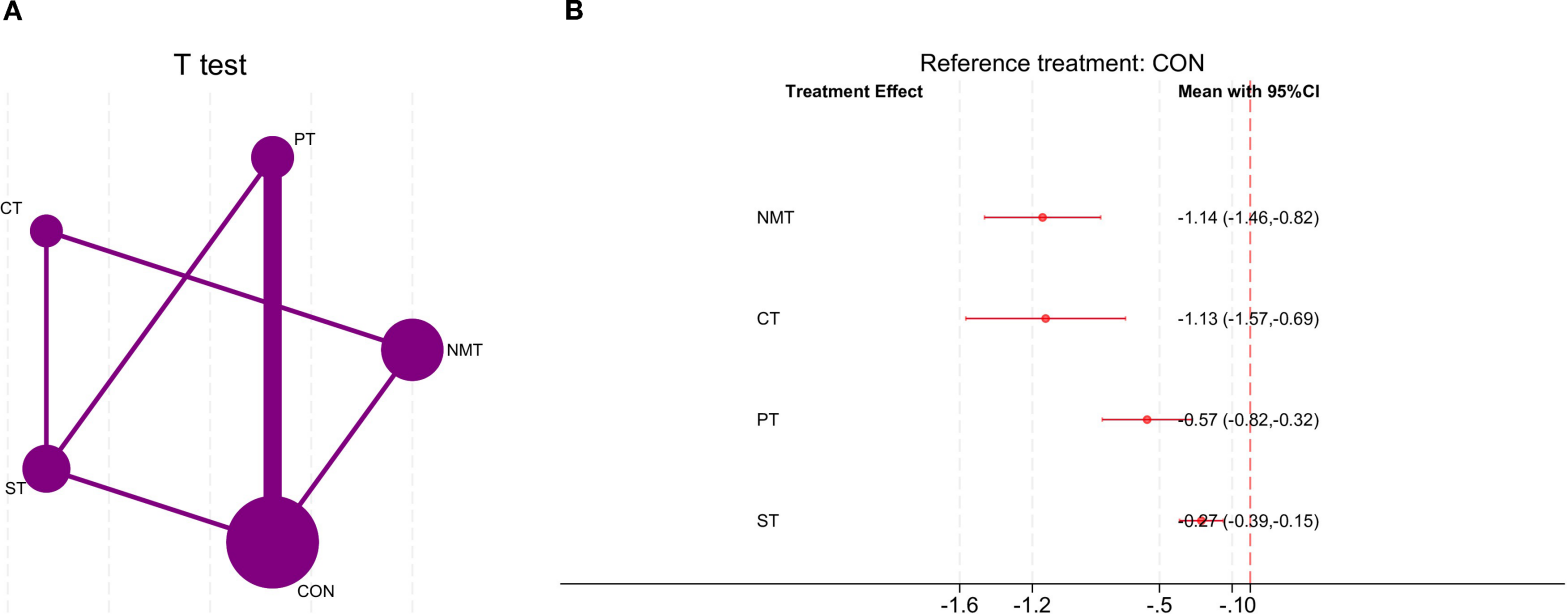
Network and forest plots for the T-test outcome. **(A)** The network plot shows the direct comparisons among training interventions for the T-test outcome, including CON, PT, CT, ST, and NMT. Node size reflects the total number of participants assigned to each intervention, and line thickness indicates the number of participants included in each direct comparison. **(B)** The forest plot presents the effect estimates and 95% confidence intervals for each intervention versus CON in the network meta-analysis. Effect estimates below 0 favor the intervention listed on the left. The interventions are presented in sequence to support clear interpretation of their relative effects.

The effects of the interventions varied across the three change-of-direction tests. For the T test (7 RCTs, n = 187), the evidence network included five intervention nodes, with CON as the main comparator. The most frequent direct comparison was between PT and CON (**Supplementary Figure 5.8**). All exercise interventions significantly improved T test performance (**Figure 5**). NMT showed the largest improvement (MD = −1.14 s, 95% CI: −1.46 to −0.82; SUCRA = 88.4%; moderate-certainty evidence), followed closely by CT (MD = −1.13 s, 95% CI: −1.57 to −0.69; SUCRA = 86.1%; high-certainty evidence). PT also produced a significant benefit, although the effect was smaller (MD = −0.57 s, 95% CI: −0.82 to −0.32; SUCRA = 50.2%; low-certainty evidence) (**Figure 5**; **Supplementary Appendix 6**, **Supplementary Figure 6.8**). Direct comparisons showed that NMT and CT were both more effective than PT, and that NMT, CT, and PT were all more effective than ST (**Supplementary Appendix 7**, **Supplementary Table 7.7**). Overall, the certainty of evidence was mainly low to moderate (**Supplementary Appendix 8**, **Supplementary Table 8.4**). For the modified T test (7 RCTs, n = 266), the network also included five intervention nodes. PT produced the greatest improvement (MD = −0.94 s, 95% CI: −1.48 to −0.40; SUCRA = 95.8%), followed by CT (MD = −0.58 s, 95% CI: −0.79 to −0.38; SUCRA = 72.9%). ST ranked third, but its effect was not statistically significant (MD = −0.38 s, 95% CI: −0.77 to 0.01; SUCRA = 53.5%) (**Supplementary Appendix 6**, **Supplementary Figure 6.7**). In direct comparisons, both PT and CT were significantly more effective than NMT (**Supplementary Appendix 7**, **Supplementary Table 7.8**). For the Illinois Agility Test (9 RCTs, n = 281), the evidence again formed a five-node network. CT showed the largest improvement (MD = −0.94 s, 95% CI: −1.29 to −0.60; SUCRA = 91.0%), followed by PT (MD = −0.84 s, 95% CI: −1.24 to −0.43; SUCRA = 82.3%) (**Supplementary Appendix 5**, **Supplementary Figure 5.6**; Appendix 6, **Supplementary Figure 6.9**). Direct comparisons further showed significant differences between PT and NMT, CT and NMT, and CT and ST (**Supplementary Appendix 7**, **Supplementary Table 7.9**).

### 3.6 Sensitivity analyses

After excluding all studies rated as high risk of bias under the RoB 2 assessment, the results of the sensitivity analyses for CMJ, the 20-m sprint, and the T test remained largely consistent with the main analysis, supporting the robustness of the primary findings. For CMJ, CT and PT still showed the greatest improvements. For the 20-m sprint, CT and PT remained significantly superior, while ST also reached significance in the sensitivity analysis. Similarly, for the T test, the beneficial effects of NMT, CT, PT, and ST were all maintained (**Supplementary Appendix 10**, **Supplementary Table 10.1**).

### 3.7 Subgroup analyses

#### 3.7.1 By intervention duration

The effects of exercise interventions may depend on the length of the training period. Previous evidence indicates that early improvements in athletic performance are largely driven by neural adaptation, whereas more stable structural changes in muscles and tendons generally require longer training exposure, typically beyond 8 weeks (Kubo et al., 2010; Damas et al., 2018). To examine this, we grouped the studies into interventions lasting ≤8 weeks and those lasting >8 weeks. Overall, longer interventions produced greater improvements and were more likely to show statistically significant effects for CMJ, 20-m sprint, and T test performance. For example, compared with the control group, CT improved CMJ by a mean difference of 3.26 cm (95% CI: 0.18 to 6.35) in interventions lasting ≤8 weeks. In interventions lasting >8 weeks, this effect increased substantially to 9.03 cm (95% CI: 3.69 to 14.38).

A similar pattern was observed for the 20-m sprint and T test. For most interventions, including CT, PT, and NMT, significant reductions in performance time were mainly found in the >8-week subgroup. By contrast, most interventions in the ≤8-week subgroup did not show statistically significant effects. Together, these findings suggest that longer intervention periods are more effective for improving sprint and change-of-direction performance (**Supplementary Appendix 11**, **Supplementary Table 11.1**).

#### 3.7.2 By sport-specific biomechanical characteristics

Because the included team sports differ in their movement demands, we also grouped them into jump- and landing-dominant sports (e.g., basketball and volleyball) and multidirectional movement- and change-of-direction-dominant sports (e.g., soccer, handball, and rugby). This allowed us to examine whether intervention effects varied by sport type.

In jump- and landing-dominant sports, significant improvements in CMJ were found after NMT, PT, CT, and ST. In contrast, in multidirectional movement- and change-of-direction-dominant sports, significant CMJ improvements were observed only after CT and PT. For the 20-m sprint, significant improvements were found only in multidirectional movement- and change-of-direction-dominant sports, specifically after PT and CT, while no clear significant effects were found in jump- and landing-dominant sports. For the T test, NMT and CT showed the strongest effects in jump- and landing-dominant sports, whereas PT and ST produced significant improvements in multidirectional movement- and change-of-direction-dominant sports (**Supplementary Appendix 11**, **Supplementary Table 11.2**).

## 4 Discussion

This study is the first to systematically examine how different training interventions affect jumping, linear sprinting, and change-of-direction performance in adolescent female team-sport athletes. The results show that complex training (CT) and plyometric training (PT) are particularly effective for improving explosive movements in the sagittal plane, especially jumping and short-distance linear sprinting. For change-of-direction performance, neuromuscular training produced the best results in the T-test, with CT showing the next strongest effect. In contrast, PT and CT performed better in the Modified T-test and Illinois test. Taken together, these findings suggest that the most effective approach for improving change-of-direction ability depends on the specific demands of the test used to assess it. The subgroup analyses further indicate that training outcomes are shaped not only by the type of intervention, but also by training duration and the movement demands of each sport. In particular, interventions lasting more than 8 weeks may mark an important threshold, at which early neural adaptations begin to develop into more substantial physical changes. The different responses observed across sports also highlight the importance of matching training programs to both the athlete’s neuromuscular profile and the specific movement requirements of the sport.

### 4.1 Jump performance

The evidence from this study suggests that complex training (CT) and plyometric training (PT) are the most effective approaches for improving jump performance, particularly countermovement jump (CMJ). Their benefits also appear to increase with longer intervention periods, although more high-quality randomized controlled trials are still needed to confirm this pattern. These findings are consistent with earlier research in adolescent athletes showing that CT, which combines heavy resistance exercise with fast, explosive movements, generally produces greater gains in lower-limb power than a single training method alone (Zhao et al., 2026). Although previous studies have suggested that female athletes may gain less from the same training dose than males (Sánchez et al., 2020), the present results indicate that adolescent girls can still make substantial improvements in jump performance when training is maintained long enough and delivered at an appropriate intensity.

One possible explanation is that both CT and PT improve the body’s ability to produce force quickly during explosive movements (Zhao et al., 2026). PT may achieve this by repeatedly training muscles to absorb and then rapidly produce force, which can improve muscle activation and the speed of force generation (Xu et al., 2025). CT may strengthen these effects further by adding heavier resistance work, which may help athletes recruit more muscle fibers and produce greater overall force (Zhao et al., 2026). These adaptations may be especially important in adolescent female athletes, who are still undergoing important stages of neuromuscular development and may therefore respond particularly well to well-designed training.

Jump improvements also seem to depend on the movement demands of each sport. In sports such as basketball and volleyball, where jumping and landing are central, several training methods can be effective. In contrast, in sports such as soccer and handball, where movement is more varied and includes frequent directional changes, meaningful improvements were seen mainly with CT and PT. One possible reason is that athletes in these sports are regularly exposed to lateral, rotational, and single-leg movements in daily training, so further gains in vertical jump performance may require a stronger overload in straight up-and-down movements. CT and PT appear well suited to provide this stimulus and may therefore be more effective in improving vertical force production (Garcia et al., 2025). From a practical perspective, if the goal is to improve jump performance in adolescent female team-sport athletes, especially CMJ performance in sports with frequent multidirectional movement, CT should be the first choice, followed by PT. Where possible, training should last longer than 8 weeks to allow early neural changes to develop into more lasting physical adaptations in muscles and tendons (Mănescu, 2025; Xu et al., 2025).

### 4.2 Linear sprint performance

Compared with jump performance, linear sprint performance appears to require a greater training stimulus before meaningful improvements can be observed. The available evidence suggests that significant gains in 20-m sprint performance are seen mainly in the PT, CT, and ST subgroups when training lasts longer than 8 weeks. By contrast, interventions of 8 weeks or less generally do not produce clear statistical benefits, although this pattern still needs to be confirmed by high-quality randomized controlled trials. This finding is in line with earlier research showing that sprint speed can improve with training, but usually to a lesser extent than jump performance (Sánchez et al., 2020; Zhao et al., 2026). Adolescence is also considered a key period for sprint development, as young athletes tend to respond more strongly to speed training than adults, highlighting the importance of systematic intervention during this stage (Lin et al., 2025).

One possible explanation lies in the specific demands of sprinting. Short-distance sprint performance, particularly over 20 m, may depend not only on how quickly force can be produced but also on the ability to direct that force horizontally, which differs from the demands of vertical jumping (Fitzpatrick et al., 2019). As a result, exercises such as squats and vertical jump training may improve general lower-limb power but have limited effects on sprint performance unless they also enhance horizontal force production and rapid single-leg propulsion (Xu et al., 2025). Sprint gains may also be influenced by longer-term physical adaptations, including changes in the muscles and tendons of the posterior chain and improvements in rapid force transfer. Because these adaptations may be less likely to occur over a short period, sprint performance appears to benefit more from sustained training (Xu et al., 2025; Zhao et al., 2026).

The present study also found that meaningful sprint improvements were concentrated in the CT and PT groups within sports characterized by frequent multidirectional movement and changes of direction, while no clear gains were observed in sports dominated by jumping and landing. This pattern suggests that sport-specific experience may affect how efficiently strength gains transfer to sprint performance. Athletes in sports such as soccer and handball are regularly exposed to high-speed running, uneven support positions, and single-leg propulsion tasks, which may help them convert strength and power gains into forward acceleration more effectively. In contrast, basketball and volleyball athletes are more accustomed to bilateral vertical force production and may have a less developed base for single-leg horizontal propulsion and pelvic control, which may reduce the transfer of force to sprinting (Garcia et al., 2025). From a practical perspective, improving linear sprint performance may require more than vertically oriented training alone. CT and PT programs should therefore include horizontally focused exercises, such as resisted sprinting and horizontal jumping, and should ideally last longer than 8 weeks to improve transfer to sprint performance (Gołaś et al., 2024).

### 4.3 Change-of-direction performance

The available evidence suggests that change-of-direction performance in adolescent female team-sport athletes is highly task-specific, and no single training method is consistently superior across all tests. Neuromuscular training (NMT) showed the strongest effect in the T-test, plyometric training (PT) performed best in the Modified T-test, and complex training (CT) ranked highest in the Illinois test while also showing relatively consistent effects across different assessments. These findings suggest that change-of-direction ability is not a single physical quality, but a complex performance outcome influenced by the movement pattern, turning angle, braking demands, and re-acceleration requirements of each test.

This interpretation also helps explain why earlier studies may have overlooked important differences by combining results from multiple agility or change-of-direction tests into a single analysis (Lin et al., 2025). Recent research suggests that shorter, lower-angle directional changes rely more on speed, whereas larger-angle cuts and repeated braking tasks depend more on braking strength and body control (Falch et al., 2020). The present findings are consistent with this view. PT appears to be more effective for tasks that require rapid ground contact and quick re-acceleration, NMT seems better suited to tasks that involve greater control during deceleration and turning, and CT appears to transfer more consistently across tasks because it develops both strength and speed.

These differences may also be supported by the likely effects of each training method. NMT may improve performance by enhancing balance, movement control, and coordination across the trunk and lower limbs, which may be especially useful in the traditional T-test, where lateral movement, sudden stops, and backward running are required (Chen et al., 2025a). PT may be more effective in short, repeated start-stop tasks because it may improve the ability to absorb and rapidly reproduce force (Lin et al., 2025). CT, by combining heavy resistance work with explosive movement, may improve both braking and propulsive force, which may explain its more stable performance in the Illinois test, a longer and more complex task (Thapa et al., 2024). In adolescent girls, these task-specific responses may be even more pronounced because ongoing growth and maturation may affect coordination and movement control (Retzepis et al., 2025).

Taken together, these findings suggest that change-of-direction training should be matched to the specific demands of the sport rather than applied uniformly. NMT may be most appropriate for situations that emphasize defensive shuffling, stopping, and body control. PT may be more effective when the goal is to improve short-distance separation and rapid re-acceleration. When athletes need to perform repeated, high-intensity directional changes across multiple angles, CT appears to be the most suitable primary training strategy.

### 4.4 Training duration and sport-specific demands

Another important finding of this study is that both training duration and sport-specific demands appear to shape training outcomes. Across jumping, sprinting, and change-of-direction performance, interventions lasting longer than 8 weeks were generally more likely to produce stable and meaningful improvements. This suggests that adaptation in adolescent female athletes involves more than short-term gains in muscle activation. Instead, it likely requires sufficient time for early neural changes to develop into broader physical adaptations. This seems especially important for sprinting and complex directional changes, which may depend not only on effective neural drive but also on longer-term changes in tendon properties, muscle structure, braking capacity, and movement coordination (Jurišić et al., 2021; Lin et al., 2025; Xu et al., 2025).

The findings also support the importance of matching training to the movement demands of the sport. Training programs should reflect the main physical requirements of each discipline rather than follow a single general model. In sports dominated by jumping and landing, NMT may be useful for improving multidirectional control, while CT and PT may further strengthen vertical power. In contrast, in sports characterized by frequent multidirectional movement and directional changes, CT, PT, or ST may be more directly effective for improving explosive performance and change-of-direction ability. Compared with a uniform approach, this more targeted method is likely to have greater practical value.

### 4.5 Potential influence of maturational status on training adaptation

Only 13 of the 47 randomized controlled trials included in this study reported information on maturational status, and the methods used were inconsistent. These included pre-, mid-, and post-peak height velocity (PHV), maturity offset, age at PHV (APHV), and years from PHV (YPHV). In addition, these measures were unevenly distributed across intervention types and outcomes, making it difficult to form sufficiently large and comparable subgroups. For this reason, no subgroup analysis based on maturational status was performed.

Even so, PHV remains a useful framework for interpreting differences in training response. These explanations should be interpreted as evidence-informed hypotheses rather than mechanisms directly tested in this network meta-analysis. According to the synergistic adaptation model, training effects may be greater when exercise coincides with a key period of development in stretch-shortening cycle function during puberty (McGarrigal et al., 2025). This may be particularly relevant in girls. Although females typically reach PHV earlier than males, at an average age of about 11.18 years, this stage is not usually accompanied by the same natural increase in neuromuscular function often seen in boys. Instead, it is more commonly associated with a relative increase in fat mass, slower muscle development, and greater compliance of connective tissue (Lima et al., 2024; McGarrigal et al., 2025). This may help explain why longer CT and PT interventions produced more consistent gains in jumping and sprinting performance in the present study, possibly because sustained mechanical loading improves lower-limb stiffness, enhances use of the stretch-shortening cycle, and supports broader neuromuscular adaptation (Tumkur Anil Kumar et al., 2021; Van Hooren et al., 2024; McGarrigal et al., 2025; Zheng et al., 2025).

By contrast, at earlier stages of maturation, the central nervous system may be highly adaptable even though the skeletal and muscular systems are not yet fully prepared for heavy loading. Under these conditions, NMT or low-intensity PT may be more appropriate for improving landing control, early muscle activation strategies, and basic agility. This may also explain why some short-term interventions were still able to produce measurable improvements in change-of-direction performance (Cho and Shin, 2021; Sánchez Pastor et al., 2023).

### 4.6 Strengths and limitations

A major strength of this study is its use of a network meta-analysis, which made it possible to integrate both direct and indirect evidence and compare the relative effects of CT, PT, NMT, ST, HIIT, and SSG across multiple performance outcomes. Compared with traditional pairwise meta-analysis, this approach offers a stronger basis for ranking training strategies and identifying practical priorities. Another strength is the focus on adolescent female athletes, a population that remains underrepresented in the literature. By also examining training duration and sport-specific biomechanical demands in subgroup analyses, this study improves the practical relevance of its findings.

However, this study has several limitations. First, the overall certainty of evidence across the included studies was low to moderate. Several studies had clear weaknesses in allocation concealment, blinding, and sample size. Substantial heterogeneity was also observed for some outcomes, particularly CMJ and SLJ, which may reduce the robustness of the effect estimates. Although subgroup analyses were conducted, they did not fully explain this heterogeneity. This suggests that residual confounding may remain, including differences in baseline performance, training intensity, total training volume, biological maturity, and testing procedures.

Second, most studies did not adequately account for biological maturation, and adolescence was usually defined by chronological age rather than by indicators such as skeletal age or peak height velocity (PHV). This is important because adolescent girls often experience rapid skeletal growth, increased ligament laxity, pelvic changes, and temporary reductions in neuromuscular control around PHV. These developmental changes, sometimes described as “adolescent awkwardness,” may strongly influence both training response and injury risk (Retzepis et al., 2025). As a result, the same training program may have very different effects depending on the athlete’s stage of maturation. Athletes in the later stages of PHV may respond more positively, whereas those near peak maturation may show smaller gains or even greater risk (Albaladejo-Saura et al., 2021; Parry et al., 2024). Future research should therefore include more precise measures of biological maturation to better distinguish training effects from normal growth-related changes.

Third, this study included only performance-based outcomes measured by time or distance. Therefore, the biomechanical and neurophysiological explanations discussed in this review should be interpreted as plausible hypotheses based on previous literature, rather than as mechanisms directly tested by the present data.

Finally, because the number of available studies remains limited, some outcomes were affected by a sparse network and fragile network connectivity. For example, in the T-test network, direct comparative evidence between certain interventions was very limited, and some comparisons were supported by single-study links. This may increase the risk of small-study influence. In addition, to allow subgroup analyses, sports were grouped using a broad sport classification based on their main movement characteristics rather than absolute sport-specific characteristics. This approach may have introduced classification bias.

## 5 Conclusion

This study shows that different training methods have distinct effects on jump performance, linear sprinting, and change-of-direction ability in adolescent female team-sport athletes. CT and PT appear to be the most effective methods for improving explosive performance, especially jumping and linear sprinting. For change-of-direction tasks, NMT seems most suitable when postural control is the main demand, PT appears more effective for rapid re-acceleration, and CT may be the best option for more complex and sustained directional changes.

The findings also show that training outcomes are shaped by both intervention duration and sport-specific movement demands. Programs lasting longer than 8 weeks, together with training that matches the specific demands of the sport, should therefore be considered key principles when designing interventions for this population. In addition, SUCRA rankings should not be used alone to determine whether one intervention is better than another. They should be interpreted alongside direct comparison evidence, effect sizes, 95% confidence intervals, and CINeMA ratings of evidence certainty. Nevertheless, the findings should be interpreted with caution because the current evidence base is only low to moderate in quality and often lacks adequate control for biological maturation. Future high-quality randomized controlled trials should incorporate maturity-related indicators such as PHV to help refine individualized training strategies for adolescent female team-sport athletes.

## Data Availability

The original contributions presented in the study are included in the article/**Supplementary Material**. Further inquiries can be directed to the corresponding author.

## References

[B1] Albaladejo-Saura M. Vaquero-Cristobal R. Gonzalez-Galvez N. Esparza-Ros F. (2021). Relationship between biological maturation, physical fitness, and kinanthropometric variables of young athletes: a systematic review and meta-analysis. Int. J. Environ. Res. Public Health 18, 328. doi: 10.3390/ijerph18010328 33466291 PMC7795393

[B2] Aschendorf P. F. Zinner C. Delextrat A. Engelmeyer E. Mester J. (2019). Effects of basketball-specific high-intensity interval training on aerobic performance and physical capacities in youth female basketball players. Physician Sportsmedicine 47, 65–70. doi: 10.1080/00913847.2018.1520054 30193074

[B3] Attene G. Iuliano E. Di Cagno A. Calcagno G. Moalla W. Aquino G. . (2015). Improving neuromuscular performance in young basketball players: plyometric vs. technique training. J. Sports Med. Phys. Fitness 55, 1–8. 24921611

[B4] Bouteraa I. Negra Y. Shephard R. J. Chelly M. S. (2020). Effects of combined balance and plyometric training on athletic performance in female basketball players. J. Strength Conditioning Res. 34, 1967–1973. doi: 10.1519/jsc.0000000000002546 29489714

[B5] Carmichael M. A. Thomson R. L. Moran L. J. Wycherley T. P. (2021). The impact of menstrual cycle phase on athletes’ performance: a narrative review. Int. J. Environ. Res. Public Health 18, 1667. doi: 10.3390/ijerph18041667 33572406 PMC7916245

[B6] Chaabene H. Negra Y. Moran J. Prieske O. Sammoud S. Ramirez-Campillo R. . (2021a). Plyometric training improves not only measures of linear speed, power, and change-of-direction speed but also repeated sprint ability in young female handball players. J. Strength Conditioning Res. 35, 2230–2235. doi: 10.1519/jsc.0000000000003128 30946268

[B7] Chaabene H. Negra Y. Sammoud S. Moran J. Ramirez-Campillo R. Granacher U. . (2021b). The effects of combined balance and complex training versus complex training only on measures of physical fitness in young female handball players. Int. J. Sports Physiol. Perform. 16, 1439–1446. doi: 10.1123/ijspp.2020-0765 33735832

[B8] Chen B. Deng L. Liu Y. Deng X. Yuan X. (2025a). The effect of integrative neuromuscular training on enhancing athletic performance: a systematic review and meta-analysis. Life 15, 1183. doi: 10.3390/life15081183 40868831 PMC12387185

[B9] Chen Y. Tulhongjiang M. Ling T. Feng X. Mi J. Liu R. (2025b). The optimal training intervention for improving the change of direction performance of adolescent team-sport athletes: a systematic review and network meta-analysis. PeerJ 13, e18971. doi: 10.7717/peerj.18971 39995993 PMC11849509

[B10] Cho S.-H. Shin I.-S. (2021). A reporting quality assessment of systematic reviews and meta-analyses in sports physical therapy: a review of reviews. Healthcare 9, 1368. doi: 10.3390/healthcare9101368 34683046 PMC8544369

[B11] Christofilos S. I. Tsikopoulos K. Tsikopoulos A. Kitridis D. Sidiropoulos K. Stoikos P. N. . (2022). Network meta-analyses: methodological prerequisites and clinical usefulness. World J. Method. 12, 92. doi: 10.5662/wjm.v12.i3.92 35721244 PMC9157634

[B12] Cossio-Bolaños M. A. Vidal-Espinoza R. Minango-Negrete J. Olivares P. R. Urzua-Alul L. de Campos L. F. C. C. . (2021). Estimation of pubertal growth spurt parameters in children and adolescents living at moderate altitude in Colombia. Front. Endocrinol. 12, 718292. doi: 10.3389/fendo.2021.718292 34603203 PMC8485727

[B13] Curteis T. Wigle A. Michaels C. J. Nikolakopoulou A. (2025). Ranking of treatments in network meta-analysis: incorporating minimally important differences. BMC Med. Res. Methodol. 25, 67. doi: 10.21203/rs.3.rs-5417882/v1 40065217 PMC11892231

[B14] Damas F. Libardi C. A. Ugrinowitsch C. (2018). The development of skeletal muscle hypertrophy through resistance training: the role of muscle damage and muscle protein synthesis. Eur. J. Appl. Physiol. 118, 485–500. doi: 10.1007/s00421-017-3792-9 29282529

[B15] Deng Q. C. (2020). Experimental Study on the Influence of Plyometric Training on Specific Jumping Ability of Secondary School Volleyball Players (Beijing, China: Minzu University of China). Master’s thesis.

[B16] Falch H. N. Haugen M. E. Kristiansen E. L. van den Tillaar R. (2022). Effect of strength vs. plyometric training upon change of direction performance in young female handball players. Int. J. Environ. Res. Public Health 19, 6946. doi: 10.3390/ijerph19116946 35682528 PMC9180755

[B17] Falch H. N. Rædergård H. G. van den Tillaar R. (2020). Effect of approach distance and change of direction angles upon step and joint kinematics, peak muscle activation, and change of direction performance. Front. Sports Active Living 2, 594567. doi: 10.3389/fspor.2020.594567 33345172 PMC7739774

[B18] Fernandes T. Rago V. Castañer M. Camerino O. (2024). Ranking sports science and medicine interventions impacting team performance: a protocol for a systematic review and meta-analysis of observational studies in elite football. BMJ Open Sport Exercise Med. 10. doi: 10.1136/bmjsem-2024-002196 39286324 PMC11404162

[B19] Fitzpatrick D. A. Cimadoro G. Cleather D. J. (2019). The magical horizontal force muscle? A preliminary study examining the “force-vector” theory. Sports 7, 30. doi: 10.3390/sports7020030 30678251 PMC6409580

[B20] Fort A. Romero D. Bagur C. Guerra M. (2012). Effects of whole-body vibration training on explosive strength and postural control in young female athletes. J. Strength Conditioning Res. 26, 926–936. doi: 10.1519/jsc.0b013e31822e02a5 22446665

[B21] Gaamouri N. Hammami M. Cherni Y. Oranchuk D. J. Bragazzi N. Knechtle B. . (2023a). The effects of upper and lower limb elastic band training on the change of direction, jump, power, strength and repeated sprint ability performance in adolescent female handball players. Front. Sports Active Living 5, 1021757. doi: 10.3389/fspor.2023.1021757 36909357 PMC9992399

[B22] Gaamouri N. Hammami M. Cherni Y. Oranchuk D. J. van den Tillaar R. Chelly M. S. (2024). Rubber band training improves athletic performance in young female handball players. J. Hum. Kinetics 92, 227. doi: 10.5114/jhk/175396 38736592 PMC11079920

[B23] Gaamouri N. Hammami M. Cherni Y. Rosemann T. Knechtle B. Chelly M. S. . (2023b). The effects of 10-week plyometric training program on athletic performance in youth female handball players. Front. Sports Active Living 5, 1193026. doi: 10.3389/fspor.2023.1193026 37521098 PMC10375710

[B24] Garcia G. Bucheli R. Castillo J. T. Fernandez J. de la Torre A. Fierro A. L. . (2025). Biomechanical and neuromuscular differences between professional and varsity football players during countermovement and approach jumps. PloS One 20, e0336672. doi: 10.1371/journal.pone.0336672 41325339 PMC12668496

[B25] Gavala M. Bassa E. Zetou E. Smilios I. Douda H. (2023). Effect of integrative neuromuscular training and detraining on performance indices in young female volleyball players. J. Sports Med. Phys. Fitness 63, 1285–1294. doi: 10.23736/S0022-4707.23.15108-5 37736664

[B26] Genc H. Cigerci A. Sever O. (2019). Effect of 8-week core training exercises on physical and physiological parameters of female handball players. Phys. Educ. Students 23, 297–305. doi: 10.15561/20755279.2019.0604

[B27] Gołaś A. Pietraszewski P. Roczniok R. Terbalyan A. Maszczyk A. Opaliński R. . (2024). Effects of an 8-week pre-season targeted training on sprinting performance, agility and lower limb muscular asymmetries in elite soccer players. Biol. Sport 41, 69–76. doi: 10.5114/biolsport.2024.134754 PMC11474991 39416490

[B28] Haghighi A. H. Hosseini S. B. Askari R. Shahrabadi H. Ramirez-Campillo R. (2024). Effects of plyometric compared to high-intensity interval training on youth female basketball player’s athletic performance. Sport Sci. Health 20, 211–220. doi: 10.1007/s11332-023-01096-2 30311153

[B29] Haklı Ö. Dincer S. Sahinkaya T. Metin G. (2026). Perturbation training in young female basketball players: a randomized controlled trial. Int. J. Sports Med. 47, 42–49. doi: 10.1055/a-2655-3997 40664373

[B30] Hammami M. Gaamouri N. Aloui G. Shephard R. J. Chelly M. S. (2019a). Effects of a complex strength-training program on athletic performance of junior female handball players. Int. J. Sports Physiol. Perform. 14, 163–169. doi: 10.1123/ijspp.2018-0160 29952672

[B31] Hammami M. Gaamouri N. Cherni Y. Chelly M. S. Hill L. Knechtle B. (2022a). Effects of contrast strength training with elastic band program on sprint, jump, strength, balance and repeated change of direction in young female handball players. Int. J. Sports Sci. Coaching 17, 1147–1157. doi: 10.1177/17479541211050724

[B32] Hammami M. Gaamouri N. Suzuki K. Shephard R. J. Chelly M. S. (2020). Effects of upper and lower limb plyometric training program on components of physical performance in young female handball players. Front. Physiol. 11, 1028. doi: 10.3389/fphys.2020.01028 33013446 PMC7461999

[B33] Hammami M. Gaamouri N. Wagner H. Pagaduan J. C. Hill L. Nikolaidis P. T. . (2022b). Effects of strength training with elastic band programme on fitness components in young female handball players: a randomized controlled trial. Biol. Sport 39, 537–545. doi: 10.5114/biolsport.2022.106390 35959327 PMC9331345

[B34] Hammami M. Ramirez-Campillo R. Gaamouri N. Aloui G. Shephard R. J. Chelly M. S. (2019b). Effects of a combined upper-and lower-limb plyometric training program on high-intensity actions in female U14 handball players. Pediatr. Exercise Sci. 31, 465–472. doi: 10.1123/pes.2018-0278 31310989

[B35] Hammami M. Zmijewski P. (2024). Comparative analysis of standard and contrast elastic resistance band training effects on physical fitness in female adolescent handball players. Biol. Sport 41, 119–127. doi: 10.5114/biolsport.2024.134143 38952902 PMC11167473

[B36] Higgins J. P. T. Li T. Deeks J. J. (2024). Chapter 6: Choosing effect measures and computing estimates of effect. In: Higgins J. P. T. Thomas J. Chandler J. Cumpston M. Li T. Page M. J. ., editors. Cochrane Handbook for Systematic Reviews of Interventions Version 6.5 ed. Cochrane.

[B37] Hou Q. (2022). Study on the Influence of INT on the Jumping Ability of Adolescent Female Volleyball Players (Changsha, China: Hunan Normal University). Master’s thesis.

[B38] Idrizovic K. Gjinovci B. Sekulic D. Uljevic O. João P. V. Spasic M. . (2018). The effects of 3-month skill-based and plyometric conditioning on fitness parameters in junior female volleyball players. Pediatr. Exercise Sci. 30, 353–363. doi: 10.1123/pes.2017-0178 29478378

[B39] Idrıss M. M. Abdelkader G. Madani R. Zerf M. Bengoua A. (2022). Effect of plyometric training on improving vertical jump in female footballers (14-17 years old). Turkish J. Kinesiology 8, 37–43. doi: 10.31459/turkjkin.1108335

[B40] Jurišić M. V. Jakšić D. Trajković N. Rakonjac D. Peulić J. Obradović J. (2021). Effects of small-sided games and high-intensity interval training on physical performance in young female handball players. Biol. Sport 38, 359–366. doi: 10.5114/biolsport.2021.99327 34475619 PMC8329974

[B41] Kambitta Valappil I. N. Govindasamy K. Vasanthi G. Elayaraja M. Clark C. C. T. Parpa K. . (2026). Effects of the FIFA 11 + Program on physical fitness in youth and adult soccer players: a systematic review and meta-analysis. Sports Med. (Auckland NZ). 56 (2), 521–541. doi: 10.1007/s40279-025-02346-8 41291351 PMC12982247

[B42] Kong R. Cao L. Li D. (2025). The chronic effects of change of direction during repeated-sprint training on jumping, sprinting, and change-of-direction abilities in players: a systematic review and meta-analysis. PeerJ 13, e19416. doi: 10.7717/peerj.19416 40416610 PMC12101437

[B43] Kubo K. Ikebukuro T. Yata H. Tsunoda N. Kanehisa H. (2010). Time course of changes in muscle and tendon properties during strength training and detraining. J. Strength Conditioning Res. 24, 322–331. doi: 10.1519/jsc.0b013e3181c865e2 19996769

[B44] Lephart S. M. Abt J. Ferris C. Sell T. Nagai T. Myers J. . (2005). Neuromuscular and biomechanical characteristic changes in high school athletes: a plyometric versus basic resistance program. Br. J. Sports Med. 39, 932–938. doi: 10.1136/bjsm.2005.019083 16306502 PMC1725089

[B45] Lima A. B. Quinaud R. T. Karasiak F. C. Galvão L. G. Gonçalves C. E. Carvalho H. M. . (2024). Longitudinal meta-analysis of peak height velocity in young female athletes. Cureus 16. doi: 10.7759/cureus.59482 38826930 PMC11142863

[B46] Lin G. Zhang R. Wu K. Deng B. Shi Y. Huang W. . (2025). Effects of plyometric training on physical fitness in adolescent and adult female team sport athletes: a systematic review and meta-analysis. Front. Physiol. 16, 1639477. doi: 10.3389/fphys.2025.1639477 41049493 PMC12488710

[B47] Lindblom H. Waldén M. Hägglund M. (2012). No effect on performance tests from a neuromuscular warm-up programme in youth female football: a randomised controlled trial. Knee Surgery Sports Traumatology Arthroscopy 20, 2116–2123. doi: 10.1007/s00167-011-1846-9 22203049

[B48] Luo H. Zhu X. Nasharuddin N. A. Kamalden T. F. T. Xiang C. (2025). Effects of strength and plyometric training on vertical jump, linear sprint, and change-of-direction speed in female adolescent team sport athletes: a systematic review and meta-analysis. J. Sports Sci. Med. 24, 406. doi: 10.52082/jssm.2025.406 40469857 PMC12131141

[B49] Ma S. F. (2021). Experimental Study on the Effects of Unilateral Plyometric Training on Lower Limb Explosive Power of Adolescent Female Basketball Players (Shijiazhuang, China: Hebei Normal University). Master’s thesis.

[B50] Mănescu D. C. (2025). Computational analysis of neuromuscular adaptations to strength and plyometric training: an integrated modeling study. Sports 13, 298. doi: 10.3390/sports13090298 41003604 PMC12473730

[B51] Martel G. F. Harmer M. L. Logan J. M. Parker C. B. (2005). Aquatic plyometric training increases vertical jump in female volleyball players. Med. Sci. Sports Exercise 37, 1814–1819. doi: 10.1249/01.mss.0000184289.87574.60 16260986

[B52] Martínez-Hernández D. Jones P. A. (2024). Change of direction actions in goal scoring situations in male and female professional soccer. Int. J. Strength Conditioning 4. doi: 10.47206/ijsc.v4i1.192

[B53] Mathisen G. Danielsen K. H. (2014). Effects of speed exercises on acceleration and agility performance in 13-year-old female soccer players. J. Phys. Educ. Sport 14, 471. doi: 10.7752/jpes.2014.04071

[B54] McGarrigal L. D. Morse C. I. Sims D. T. Stebbings G. K. (2025). Development of stretch-shortening cycle function in girls during maturation and in response to training: a narrative review. J. Strength Conditioning Res. 39, e1043–e1051. doi: 10.1519/jsc.0000000000005191 40644680

[B55] Meszler B. Váczi M. (2019). Effects of short-term in-season plyometric training in adolescent female basketball players. Physiol. Int. 106, 168–179. doi: 10.1556/2060.106.2019.14 31271308

[B56] Nayıroğlu S. Yılmaz A. K. Silva A. F. Silva R. Nobari H. Clemente F. M. (2022). Effects of small-sided games and running-based high-intensity interval training on body composition and physical fitness in under-19 female soccer players. BMC Sports Sci. Med. Rehabil. 14, 119. doi: 10.1186/s13102-022-00516-z 35765044 PMC9238068

[B57] Nimphius S. Callaghan S. J. Bezodis N. E. Lockie R. G. (2018). Change of direction and agility tests: Challenging our current measures of performance. Strength Conditioning J. 40, 26–38. doi: 10.1519/ssc.0000000000000309 38604988

[B58] Noutsos K. S. Meletakos P. G. Kepesidou M. Bogdanis G. C. (2024). Equal effects of low-and moderate-volume supplementary plyometric training on sprint, change of direction ability, and lower-limb power in preadolescent female handball players. J. Funct. Morphology Kinesiology 9, 204. doi: 10.3390/jfmk9040204 39584857 PMC11586993

[B59] Nygaard Falch H. Guldteig Rædergård H. van den Tillaar R. (2019). Effect of different physical training forms on change of direction ability: a systematic review and meta-analysis. Sports Med. - Open 5, 53. doi: 10.1186/s40798-019-0223-y 31858292 PMC6923302

[B60] Ortega J. A. F. De los Reyes Y. G. Pena F. R. G. (2020). Effects of strength training based on velocity versus traditional training on muscle mass, neuromuscular activation, and indicators of maximal power and strength in girls soccer players. Apunts Sports Med. 55, 53–61. doi: 10.1016/j.apunsm.2020.03.002 38826717

[B61] Ozbar N. Ates S. Agopyan A. (2014). The effect of 8-week plyometric training on leg power, jump and sprint performance in female soccer players. J. Strength Conditioning Res. 28, 2888–2894. doi: 10.1519/jsc.0000000000000541 24852255

[B62] Paes P. P. Correia G. A. F. Damasceno V. D. O. Lucena E. V. R. Alexandre I. G. Da Silva L. R. . (2022). Effect of plyometric training on sprint and change of direction speed in young basketball athletes. J. Phys. Educ. Sport 22, 305–310. doi: 10.7752/jpes.2022.02039

[B63] Pang X. L. (2024). Study on the Influence of Plyometric Training on Foot Movement Ability of Adolescent Female Basketball Players (Harbin, China: Harbin Sport University). Master’s thesis.

[B64] Pardos-Mainer E. Casajús J. A. Bishop C. Gonzalo-Skok O. (2020). Effects of combined strength and power training on physical performance and interlimb asymmetries in adolescent female soccer players. Int. J. Sports Physiol. Perform. 15, 1147–1155. doi: 10.1123/ijspp.2019-0265 32820132

[B65] Parry G. N. Williams S. McKay C. D. Johnson D. J. Bergeron M. F. Cumming S. P. (2024). Associations between growth, maturation and injury in youth athletes engaged in elite pathways: a scoping review. Br. J. Sports Med. 58, 1001–1010. doi: 10.1136/bjsports-2024-108233 39209526 PMC11420720

[B66] Pereira A. Costa A. M. Santos P. Figueiredo T. João P. V. (2015). Training strategy of explosive strength in young female volleyball players. Medicina 51, 126–131. doi: 10.1016/j.medici.2015.03.004 25975882

[B67] Retzepis N.-O. Avloniti A. Kokkotis C. Stampoulis T. Balampanos D. Gkachtsou A. . (2025). The effect of peak height velocity on strength and power development of young athletes: A scoping review. J. Funct. Morphology Kinesiology 10, 168. doi: 10.3390/jfmk10020168 40407452 PMC12101259

[B68] Rhodes K. M. Turner R. M. Higgins J. P. (2015). Predictive distributions were developed for the extent of heterogeneity in meta-analyses of continuous outcome data. J. Clin. Epidemiol. 68, 52–60. doi: 10.1016/j.jclinepi.2014.08.012 25304503 PMC4270451

[B69] Rojano Ortega D. Berral-Aguilar A. J. Berral de la Rosa F. J. (2022). Kinetics and vertical stiffness of female volleyball players: Effect of low-intensity plyometric training. Res. Q. For. Exercise Sport 93, 734–740. doi: 10.1080/02701367.2021.1915946 34709134

[B70] Rubley M. D. Haase A. C. Holcomb W. R. Girouard T. J. Tandy R. D. (2011). The effect of plyometric training on power and kicking distance in female adolescent soccer players. J. Strength Conditioning Res. 25, 129–134. doi: 10.1519/jsc.0b013e3181b94a3d 19966586

[B71] Salaj S. Markovic G. (2011). Specificity of jumping, sprinting, and quick change-of-direction motor abilities. J. Strength Conditioning Res. 25, 1249–1255. doi: 10.1519/jsc.0b013e3181da77df 21240031

[B72] Sánchez M. Sanchez-Sanchez J. Nakamura F. Y. Clemente F. M. Romero-Moraleda B. Ramirez-Campillo R. (2020). Effects of plyometric jump training in female soccer player’s physical fitness: a systematic review with meta-analysis. Int. J. Environ. Res. Public Health 17, 8911. doi: 10.3390/ijerph17238911 33266195 PMC7731275

[B73] Sánchez Pastor A. García-Sánchez C. Marquina Nieto M. de la Rubia A. (2023). Influence of strength training variables on neuromuscular and morphological adaptations in prepubertal children: a systematic review. Int. J. Environ. Res. Public Health 20, 4833. doi: 10.3390/ijerph20064833 36981742 PMC10049541

[B74] Shui Y. Fu H. (2018). Effects of integrative neuromuscular training on specific athletic performance of adolescent female soccer players. J. Chengdu Sport Univ. 44, 84–90. doi: 10.15942/j.jcsu.2018.05.014

[B75] Su X. McDonough D. J. Chu H. Quan M. Gao Z. (2020). Application of network meta-analysis in the field of physical activity and health promotion. J. Sport Health Sci. 9, 511–520. doi: 10.1016/j.jshs.2020.07.011 32745617 PMC7749244

[B76] Tazabek Y. Zhunusbekov Z. Avsiyevich V. Belegova A. Yermenova B. (2024). The influence of physiological and functional characteristics of the body of girls aged 17-18 years on playing sports. Retos 59, 811–821. doi: 10.47197/retos.v59.105688

[B77] Thapa R. K. Weldon A. Freitas T. T. Boullosa D. Afonso J. Granacher U. . (2024). What do we know about complex-contrast training? A systematic scoping review. Sports Med. - Open 10, 104. doi: 10.1186/s40798-024-00771-z 39333341 PMC11436572

[B78] Trajković N. Bogataj Š. (2020). Effects of neuromuscular training on motor competence and physical performance in young female volleyball players. Int. J. Environ. Res. Public Health 17, 1755. doi: 10.3390/ijerph17051755 32182680 PMC7084803

[B79] Tumkur Anil Kumar N. Oliver J. L. Lloyd R. S. Pedley J. S. Radnor J. M. (2021). The influence of growth, maturation and resistance training on muscle-tendon and neuromuscular adaptations: a narrative review. Sports 9, 59. doi: 10.3390/sports9050059 34066778 PMC8150311

[B80] Váczi M. Fazekas G. Pilissy T. Cselkó A. Trzaskoma L. Sebesi B. . (2022). The effects of eccentric hamstring exercise training in young female handball players. Eur. J. Appl. Physiol. 122, 955–964. doi: 10.1007/s00421-022-04888-5 35064811 PMC8926956

[B81] Van Hooren B. Aagaard P. Blazevich A. J. (2024). Optimizing resistance training for sprint and endurance athletes: balancing positive and negative adaptations. Sports Med. 54, 3019–3050. doi: 10.1007/s40279-024-02110-4 39373864 PMC11608172

[B82] Wan K. Dai Z. Wong P. Ho R. Tam B. T. (2025). Comparing the effects of integrative neuromuscular training and traditional physical fitness training on physical performance outcomes in young athletes: a systematic review and meta-analysis. Sports Med. - Open 11, 15. doi: 10.1186/s40798-025-00811-2 39921710 PMC11807040

[B83] Wang J. Zuo Y. (2023). Effects of different directions of plyometric training on agility of adolescent female athletes. Jiujiang Vocat Tech. Coll., 77–81. doi: 10.16062/j.cnki.cn36-1247/z.2023.03.013

[B84] Wen X. Song F. Yang L. Xu Q. (2024). Small-sided soccer games promote greater adaptations on vertical jump and change-of-direction deficit and similar adaptations in aerobic capacity than high-intensity interval training in females. J. Sports Sci. Med. 23, 445. doi: 10.52082/jssm.2024.445 38841638 PMC11149063

[B85] World Rugby (2026). Coaching Women and Girls: Introduction. Available online at: https://passport.world.rugby/coaching/coaching-women-and-girls/introduction/ (Accessed April 15, 2026).

[B86] Xu T. T. (2024). Research on the Influence of SAQ Training on Special Physical Fitness of Middle School Female Volleyball Players (Chongqing, China: Southwest University). Master’s thesis.

[B87] Xu Z. Sun J. Gu J. Yu L. (2025). Effects of 8 weeks of combined strength and plyometric training on lower limb vertical stiffness and jump performance in elite long jump athletes. Front. Physiol. 16, 1692254. doi: 10.3389/fphys.2025.1692254 41293050 PMC12640820

[B88] Zhao R. Yao J. Dong Y. (2025). From a female perspective: plyometric training’s impact on jump, sprint, and change-of-direction performance in adult female athletes—a systematic review and meta-analysis. Front. Physiol. 16, 1633089. doi: 10.3389/fphys.2025.1633089 41030316 PMC12477254

[B89] Zhao C. Zhu Y. Zhang Y. (2026). Effects of combined resistance and plyometric training modalities on vertical jump and sprint: a systematic review and network meta-analysis. BMC Sports Sci. Med. Rehabil. 18, 82. doi: 10.1186/s13102-026-01531-0 41549302 PMC12903741

[B90] Zheng T. Kong R. Liang X. Huang Z. Luo X. Zhang X. . (2025). Effects of plyometric training on jump, sprint, and change of direction performance in adolescent soccer player: A systematic review with meta-analysis. PloS One 20, e0319548. doi: 10.1371/journal.pone.0319548 40300007 PMC12040276

[B91] Zhou Y. Liu J. Yang L. Zheng B. (2025). Can maturation level influence long-term physiological and physical adaptations in youth female soccer players exposed to combined sided games and HIIT? A comparison across maturation statuses. J. Sports Sci. Med. 24, 634. doi: 10.52082/jssm.2025.634 40933321 PMC12418192

