# Supplementary material for: Comparative effects of exercise-based interventions on jump, linear sprint, and change-of-direction performance in female adolescent team-sport athletes: a systematic review and network meta-analysis
Source: Front Physiol. 2026 Jul 1;17:1867361. doi: 10.3389/fphys.2026.1867361 (PMC13368477; doi:10.3389/fphys.2026.1867361)
Supplement: Supplementary file 1 [file SupplementaryFile1.docx]

Appendix 1: Search strategy

**Table S1.** **1**Search strategy of PubMed

| Searches | |
| --- | --- |
| #1 | ((((((((Female[MeSH Terms]) OR (Females[Title/Abstract])) OR (Women[MeSH Terms])) OR (Girls[MeSH Terms])) OR (Girl[MeSH Terms])) OR (Woman[MeSH Terms])) OR (Women's Groups[MeSH Terms])) OR (Women Groups[MeSH Terms])) OR (Women's Group[MeSH Terms]) |
| #2 | (((((((((Adolescent[MeSHTerms]) OR (Adolescents[Title/Abstract])) OR (Adolescence[Title/Abstract])) OR (Youth[Title/Abstract])) OR (Youths[Title/Abstract])) OR (Teens[Title/Abstract])) OR (Teen[Title/Abstract])) OR (Teenagers[Title/Abstract])) OR (Teenager[Title/Abstract])) OR (Minors[MeSH Terms]) |
| #3 | ((((((((((Athletes[MeSH Terms]) OR (Athlete[Title/Abstract])) OR (Professional Athletes[Title/Abstract])) OR (Athlete, Professional[Title/Abstract])) OR (Athletes, Professional[Title/Abstract])) OR (Professional Athlete[Title/Abstract])) OR (Elite Athletes[Title/Abstract])) OR (Athlete, Elite[Title/Abstract])) OR (Athletes, Elite[Title/Abstract])) OR (Elite Athlete[Title/Abstract])) |
| #4 | (Team Sports[MeSH Terms]) OR (Team Sport[Title/Abstract]) |
| #5 | (((((((((((((((((((((((((((((((((((((((((((Basketball[MeSH Terms])) OR (Basketballs[Title/Abstract])) OR (Netball[Title/Abstract])) OR (Netballs[Title/Abstract])) OR (Rugby[MeSH Terms])) OR (Rugby, Union Play[Title/Abstract])) OR (Play Rugby, Union[Title/Abstract])) OR (Union Play Rugbies[Title/Abstract])) OR (Union Play Rugby[Title/Abstract])) OR (Rugby, League Play[Title/Abstract])) OR (League Play Rugby[Title/Abstract])) OR (Play Rugby, League[Title/Abstract])) OR (Football[MeSH Terms])) OR (American Football[Title/Abstract])) OR (Football, American[Title/Abstract])) OR (Gaelic Football[MeSH Terms])) OR (Football, Gaelic[Title/Abstract])) OR (Ladies Gaelic Football[Title/Abstract])) OR (Football, Ladies Gaelic[Title/Abstract])) OR (Gaelic Football, Ladies[Title/Abstract])) OR (Soccer[MeSH Terms])) OR (Football European[Title/Abstract])) OR (European, Football[Title/Abstract])) OR (Europeans, Football[Title/Abstract])) OR (European Football[Title/Abstract])) OR (Football, European[Title/Abstract])) OR (Volleyball[MeSH Terms])) OR (Volleyballs[Title/Abstract])) OR (Cricket Sport[MeSH Terms])) OR (Cricket Sports[Title/Abstract])) OR (Sport, Cricket[Title/Abstract])) OR (Sports, Cricket[Title/Abstract])) OR (handball[Title/Abstract])) OR (Hockey[MeSH Terms])) OR (Hockeys[Title/Abstract])) OR (Field Hockey[Title/Abstract])) OR (Field Hockeys[Title/Abstract])) OR (Hockey, Field[Title/Abstract])) OR (Hockeys, Field[Title/Abstract])) OR (Ice Hockey[Title/Abstract])) OR (Hockey, Ice[Title/Abstract])) OR (Hockeys, Ice[Title/Abstract])) OR (Ice Hockeys[Title/Abstract]) |
| #6 | (((((((((((((((((((((((((((((((((((((((((((((((((((((((((((((((((((((((((((((((((((((((((((((((((((((((Exercise[MeSH Terms]) OR (Exercises[Title/Abstract])) OR (Exercise, Physical[Title/Abstract])) OR (Exercises, Physical[Title/Abstract])) OR (Physical Exercise[Title/Abstract])) OR (Physical Exercises[Title/Abstract])) OR (Exercise, Isometric[Title/Abstract])) OR (Exercises, Isometric[Title/Abstract])) OR (Isometric Exercises[Title/Abstract])) OR (Isometric Exercise[Title/Abstract])) OR (Exercise, Aerobic[Title/Abstract])) OR (Aerobic Exercise[Title/Abstract])) OR (Aerobic Exercises[Title/Abstract])) OR (Exercises, Aerobic[Title/Abstract])) OR (Exercise Training[Title/Abstract])) OR (Exercise Trainings[Title/Abstract])) OR (Training, Exercise[Title/Abstract])) OR (Trainings, Exercise[Title/Abstract])) OR (Physical Activity[Title/Abstract])) OR (Activities, Physical[Title/Abstract])) OR (Physical Activities[Title/Abstract])) OR (Active Breaks[Title/Abstract])) OR (Activity Breaks[Title/Abstract])) OR (Acute Exercise[Title/Abstract])) OR (Acute Exercises[Title/Abstract])) OR (Exercise, Acute[Title/Abstract])) OR (Exercises, Acute[Title/Abstract])) OR (Physical Education and Training[MeSH Terms])) OR (Physical Education, Training[Title/Abstract])) OR (Physical Education[Title/Abstract])) OR (Education, Physical[Title/Abstract])) OR (Resistance Training[MeSH Terms])) OR (Training, Resistance[Title/Abstract])) OR (Strength Training[Title/Abstract])) OR (Training, Strength[Title/Abstract])) OR (Weight-Lifting Strengthening Program[Title/Abstract])) OR (Strengthening Programs, Weight-Lifting[Title/Abstract])) OR (Strengthening Program, Weight-Lifting[Title/Abstract])) OR (Weight Lifting Strengthening Program[Title/Abstract])) OR (Weight-Lifting Strengthening Programs[Title/Abstract])) OR (Weight-Lifting Exercise Program[Title/Abstract])) OR (Exercise Programs, Weight-Lifting[Title/Abstract])) OR (Exercise Program, Weight-Lifting[Title/Abstract])) OR (Weight Lifting Exercise Program[Title/Abstract])) OR (Weight-Lifting Exercise Programs[Title/Abstract])) OR (Weight-Bearing Strengthening Program[Title/Abstract])) OR (Strengthening Programs, Weight-Bearing[Title/Abstract])) OR (Strengthening Program, Weight-Bearing[Title/Abstract])) OR (Weight Bearing Strengthening Program[Title/Abstract])) OR (Weight-Bearing Strengthening Programs[Title/Abstract])) OR (Weight-Bearing Exercise Program[Title/Abstract])) OR (Exercise Programs, Weight-Bearing[Title/Abstract])) OR (Exercise Program, Weight-Bearing[Title/Abstract])) OR (Weight Bearing Exercise Program[Title/Abstract])) OR (Weight-Bearing Exercise Programs[Title/Abstract])) OR (Plyometric Exercise[MeSH Terms])) OR (Exercise, Plyometric[Title/Abstract])) OR (Exercises, Plyometric[Title/Abstract])) OR (Plyometric Exercises[Title/Abstract])) OR (Plyometric Training[Title/Abstract])) OR (Plyometric Trainings[Title/Abstract])) OR (Training, Plyometric[Title/Abstract])) OR (Trainings, Plyometric[Title/Abstract])) OR (Plyometric Drill[Title/Abstract])) OR (Drill, Plyometric[Title/Abstract])) OR (Drills, Plyometric[Title/Abstract])) OR (Plyometric Drills[Title/Abstract])) OR (Stretch-Shortening Exercise[Title/Abstract])) OR (Exercises, Stretch-Shortening[Title/Abstract])) OR (Exercise, Stretch-Shortening[Title/Abstract])) OR (Stretch Shortening Exercise[Title/Abstract])) OR (Stretch-Shortening Exercises[Title/Abstract])) OR (Stretch-Shortening Drill[Title/Abstract])) OR (Drills, Stretch-Shortening[Title/Abstract])) OR (Drill, Stretch-Shortening[Title/Abstract])) OR (Stretch Shortening Drill[Title/Abstract])) OR (Stretch-Shortening Drills[Title/Abstract])) OR (Stretch-Shortening Cycle Exercise[Title/Abstract])) OR (Cycle Exercises, Stretch-Shortening[Title/Abstract])) OR (Cycle Exercise, Stretch-Shortening[Title/Abstract])) OR (Exercises, Stretch-Shortening Cycle[Title/Abstract])) OR (Exercise, Stretch-Shortening Cycle[Title/Abstract])) OR (Stretch Shortening Cycle Exercise[Title/Abstract])) OR (Stretch-Shortening Cycle Exercises[Title/Abstract])) OR (High-Intensity Interval Training[MeSH Terms])) OR (High Intensity Interval Training[Title/Abstract])) OR (High-Intensity Interval Trainings[Title/Abstract])) OR (Interval Training, High-Intensity[Title/Abstract])) OR (Interval Trainings, High-Intensity[Title/Abstract])) OR (Training, High-Intensity Interval[Title/Abstract])) OR (Trainings, High-Intensity Interval[Title/Abstract])) OR (High-Intensity Intermittent Exercise[Title/Abstract])) OR (Exercise, High-Intensity Intermittent[Title/Abstract])) OR (Exercises, High-Intensity Intermittent[Title/Abstract])) OR (High-Intensity Intermittent Exercises[Title/Abstract])) OR (Sprint Interval Training[Title/Abstract])) OR (Sprint Interval Trainings[Title/Abstract])) OR (small sided game[Title/Abstract])) OR (neuromuscular training[Title/Abstract])) OR (neuromuscular conditioning[Title/Abstract])) OR (complex training[Title/Abstract])) OR (contrast training[Title/Abstract])) OR (agility[Title/Abstract])) OR (sprint[Title/Abstract]) |
| #7 | (((((((((((Athletic Performance[MeSH Terms]) OR (Athletic Performances[Title/Abstract])) OR (Performance, Athletic[Title/Abstract])) OR (Performances, Athletic[Title/Abstract])) OR (Sports Performance[Title/Abstract])) OR (Performance, Sports[Title/Abstract])) OR (Performances, Sports[Title/Abstract])) OR (Sports Performances[Title/Abstract])) OR (jump[Title/Abstract])) OR (change of direction[Title/Abstract])) OR (speed[Title/Abstract])) OR (COD[Title/Abstract]) |
| #8 | ((((Randomized Controlled Trial [Publication Type]) OR (Randomized Controlled Trials as Topic[MeSH Terms])) OR (Clinical Trials, Randomized[Title/Abstract])) OR (Trials, Randomized Clinical[Title/Abstract])) OR (Controlled Clinical Trials, Randomized[Title/Abstract]) |
| #9 | #1 AND #2 AND #3 AND (#4 OR #5) AND #6 AND #7 AND #8 |

**Table S S1.2** Search strategy of Web of Science

|  | Searches |
| --- | --- |
| #1 | TS=(female* OR woman OR women OR girl* OR "women's group*" OR "women group*") |
| #2 | TS=(adolescent* OR adolescence OR youth* OR teen* OR minor*) |
| #3 | TS=(athlete* OR "professional athlete*" OR "elite athlete*") |
| #4 | TS=("team sport*") |
| #5 | TS=(basketball* OR netball* OR rugby OR football OR soccer OR volleyball* OR cricket OR handball* OR hockey) |
| #6 | TS=(exercise* OR "physical activit*" OR "active break*" OR "physical education" OR "resistance training" OR "strength training" OR "weight-lifting" OR "weight lifting" OR "weight-bearing" OR plyometric* OR "stretch-shortening*" OR "high-intensity interval training" OR HIIT OR "high-intensity intermittent exercise" OR "sprint interval training" OR SIT OR "small sided game*" OR "small-sided game*" OR "neuromuscular training" OR "neuromuscular conditioning" OR "complex training" OR "contrast training" OR agility OR sprint*) |
| #7 | TS=("athletic performance*" OR "sport* performance*" OR jump* OR "change of direction" OR COD OR speed OR sprint*) |
| #8 | TS=("randomized controlled trial*" OR "randomised controlled trial*" OR "randomized clinical trial*" OR "randomised clinical trial*" OR "controlled clinical trial*") |
| #9 | #1 AND #2 AND #3 AND (#4 OR #5) AND #6 AND #7 AND #8 |

**Table S S1.3** Search strategy of Cochrane

|  | Searches |
| --- | --- |
| #1 | [mh "Female"] OR "Females":ti,ab,kw OR [mh "Women"] OR [mh "Girls"] OR [mh "Girl"] OR [mh "Woman"] OR [mh "Women's Groups"] OR [mh "Women Groups"] OR [mh "Women's Group"] |
| #2 | [mh "Adolescent"] OR "Adolescents":ti,ab,kw OR "Adolescence":ti,ab,kw OR "Youth":ti,ab,kw OR "Youths":ti,ab,kw OR "Teens":ti,ab,kw OR "Teen":ti,ab,kw OR "Teenagers":ti,ab,kw OR "Teenager":ti,ab,kw OR [mh "Minors"] |
| #3 | [mh "Athletes"] OR "Athlete":ti,ab,kw OR "Professional Athletes":ti,ab,kw OR "Athlete, Professional":ti,ab,kw OR "Athletes, Professional":ti,ab,kw OR "Professional Athlete":ti,ab,kw OR "Elite Athletes":ti,ab,kw OR "Athlete, Elite":ti,ab,kw OR "Athletes, Elite":ti,ab,kw OR "Elite Athlete":ti,ab,kw |
| #4 | [mh "Team Sports"] OR "Team Sport":ti,ab,kw |
| #5 | [mh "Basketball"] OR "Basketballs":ti,ab,kw OR "Netball":ti,ab,kw OR "Netballs":ti,ab,kw OR [mh "Rugby"] OR "Rugby, Union Play":ti,ab,kw OR "Play Rugby, Union":ti,ab,kw OR "Union Play Rugbies":ti,ab,kw OR "Union Play Rugby":ti,ab,kw OR "Rugby, League Play":ti,ab,kw OR "League Play Rugby":ti,ab,kw OR "Play Rugby, League":ti,ab,kw OR [mh "Football"] OR "American Football":ti,ab,kw OR "Football, American":ti,ab,kw OR [mh "Gaelic Football"] OR "Football, Gaelic":ti,ab,kw OR "Ladies Gaelic Football":ti,ab,kw OR "Football, Ladies Gaelic":ti,ab,kw OR "Gaelic Football, Ladies":ti,ab,kw OR [mh "Soccer"] OR "Football European":ti,ab,kw OR "European, Football":ti,ab,kw OR "Europeans, Football":ti,ab,kw OR "European Football":ti,ab,kw OR "Football, European":ti,ab,kw OR [mh "Volleyball"] OR "Volleyballs":ti,ab,kw OR [mh "Cricket Sport"] OR "Cricket Sports":ti,ab,kw OR "Sport, Cricket":ti,ab,kw OR "Sports, Cricket":ti,ab,kw OR "handball":ti,ab,kw OR [mh "Hockey"] OR "Hockeys":ti,ab,kw OR "Field Hockey":ti,ab,kw OR "Field Hockeys":ti,ab,kw OR "Hockey, Field":ti,ab,kw OR "Hockeys, Field":ti,ab,kw OR "Ice Hockey":ti,ab,kw OR "Hockey, Ice":ti,ab,kw OR "Hockeys, Ice":ti,ab,kw OR "Ice Hockeys":ti,ab,kw |
| #6 | [mh "Exercise"] OR "Exercises":ti,ab,kw OR "Exercise, Physical":ti,ab,kw OR "Exercises, Physical":ti,ab,kw OR "Physical Exercise":ti,ab,kw OR "Physical Exercises":ti,ab,kw OR "Exercise, Isometric":ti,ab,kw OR "Exercises, Isometric":ti,ab,kw OR "Isometric Exercises":ti,ab,kw OR "Isometric Exercise":ti,ab,kw OR "Exercise, Aerobic":ti,ab,kw OR "Aerobic Exercise":ti,ab,kw OR "Aerobic Exercises":ti,ab,kw OR "Exercises, Aerobic":ti,ab,kw OR "Exercise Training":ti,ab,kw OR "Exercise Trainings":ti,ab,kw OR "Training, Exercise":ti,ab,kw OR "Trainings, Exercise":ti,ab,kw OR "Physical Activity":ti,ab,kw OR "Activities, Physical":ti,ab,kw OR "Physical Activities":ti,ab,kw OR "Active Breaks":ti,ab,kw OR "Activity Breaks":ti,ab,kw OR "Acute Exercise":ti,ab,kw OR "Acute Exercises":ti,ab,kw OR "Exercise, Acute":ti,ab,kw OR "Exercises, Acute":ti,ab,kw OR [mh "Physical Education and Training"] OR "Physical Education, Training":ti,ab,kw OR "Physical Education":ti,ab,kw OR "Education, Physical":ti,ab,kw OR [mh "Resistance Training"] OR "Training, Resistance":ti,ab,kw OR "Strength Training":ti,ab,kw OR "Training, Strength":ti,ab,kw OR "Weight-Lifting Strengthening Program":ti,ab,kw OR "Strengthening Programs, Weight-Lifting":ti,ab,kw OR "Strengthening Program, Weight-Lifting":ti,ab,kw OR "Weight Lifting Strengthening Program":ti,ab,kw OR "Weight-Lifting Strengthening Programs":ti,ab,kw OR "Weight-Lifting Exercise Program":ti,ab,kw OR "Exercise Programs, Weight-Lifting":ti,ab,kw OR "Exercise Program, Weight-Lifting":ti,ab,kw OR "Weight Lifting Exercise Program":ti,ab,kw OR "Weight-Lifting Exercise Programs":ti,ab,kw OR "Weight-Bearing Strengthening Program":ti,ab,kw OR "Strengthening Programs, Weight-Bearing":ti,ab,kw OR "Strengthening Program, Weight-Bearing":ti,ab,kw OR "Weight Bearing Strengthening Program":ti,ab,kw OR "Weight-Bearing Strengthening Programs":ti,ab,kw OR "Weight-Bearing Exercise Program":ti,ab,kw OR "Exercise Programs, Weight-Bearing":ti,ab,kw OR "Exercise Program, Weight-Bearing":ti,ab,kw OR "Weight Bearing Exercise Program":ti,ab,kw OR "Weight-Bearing Exercise Programs":ti,ab,kw OR [mh "Plyometric Exercise"] OR "Exercise, Plyometric":ti,ab,kw OR "Exercises, Plyometric":ti,ab,kw OR "Plyometric Exercises":ti,ab,kw OR "Plyometric Training":ti,ab,kw OR "Plyometric Trainings":ti,ab,kw OR "Training, Plyometric":ti,ab,kw OR "Trainings, Plyometric":ti,ab,kw OR "Plyometric Drill":ti,ab,kw OR "Drill, Plyometric":ti,ab,kw OR "Drills, Plyometric":ti,ab,kw OR "Plyometric Drills":ti,ab,kw OR "Stretch-Shortening Exercise":ti,ab,kw OR "Exercises, Stretch-Shortening":ti,ab,kw OR "Exercise, Stretch-Shortening":ti,ab,kw OR "Stretch Shortening Exercise":ti,ab,kw OR "Stretch-Shortening Exercises":ti,ab,kw OR "Stretch-Shortening Drill":ti,ab,kw OR "Drills, Stretch-Shortening":ti,ab,kw OR "Drill, Stretch-Shortening":ti,ab,kw OR "Stretch Shortening Drill":ti,ab,kw OR "Stretch-Shortening Drills":ti,ab,kw OR "Stretch-Shortening Cycle Exercise":ti,ab,kw OR "Cycle Exercises, Stretch-Shortening":ti,ab,kw OR "Cycle Exercise, Stretch-Shortening":ti,ab,kw OR "Exercises, Stretch-Shortening Cycle":ti,ab,kw OR "Exercise, Stretch-Shortening Cycle":ti,ab,kw OR "Stretch Shortening Cycle Exercise":ti,ab,kw OR "Stretch-Shortening Cycle Exercises":ti,ab,kw OR [mh "High-Intensity Interval Training"] OR "High Intensity Interval Training":ti,ab,kw OR "High-Intensity Interval Trainings":ti,ab,kw OR "Interval Training, High-Intensity":ti,ab,kw OR "Interval Trainings, High-Intensity":ti,ab,kw OR "Training, High-Intensity Interval":ti,ab,kw OR "Trainings, High-Intensity Interval":ti,ab,kw OR "High-Intensity Intermittent Exercise":ti,ab,kw OR "Exercise, High-Intensity Intermittent":ti,ab,kw OR "Exercises, High-Intensity Intermittent":ti,ab,kw OR "High-Intensity Intermittent Exercises":ti,ab,kw OR "Sprint Interval Training":ti,ab,kw OR "Sprint Interval Trainings":ti,ab,kw OR "small sided game":ti,ab,kw OR "neuromuscular training":ti,ab,kw OR "neuromuscular conditioning":ti,ab,kw OR "complex training":ti,ab,kw OR "contrast training":ti,ab,kw OR "agility":ti,ab,kw OR "sprint":ti,ab,kw |
| #7 | [mh "Athletic Performance"] OR "Athletic Performances":ti,ab,kw OR "Performance, Athletic":ti,ab,kw OR "Performances, Athletic":ti,ab,kw OR "Sports Performance":ti,ab,kw OR "Performance, Sports":ti,ab,kw OR "Performances, Sports":ti,ab,kw OR "Sports Performances":ti,ab,kw OR "jump":ti,ab,kw OR "change of direction":ti,ab,kw OR "speed":ti,ab,kw OR "COD":ti,ab,kw |
| #8 | [pt "Randomized Controlled Trial"] OR [mh "Randomized Controlled Trials as Topic"] OR "Clinical Trials, Randomized":ti,ab,kw OR "Trials, Randomized Clinical":ti,ab,kw OR "Controlled Clinical Trials, Randomized":ti,ab,kw |
| #9 | #1 AND #2 AND #3 AND (#4 OR #5) AND #6 AND #7 AND #8 |

**Table S S1.4** Search strategy of Embase

|  | Searches |
| --- | --- |
| #1 | ('female'/exp OR 'females':ti,ab OR 'women'/exp OR 'girls'/exp OR 'girl'/exp OR 'woman'/exp OR 'women''s groups'/exp OR 'women groups'/exp OR 'women''s group'/exp) |
| #2 | ('adolescent'/exp OR 'adolescents':ti,ab OR 'adolescence':ti,ab OR 'youth':ti,ab OR 'youths':ti,ab OR 'teens':ti,ab OR 'teen':ti,ab OR 'teenagers':ti,ab OR 'teenager':ti,ab OR 'minors'/exp) |
| #3 | ('athletes'/exp OR 'athlete':ti,ab OR 'professional athletes':ti,ab OR 'athlete, professional':ti,ab OR 'athletes, professional':ti,ab OR 'professional athlete':ti,ab OR 'elite athletes':ti,ab OR 'athlete, elite':ti,ab OR 'athletes, elite':ti,ab OR 'elite athlete':ti,ab) |
| #4 | ('team sports'/exp OR 'team sport':ti,ab) |
| #5 | ('basketball'/exp OR 'basketballs':ti,ab OR 'netball':ti,ab OR 'netballs':ti,ab OR 'rugby'/exp OR 'rugby, union play':ti,ab OR 'play rugby, union':ti,ab OR 'union play rugbies':ti,ab OR 'union play rugby':ti,ab OR 'rugby, league play':ti,ab OR 'league play rugby':ti,ab OR 'play rugby, league':ti,ab OR 'football'/exp OR 'american football':ti,ab OR 'football, american':ti,ab OR 'gaelic football'/exp OR 'football, gaelic':ti,ab OR 'ladies gaelic football':ti,ab OR 'football, ladies gaelic':ti,ab OR 'gaelic football, ladies':ti,ab OR 'soccer'/exp OR 'football european':ti,ab OR 'european, football':ti,ab OR 'europeans, football':ti,ab OR 'european football':ti,ab OR 'football, european':ti,ab OR 'volleyball'/exp OR 'volleyballs':ti,ab OR 'cricket sport'/exp OR 'cricket sports':ti,ab OR 'sport, cricket':ti,ab OR 'sports, cricket':ti,ab OR 'handball':ti,ab OR 'hockey'/exp OR 'hockeys':ti,ab OR 'field hockey':ti,ab OR 'field hockeys':ti,ab OR 'hockey, field':ti,ab OR 'hockeys, field':ti,ab OR 'ice hockey':ti,ab OR 'hockey, ice':ti,ab OR 'hockeys, ice':ti,ab OR 'ice hockeys':ti,ab) |
| #6 | ('exercise'/exp OR 'exercises':ti,ab OR 'exercise, physical':ti,ab OR 'exercises, physical':ti,ab OR 'physical exercise':ti,ab OR 'physical exercises':ti,ab OR 'exercise, isometric':ti,ab OR 'exercises, isometric':ti,ab OR 'isometric exercises':ti,ab OR 'isometric exercise':ti,ab OR 'exercise, aerobic':ti,ab OR 'aerobic exercise':ti,ab OR 'aerobic exercises':ti,ab OR 'exercises, aerobic':ti,ab OR 'exercise training':ti,ab OR 'exercise trainings':ti,ab OR 'training, exercise':ti,ab OR 'trainings, exercise':ti,ab OR 'physical activity':ti,ab OR 'activities, physical':ti,ab OR 'physical activities':ti,ab OR 'active breaks':ti,ab OR 'activity breaks':ti,ab OR 'acute exercise':ti,ab OR 'acute exercises':ti,ab OR 'exercise, acute':ti,ab OR 'exercises, acute':ti,ab OR 'physical education and training'/exp OR 'physical education, training':ti,ab OR 'physical education':ti,ab OR 'education, physical':ti,ab OR 'resistance training'/exp OR 'training, resistance':ti,ab OR 'strength training':ti,ab OR 'training, strength':ti,ab OR 'weight-lifting strengthening program':ti,ab OR 'strengthening programs, weight-lifting':ti,ab OR 'strengthening program, weight-lifting':ti,ab OR 'weight lifting strengthening program':ti,ab OR 'weight-lifting strengthening programs':ti,ab OR 'weight-lifting exercise program':ti,ab OR 'exercise programs, weight-lifting':ti,ab OR 'exercise program, weight-lifting':ti,ab OR 'weight lifting exercise program':ti,ab OR 'weight-lifting exercise programs':ti,ab OR 'weight-bearing strengthening program':ti,ab OR 'strengthening programs, weight-bearing':ti,ab OR 'strengthening program, weight-bearing':ti,ab OR 'weight bearing strengthening program':ti,ab OR 'weight-bearing strengthening programs':ti,ab OR 'weight-bearing exercise program':ti,ab OR 'exercise programs, weight-bearing':ti,ab OR 'exercise program, weight-bearing':ti,ab OR 'weight bearing exercise program':ti,ab OR 'weight-bearing exercise programs':ti,ab OR 'plyometric exercise'/exp OR 'exercise, plyometric':ti,ab OR 'exercises, plyometric':ti,ab OR 'plyometric exercises':ti,ab OR 'plyometric training':ti,ab OR 'plyometric trainings':ti,ab OR 'training, plyometric':ti,ab OR 'trainings, plyometric':ti,ab OR 'plyometric drill':ti,ab OR 'drill, plyometric':ti,ab OR 'drills, plyometric':ti,ab OR 'plyometric drills':ti,ab OR 'stretch-shortening exercise':ti,ab OR 'exercises, stretch-shortening':ti,ab OR 'exercise, stretch-shortening':ti,ab OR 'stretch shortening exercise':ti,ab OR 'stretch-shortening exercises':ti,ab OR 'stretch-shortening drill':ti,ab OR 'drills, stretch-shortening':ti,ab OR 'drill, stretch-shortening':ti,ab OR 'stretch shortening drill':ti,ab OR 'stretch-shortening drills':ti,ab OR 'stretch-shortening cycle exercise':ti,ab OR 'cycle exercises, stretch-shortening':ti,ab OR 'cycle exercise, stretch-shortening':ti,ab OR 'exercises, stretch-shortening cycle':ti,ab OR 'exercise, stretch-shortening cycle':ti,ab OR 'stretch shortening cycle exercise':ti,ab OR 'stretch-shortening cycle exercises':ti,ab OR 'high-intensity interval training'/exp OR 'high intensity interval training':ti,ab OR 'high-intensity interval trainings':ti,ab OR 'interval training, high-intensity':ti,ab OR 'interval trainings, high-intensity':ti,ab OR 'training, high-intensity interval':ti,ab OR 'trainings, high-intensity interval':ti,ab OR 'high-intensity intermittent exercise':ti,ab OR 'exercise, high-intensity intermittent':ti,ab OR 'exercises, high-intensity intermittent':ti,ab OR 'high-intensity intermittent exercises':ti,ab OR 'sprint interval training':ti,ab OR 'sprint interval trainings':ti,ab OR 'small sided game':ti,ab OR 'neuromuscular training':ti,ab OR 'neuromuscular conditioning':ti,ab OR 'complex training':ti,ab OR 'contrast training':ti,ab OR 'agility':ti,ab OR 'sprint':ti,ab) |
| #7 | ('athletic performance'/exp OR 'athletic performances':ti,ab OR 'performance, athletic':ti,ab OR 'performances, athletic':ti,ab OR 'sports performance':ti,ab OR 'performance, sports':ti,ab OR 'performances, sports':ti,ab OR 'sports performances':ti,ab OR 'jump':ti,ab OR 'change of direction':ti,ab OR 'speed':ti,ab OR 'cod':ti,ab) |
| #8 | ('randomized controlled trial'/exp OR 'randomized controlled trial':it OR 'clinical trials, randomized':ti,ab OR 'trials, randomized clinical':ti,ab OR 'controlled clinical trials, randomized':ti,ab) |
| #9 | #1 AND #2 AND #3 AND (#4 OR #5) AND #6 AND #7 AND #8 |

**Table S S1.5** Search strategy of SPORTDiscus

|  | Searches |
| --- | --- |
| #1 | (DE "Female" OR DE "Women" OR DE "Girls" OR DE "Girl" OR DE "Woman" OR DE "Women's Groups" OR DE "Women Groups" OR DE "Women's Group" OR TI "Females" OR AB "Females") |
| #2 | (DE "Adolescent" OR DE "Minors" OR TI ("Adolescents" OR "Adolescence" OR "Youth" OR "Youths" OR "Teens" OR "Teen" OR "Teenagers" OR "Teenager") OR AB ("Adolescents" OR "Adolescence" OR "Youth" OR "Youths" OR "Teens" OR "Teen" OR "Teenagers" OR "Teenager")) |
| #3 | (DE "Athletes" OR TI ("Athlete" OR "Professional Athletes" OR "Athlete, Professional" OR "Athletes, Professional" OR "Professional Athlete" OR "Elite Athletes" OR "Athlete, Elite" OR "Athletes, Elite" OR "Elite Athlete") OR AB ("Athlete" OR "Professional Athletes" OR "Athlete, Professional" OR "Athletes, Professional" OR "Professional Athlete" OR "Elite Athletes" OR "Athlete, Elite" OR "Athletes, Elite" OR "Elite Athlete")) |
| #4 | (DE "Team Sports" OR TI "Team Sport" OR AB "Team Sport") |
| #5 | (DE "Basketball" OR DE "Rugby" OR DE "Football" OR DE "Gaelic Football" OR DE "Soccer" OR DE "Volleyball" OR DE "Cricket Sport" OR DE "Hockey" OR TI ("Basketballs" OR "Netball" OR "Netballs" OR "Rugby, Union Play" OR "Play Rugby, Union" OR "Union Play Rugbies" OR "Union Play Rugby" OR "Rugby, League Play" OR "League Play Rugby" OR "Play Rugby, League" OR "American Football" OR "Football, American" OR "Football, Gaelic" OR "Ladies Gaelic Football" OR "Football, Ladies Gaelic" OR "Gaelic Football, Ladies" OR "Football European" OR "European, Football" OR "Europeans, Football" OR "European Football" OR "Football, European" OR "Volleyballs" OR "Cricket Sports" OR "Sport, Cricket" OR "Sports, Cricket" OR "handball" OR "Hockeys" OR "Field Hockey" OR "Field Hockeys" OR "Hockey, Field" OR "Hockeys, Field" OR "Ice Hockey" OR "Hockey, Ice" OR "Hockeys, Ice" OR "Ice Hockeys") OR AB ("Basketballs" OR "Netball" OR "Netballs" OR "Rugby, Union Play" OR "Play Rugby, Union" OR "Union Play Rugbies" OR "Union Play Rugby" OR "Rugby, League Play" OR "League Play Rugby" OR "Play Rugby, League" OR "American Football" OR "Football, American" OR "Football, Gaelic" OR "Ladies Gaelic Football" OR "Football, Ladies Gaelic" OR "Gaelic Football, Ladies" OR "Football European" OR "European, Football" OR "Europeans, Football" OR "European Football" OR "Football, European" OR "Volleyballs" OR "Cricket Sports" OR "Sport, Cricket" OR "Sports, Cricket" OR "handball" OR "Hockeys" OR "Field Hockey" OR "Field Hockeys" OR "Hockey, Field" OR "Hockeys, Field" OR "Ice Hockey" OR "Hockey, Ice" OR "Hockeys, Ice" OR "Ice Hockeys")) |
| #6 | (DE "Exercise" OR DE "Physical Education and Training" OR DE "Resistance Training" OR DE "Plyometric Exercise" OR DE "High-Intensity Interval Training" OR TI ("Exercises" OR "Exercise, Physical" OR "Exercises, Physical" OR "Physical Exercise" OR "Physical Exercises" OR "Exercise, Isometric" OR "Exercises, Isometric" OR "Isometric Exercises" OR "Isometric Exercise" OR "Exercise, Aerobic" OR "Aerobic Exercise" OR "Aerobic Exercises" OR "Exercises, Aerobic" OR "Exercise Training" OR "Exercise Trainings" OR "Training, Exercise" OR "Trainings, Exercise" OR "Physical Activity" OR "Activities, Physical" OR "Physical Activities" OR "Active Breaks" OR "Activity Breaks" OR "Acute Exercise" OR "Acute Exercises" OR "Exercise, Acute" OR "Exercises, Acute" OR "Physical Education, Training" OR "Physical Education" OR "Education, Physical" OR "Training, Resistance" OR "Strength Training" OR "Training, Strength" OR "Weight-Lifting Strengthening Program" OR "Strengthening Programs, Weight-Lifting" OR "Strengthening Program, Weight-Lifting" OR "Weight Lifting Strengthening Program" OR "Weight-Lifting Strengthening Programs" OR "Weight-Lifting Exercise Program" OR "Exercise Programs, Weight-Lifting" OR "Exercise Program, Weight-Lifting" OR "Weight Lifting Exercise Program" OR "Weight-Lifting Exercise Programs" OR "Weight-Bearing Strengthening Program" OR "Strengthening Programs, Weight-Bearing" OR "Strengthening Program, Weight-Bearing" OR "Weight Bearing Strengthening Program" OR "Weight-Bearing Strengthening Programs" OR "Weight-Bearing Exercise Program" OR "Exercise Programs, Weight-Bearing" OR "Exercise Program, Weight-Bearing" OR "Weight Bearing Exercise Program" OR "Weight-Bearing Exercise Programs" OR "Exercise, Plyometric" OR "Exercises, Plyometric" OR "Plyometric Exercises" OR "Plyometric Training" OR "Plyometric Trainings" OR "Training, Plyometric" OR "Trainings, Plyometric" OR "Plyometric Drill" OR "Drill, Plyometric" OR "Drills, Plyometric" OR "Plyometric Drills" OR "Stretch-Shortening Exercise" OR "Exercises, Stretch-Shortening" OR "Exercise, Stretch-Shortening" OR "Stretch Shortening Exercise" OR "Stretch-Shortening Exercises" OR "Stretch-Shortening Drill" OR "Drills, Stretch-Shortening" OR "Drill, Stretch-Shortening" OR "Stretch Shortening Drill" OR "Stretch-Shortening Drills" OR "Stretch-Shortening Cycle Exercise" OR "Cycle Exercises, Stretch-Shortening" OR "Cycle Exercise, Stretch-Shortening" OR "Exercises, Stretch-Shortening Cycle" OR "Exercise, Stretch-Shortening Cycle" OR "Stretch Shortening Cycle Exercise" OR "Stretch-Shortening Cycle Exercises" OR "High Intensity Interval Training" OR "High-Intensity Interval Trainings" OR "Interval Training, High-Intensity" OR "Interval Trainings, High-Intensity" OR "Training, High-Intensity Interval" OR "Trainings, High-Intensity Interval" OR "High-Intensity Intermittent Exercise" OR "Exercise, High-Intensity Intermittent" OR "Exercises, High-Intensity Intermittent" OR "High-Intensity Intermittent Exercises" OR "Sprint Interval Training" OR "Sprint Interval Trainings" OR "small sided game" OR "neuromuscular training" OR "neuromuscular conditioning" OR "complex training" OR "contrast training" OR "agility" OR "sprint") OR AB ("Exercises" OR "Exercise, Physical" OR "Exercises, Physical" OR "Physical Exercise" OR "Physical Exercises" OR "Exercise, Isometric" OR "Exercises, Isometric" OR "Isometric Exercises" OR "Isometric Exercise" OR "Exercise, Aerobic" OR "Aerobic Exercise" OR "Aerobic Exercises" OR "Exercises, Aerobic" OR "Exercise Training" OR "Exercise Trainings" OR "Training, Exercise" OR "Trainings, Exercise" OR "Physical Activity" OR "Activities, Physical" OR "Physical Activities" OR "Active Breaks" OR "Activity Breaks" OR "Acute Exercise" OR "Acute Exercises" OR "Exercise, Acute" OR "Exercises, Acute" OR "Physical Education, Training" OR "Physical Education" OR "Education, Physical" OR "Training, Resistance" OR "Strength Training" OR "Training, Strength" OR "Weight-Lifting Strengthening Program" OR "Strengthening Programs, Weight-Lifting" OR "Strengthening Program, Weight-Lifting" OR "Weight Lifting Strengthening Program" OR "Weight-Lifting Strengthening Programs" OR "Weight-Lifting Exercise Program" OR "Exercise Programs, Weight-Lifting" OR "Exercise Program, Weight-Lifting" OR "Weight Lifting Exercise Program" OR "Weight-Lifting Exercise Programs" OR "Weight-Bearing Strengthening Program" OR "Strengthening Programs, Weight-Bearing" OR "Strengthening Program, Weight-Bearing" OR "Weight Bearing Strengthening Program" OR "Weight-Bearing Strengthening Programs" OR "Weight-Bearing Exercise Program" OR "Exercise Programs, Weight-Bearing" OR "Exercise Program, Weight-Bearing" OR "Weight Bearing Exercise Program" OR "Weight-Bearing Exercise Programs" OR "Exercise, Plyometric" OR "Exercises, Plyometric" OR "Plyometric Exercises" OR "Plyometric Training" OR "Plyometric Trainings" OR "Training, Plyometric" OR "Trainings, Plyometric" OR "Plyometric Drill" OR "Drill, Plyometric" OR "Drills, Plyometric" OR "Plyometric Drills" OR "Stretch-Shortening Exercise" OR "Exercises, Stretch-Shortening" OR "Exercise, Stretch-Shortening" OR "Stretch Shortening Exercise" OR "Stretch-Shortening Exercises" OR "Stretch-Shortening Drill" OR "Drills, Stretch-Shortening" OR "Drill, Stretch-Shortening" OR "Stretch Shortening Drill" OR "Stretch-Shortening Drills" OR "Stretch-Shortening Cycle Exercise" OR "Cycle Exercises, Stretch-Shortening" OR "Cycle Exercise, Stretch-Shortening" OR "Exercises, Stretch-Shortening Cycle" OR "Exercise, Stretch-Shortening Cycle" OR "Stretch Shortening Cycle Exercise" OR "Stretch-Shortening Cycle Exercises" OR "High Intensity Interval Training" OR "High-Intensity Interval Trainings" OR "Interval Training, High-Intensity" OR "Interval Trainings, High-Intensity" OR "Training, High-Intensity Interval" OR "Trainings, High-Intensity Interval" OR "High-Intensity Intermittent Exercise" OR "Exercise, High-Intensity Intermittent" OR "Exercises, High-Intensity Intermittent" OR "High-Intensity Intermittent Exercises" OR "Sprint Interval Training" OR "Sprint Interval Trainings" OR "small sided game" OR "neuromuscular training" OR "neuromuscular conditioning" OR "complex training" OR "contrast training" OR "agility" OR "sprint")) |
| #7 | (DE "Athletic Performance" OR TI ("Athletic Performances" OR "Performance, Athletic" OR "Performances, Athletic" OR "Sports Performance" OR "Performance, Sports" OR "Performances, Sports" OR "Sports Performances" OR "jump" OR "change of direction" OR "speed" OR "COD") OR AB ("Athletic Performances" OR "Performance, Athletic" OR "Performances, Athletic" OR "Sports Performance" OR "Performance, Sports" OR "Performances, Sports" OR "Sports Performances" OR "jump" OR "change of direction" OR "speed" OR "COD")) |
| #8 | (PT "Randomized Controlled Trial" OR DE "Randomized Controlled Trials as Topic" OR TI ("Clinical Trials, Randomized" OR "Trials, Randomized Clinical" OR "Controlled Clinical Trials, Randomized") OR AB ("Clinical Trials, Randomized" OR "Trials, Randomized Clinical" OR "Controlled Clinical Trials, Randomized")) |
| #9 | S1 AND S2 AND S3 AND (S4 OR S5) AND S6 AND S7 AND S8 |

Chinese Databases (CNKI/ Wanfang /VIP)

(女性OR女子OR女孩OR妇女)AND(青少年OR青年OR少年OR青春期OR未成年)AND(运动员OR职业运动员OR精英运动员OR专业运动员)AND(团队运动OR集体项目OR团队项目OR篮球OR橄榄球OR足球OR排球OR板球OR手球OR曲棍球OR冰球)AND(运动OR锻炼OR体力活动OR体育训练OR阻力训练OR力量训练OR举重OR负重训练OR增强式训练OR超等长训练OR快速伸缩复合训练OR高强度间歇训练ORHIITOR冲刺间歇训练ORSITOR小场地比赛OR神经肌肉训练OR复合训练OR对比训练OR敏捷训练OR冲刺)AND(运动表现OR竞技表现OR体育表现OR跳跃OR变向OR速度ORCOD)AND(随机对照试验OR随机对照研究ORRCTOR随机分组OR随机)

Appendix 2: Characteristics of included studies

**Table S2.1** Definitions and Classification of Exercise-Based Intervention Nodes.

| **Node Name** | **Full Name** | **Description & Content** |
| --- | --- | --- |
| **HIIT** | High-Intensity Interval Training | Alternating short bouts of high-intensity exercise with periods of rest or low-intensity recovery, designed to impose substantial cardiopulmonary and metabolic stress within a relatively short time. |
| **SSG** | Small-Sided Games | A modified form of formal match play involving fewer players, reduced playing area, and adjusted rules to simulate real-game situations in a controlled environment. |
| **NMT** | Neuromuscular Training | A supplementary training model integrating general physical conditioning with targeted interventions for movement-control deficits, improving motor control and dynamic joint stability through resistance exercise, core stability, balance, proprioception, agility, and low-intensity jumping activities. |
| **PT** | Plyometric Training | Explosive bodyweight or light-load exercise using the stretch-shortening cycle to improve speed, strength, and peak power. |
| **CT** | Complex Training | Involves an intervention strategy that alternates heavy traditional resistance training (e.g., heavy back squats) with biomechanically similar explosive/plyometric exercises (e.g., loaded jumps or vertical jumps) within the same training session or set. |
| **ST** | Strength training | Exercise using external resistance to improve muscle function, hypertrophy, and physical performance through manipulation of training variables. |
| **HIIT+SSG** | Combined HIIT and SSG | A mixed intervention protocol that explicitly combines high-intensity interval training and small-sided games within the same training cycle or a single training session. |
| **CON** | Control Group | Consists of participants undergoing only standard/routine sport-specific tactical and technical team practice, or maintaining regular school physical education classes/routine daily activities, without the addition of any specialized physical fitness interventions during the study period. |

Appendix 3: Risk of bias of randomized clinical trials

Table S3.1:Study-level risk of bias assessment of the included randomized controlled trials using the Cochrane RoB 2 tool.

| **Author_Year** | **Randomization process** | **Deviations from intended interventions** | **Missing outcome data** | **Measurement of the outcome** | **Selection of the reported result** | **Over all** |
| --- | --- | --- | --- | --- | --- | --- |
| Jurišić,2021 | Low | Some concerns | Low | Low | Low | Some concerns |
| Zhou,2025 | Low | Low | Low | Low | Low | Low |
| Paula F，2018 | Some concerns | Some concerns | Low | Low | Low | High |
| Wen,2024 | Low | Some concerns | Low | Low | Low | Some concerns |
| Nayıroğlu S,2022 | Low | Some concerns | Low | Low | Low | Some concerns |
| Xu,2024 | Low | Some concerns | Low | Low | Low | Some concerns |
| Pang ,2024 | Low | Some concerns | Low | Low | Low | Some concerns |
| Wang,2023 | Low | Some concerns | Low | Low | Low | Some concerns |
| Ma,2021 | Some concerns | Some concerns | Low | Low | Low | High |
| Deng,2020 | Some concerns | Some concerns | Low | Low | Low | High |
| Helmi Chaabene,2021 | Low | Low | Low | Low | Low | Low |
| Gaamouri,2023 | Low | Some concerns | Low | Low | Low | Some concerns |
| Hammami,2018 | Low | Some concerns | Low | Low | Low | Some concerns |
| Hammami,2022 | Low | Low | Low | Low | Low | Low |
| Hammami,2024 | Low | Some concerns | Low | Low | Low | Some concerns |
| Gaamouri,2024 | Low | Some concerns | Low | Low | Low | Some concerns |
| Meszler ,2019 | Low | Low | Low | Low | Low | Low |
| Pardos,2019 | Low | Some concerns | Low | Low | Low | Some concerns |
| Márk Váczi,2022 | Low | Some concerns | Low | Low | Low | Some concerns |
| Ortega,2020 | Low | Low | Low | Low | Low | Low |
| Hammami,2022 | Low | Some concerns | Low | Low | Low | Some concerns |
| Genc H,2019 | Low | Some concerns | Low | Low | Low | Some concerns |
| Fort,2012 | Low | Some concerns | Low | Low | Low | Some concerns |
| MATHISEN,2014 | Some concerns | Some concerns | Low | Low | Low | High |
| Bouteraa,2018 | Low | Low | Low | Low | Low | Low |
| Noutsos,2024 | Low | Low | Low | Low | Low | Low |
| Gaamouri,2023 | Some concerns | Some concerns | Low | Low | Low | High |
| Falch,2022 | Low | Low | Low | Low | Low | Low |
| Idrizovic,2018 | Low | Some concerns | Low | Low | Low | Some concerns |
| Pereira,2015 | Low | Some concerns | Low | Low | Low | Some concerns |
| Martel,2005 | Low | Some concerns | Low | Low | Low | Some concerns |
| Chaabene，2019 | Low | Some concerns | Some concerns | Low | Low | High |
| Hammami，2020 | Low | Low | Low | Low | Low | Low |
| Ozbar，2014 | Low | Some concerns | Low | Low | Low | Some concerns |
| Rubley，2011 | Low | Some concerns | High | Low | Low | High |
| Mokkedes，2022 | Some concerns | Some concerns | Low | Low | Low | High |
| Haghighi，2024 | Low | Low | Low | Low | Low | Low |
| Daniel，2022 | Low | Some concerns | Low | Low | Low | Some concerns |
| Attene，2015 | Low | Some concerns | Low | Low | Low | Some concerns |
| Paes，2022 | Low | Some concerns | Some concerns | Low | Low | High |
| Hammami，2019 | Low | Some concerns | Low | Low | Low | Some concerns |
| Nebojša，2020 | Low | Some concerns | Low | Low | Low | Some concerns |
| Hou，2022 | Low | Some concerns | Low | Some concerns | Low | High |
| Shui，2018 | Low | Some concerns | Low | Low | Low | Some concerns |
| Hanna，2012 | Low | Some concerns | High | Low | Low | High |
| Hakl，2025 | Low | Low | Low | Low | Low | Low |
| Gavala，2023 | Low | Some concerns | Low | Low | Low | Some concerns |

**Appendix 4: Evaluation of inconsistency and heterogeneity**

Table S4.1**:** Global consistency

| outcome | Chi2 | P | tau2 |
| --- | --- | --- | --- |
| CMJ | 2.46 | 0.9820 | 0.7401 |
| SJ | 1.42 | 0.7001 | 0.0113 |
| SLJ | 1.37 | 0.7133 | 1.1073 |
| Sprint 10m | 1.64 | 0.8025 | 0.0035 |
| Sprint 20m | 7.61 | 0.4732 | 0.0239 |
| Sprint 30m | 4.37 | 0.1122 | 0.1019 |
| ModTtest | 3.63 | 0.0569 | 0.0338 |
| Illinois | 17.44 | 0.2665 | ＜0.04 |
| T test | 0.96 | 0.6201 | 0.0862 |

Table S4.2**:** Subgroup heterogeneity estimates for CMJ

| Outcome | Subgroup variable | Subgroup | τ² |
| --- | --- | --- | --- |
| CMJ | Intervention duration | ≤8 weeks | 0.681 |
| CMJ | Intervention duration | >8 weeks | 1.029 |
| CMJ | Sport type | Vertical-dominant | <0.04 |
| CMJ | Sport type | Multidirectional-dominant | 1.480 |

Table S4.3: Side-splitting of CMJ.

| Side | Direct_Coef | Direct_Std_Err | Indirect_Coef | Indirect_Std_Err | Diff_Coef | Diff_Std_Err | P>\|z\| | tau |
| --- | --- | --- | --- | --- | --- | --- | --- | --- |
| HIIT SSG | 1.56977 | 2.514969 | -0.0074478 | 6.187785 | 1.577217 | 6.679302 | 0.813 | 4.230415 |
| HIIT CON | -1.534651 | 3.132272 | -0.5551024 | 5.034876 | -0.9795482 | 5.931643 | 0.869 | 4.231183 |
| SSG PT | 2.597619 | 5.059155 | 1.221268 | 3.408005 | 1.376351 | 6.096191 | 0.821 | 4.227583 |
| SSG CON | -2.489409 | 3.211554 | -2.848292 | 4.561781 | 0.3588828 | 5.528911 | 0.948 | 4.238631 |
| NMT CT | 1.600682 | 4.784335 | 4.345606 | 2.628561 | -2.744924 | 5.458879 | 0.615 | 4.217377 |
| NMT ST | -2.569928 | 4.279479 | 0.50623 | 2.509716 | -3.076158 | 4.961115 | 0.535 | 4.216858 |
| NMT CON | -1.877051 | 2.168103 | -5.471207 | 3.415673 | 3.594157 | 4.04576 | 0.374 | 4.185473 |
| PT CON * | -4.227528 | 1.248294 | -6.748677 | 10.78915 | 2.521149 | 10.84841 | 0.816 | 4.228908 |
| CT ST | -0.8804825 | 3.260329 | -5.862494 | 2.52278 | 4.982011 | 4.122832 | 0.227 | 4.131413 |
| CT CON | -7.541127 | 1.922671 | -3.513347 | 3.535187 | -4.02778 | 4.024808 | 0.317 | 4.169744 |
| ST CON | -2.359571 | 1.650824 | -3.844601 | 3.508476 | 1.48503 | 3.87752 | 0.702 | 4.232099 |

Table S4.4: Side-splitting of SJ.

| Side | Direct_Coef. | Direct_Std. Err. | Indirect_Coef. | Indirect_Std. Err. | Difference_Coef. | Difference_Std. Err. | P>\|z\| | tau |
| --- | --- | --- | --- | --- | --- | --- | --- | --- |
| HIIT  CON | -2.309999 | 1.655484 | -2.778496 | 27.73851 | 0.4684962 | 27.78787 | 0.987 | 0.0000142 |
| PT  CON | -2.828661 | 0.3894938 | -1.793262 | 89.50142 | -1.035399 | 89.50397 | 0.991 | 9.75E-07 |
| CT  ST | -0.7888567 | 0.8536324 | -1.361984 | 0.5941517 | 0.5731273 | 1.035332 | 0.58 | 0.2507438 |
| CT  CON | -3.2375 | 0.3699738 | -1.732709 | 1.64293 | -1.504791 | 1.666841 | 0.367 | 0.2643471 |
| ST  CON | -2.000103 | 0.3820035 | -1.903715 | 1.755275 | -0.0963887 | 1.796798 | 0.957 | 0.2178358 |

Table S4.5: Side-splitting of SLJ.

| Side | Direct Coef. | Direct Std. Err. | Indirect Coef. | Indirect Std. Err. | Difference Coef. | Difference Std. Err. | P>\|z\| | tau |
| --- | --- | --- | --- | --- | --- | --- | --- | --- |
| HIIT CON | -5.0000000 | 12.7644400 | -11.4957600 | 28.0621700 | 6.4957590 | 30.8288200 | 0.833 | 1.4457200 |
| NMT PT | -5.3795110 | 10.9807000 | 2.3854320 | 16.0282800 | -7.7649420 | 19.4291900 | 0.689 | 1.9227300 |
| NMT CT | 0.0267190 | 13.7454900 | -7.4736540 | 13.8319100 | 7.5003730 | 19.5093600 | 0.701 | 1.9251800 |
| PT ST | 8.2006150 | 12.0741200 | -6.9547160 | 8.4934980 | 15.1553300 | 14.7614400 | 0.305 | 1.2523300 |
| PT CON | -15.2375900 | 5.8294840 | 0.2835238 | 11.0569200 | -15.5211100 | 12.5085400 | 0.215 | 9.9107050 |
| CT ST | 0.0553759 | 8.4641150 | -2.9649640 | 10.7638900 | 3.0203400 | 13.6933300 | 0.825 | 1.0514700 |
| CT CON | -10.9052500 | 8.7760950 | -11.2422600 | 9.8684440 | 0.3370081 | 13.2088800 | 0.980 | 1.0785900 |
| ST CON | -3.5695250 | 8.5156810 | -16.3077100 | 8.5496520 | 12.7381900 | 12.0681000 | 0.291 | 1.2196800 |

Table S4.6: Side-splitting of Sprint 10m.

| Side | Direct Coef. | Std. Err. | Indirect Coef. | Std. Err. | Difference Coef. | Std. Err. | P>\|z\| | tau |
| --- | --- | --- | --- | --- | --- | --- | --- | --- |
| HIIT CON | 0.0699999 | 0.0734465 | 0.0792251 | 25.12996 | -0.0092252 | 25.13007 | 1 | 0.0593083 |
| NMT CT | 7.74E-09 | 0.073376 | -0.0761015 | 0.0493639 | 0.0761015 | 0.0884355 | 0.389 | 0.0603458 |
| NMT CON | 0.0280263 | 0.0392515 | 0.1041279 | 0.079239 | -0.0761016 | 0.0884355 | 0.389 | 0.0603458 |
| PT  ST | -3.14E-09 | 0.0797016 | 0.0329548 | 0.0542971 | -0.0329548 | 0.0964392 | 0.733 | 0.0617736 |
| PT CON | 0.1005827 | 0.0365085 | 0.0676279 | 0.0892665 | 0.0329549 | 0.0964391 | 0.733 | 0.0617736 |
| CT ST | -0.01 | 0.0660413 | 0.0447061 | 0.0554229 | -0.0547061 | 0.0862157 | 0.526 | 0.0613307 |
| CT CON | 0.1095566 | 0.0297016 | 0.0168072 | 0.0701999 | 0.0927494 | 0.0763769 | 0.225 | 0.0579893 |
| ST CON | 0.0629107 | 0.0422744 | 0.1042005 | 0.0730105 | -0.0412898 | 0.0846853 | 0.626 | 0.0615553 |

Table S4.7: Side-splitting of Sprint 20m.

| Side | Direct Coef. | Direct Std. Err. | Indirect Coef. | Indirect Std. Err. | Difference Coef. | Difference Std. Err. | P>\|z\| | tau |
| --- | --- | --- | --- | --- | --- | --- | --- | --- |
| HIIT SSG | -0.01 | 0.1704613 | 0.1196428 | 0.2002569 | -0.1296428 | 0.2629827 | 0.622 | 0.1595266 |
| HIIT PT | 0.1500917 | 0.1722058 | -0.2480986 | 0.1342582 | 0.3981903 | 0.2165175 | 0.066 | 0.1430793 |
| HIIT CON | 0.0380407 | 0.1274911 | 0.2473276 | 0.1943384 | -0.2092869 | 0.2302034 | 0.363 | 0.1561734 |
| SSG PT | -0.3037115 | 0.1743279 | 0.0208433 | 0.1754928 | -0.3245548 | 0.2448057 | 0.185 | 0.1485151 |
| SSG CON | 0.1154041 | 0.1760546 | -0.0060637 | 0.1798828 | 0.1214678 | 0.2466087 | 0.622 | 0.1588189 |
| NMT CT | 3.91E-09 | 0.174387 | -0.281851 | 0.1799444 | 0.281851 | 0.250581 | 0.261 | 0.153105 |
| NMT CON | -0.05 | 0.1648312 | 0.231851 | 0.1887371 | -0.2818509 | 0.2505814 | 0.261 | 0.1531053 |
| PT ST | 0.0481208 | 0.1242374 | 0.0168096 | 0.1284816 | 0.0313112 | 0.1787713 | 0.861 | 0.1609268 |
| PT CON | 0.1989307 | 0.0675358 | 0.2095659 | 0.1559374 | -0.0106352 | 0.1699219 | 0.95 | 0.1611836 |
| CT ST | 0.1194135 | 0.163816 | -0.0104614 | 0.132923 | 0.1298749 | 0.2109974 | 0.538 | 0.1591463 |
| CT CON | 0.2355361 | 0.0741635 | 0.0163066 | 0.1971694 | 0.2192295 | 0.2105042 | 0.298 | 0.1539751 |
| ST CON | 0.2254421 | 0.1126372 | 0.0942858 | 0.1264661 | 0.1311564 | 0.1692429 | 0.438 | 0.1557556 |

Table S4.8: Side-splitting of Sprint 30m.

| Side | Direct Coef. | Direct Std. Err. | Indirect Coef. | Indirect Std. Err. | Difference Coef. | Difference Std. Err. | P>\|z\| | tau |
| --- | --- | --- | --- | --- | --- | --- | --- | --- |
| NMT ST | 0.27 | 0.4727499 | -0.3419401 | 0.3608268 | 0.6119401 | 0.5947171 | 0.303 | 0.3191712 |
| NMT CON | 0.1100001 | 0.3193367 | 0.7219072 | 0.5016583 | -0.6119071 | 0.5946738 | 0.303 | 0.3191708 |
| PT ST | 0.0102868 | 0.2080958 | 0.6513097 | 0.2581728 | -0.6410229 | 0.3316 | 0.053 | 0.2748217 |
| PT CON | 0.9109952 | 0.2016948 | 0.2699652 | 0.263934 | 0.64103 | 0.3315991 | 0.053 | 0.2748214 |
| CT CON | 0.3306855 | 0.1889532 | 0.2438029 | 115.5702 | 0.0868826 | 115.5704 | 0.999 | 0.3213033 |
| ST CON | 0.3000884 | 0.1923849 | 0.6303867 | 0.2871712 | -0.3302983 | 0.3446294 | 0.338 | 0.3150826 |
| CON HIIT+SSG | -0.04 | 0.3240867 | -0.6144986 | 200.3315 | 0.5744986 | 200.3317 | 0.998 | 0.3213034 |

Table S4.9: Side-splitting of Mod T test.

| Side | Direct_Coef | Direct_Std_Err | Indirect_Coef | Indirect_Std_Err | Diff_Coef | Diff_Std_Err | P>\|z\| | tau |
| --- | --- | --- | --- | --- | --- | --- | --- | --- |
| NMT CON | -0.0599999 | 0.2354834 | 0.607205 | 40.81923 | -0.667205 | 40.81991 | 0.987 | 0.1842613 |
| PT CON | 0.9400001 | 0.2743071 | -1.059962 | 199.9221 | 1.999963 | 199.9225 | 0.992 | 0.1842601 |
| CT CON | 0.5808142 | 0.1049187 | -0.7007935 | 100.2227 | 1.281608 | 100.2228 | 0.99 | 0.1842609 |
| ST CON | 0.3800001 | 0.199585 | -0.4999513 | 200.2791 | 0.8799514 | 200.2792 | 0.996 | 0.1842602 |

Table S4.10: Side-splitting of T test.

| Side | Direct_Coef | Direct_Std_Err | Indirect_Coef | Indirect_Std_Err | Diff_Coef | Diff_Std_Err | P>\|z\| | tau |
| --- | --- | --- | --- | --- | --- | --- | --- | --- |
| NMT CT | 0.0999994 | 0.2728914 | -0.1235118 | 0.3572527 | 0.2235113 | 0.4495544 | 0.619 | 7.18E-09 |
| NMT CON | 1.11 | 0.1778383 | 1.333446 | 0.4128716 | -0.2234466 | 0.4495435 | 0.619 | 2.07E-06 |
| PT ST | 0.1900015 | 0.1791336 | 0.4081251 | 0.1799904 | -0.2181236 | 0.2539399 | 0.39 | 2.42E-09 |
| PT CON | 0.6638919 | 0.1685287 | 0.4457738 | 0.1899565 | 0.2181181 | 0.25394 | 0.39 | 5.70E-09 |
| CT ST | 0.96 | 0.3036205 | 0.7364871 | 0.3315346 | 0.2235129 | 0.4495561 | 0.619 | 3.66E-10 |
| ST CON | 0.2599994 | 0.0638508 | 0.3748047 | 0.2152913 | -0.1148052 | 0.2245602 | 0.609 | 6.26E-08 |

Table S4.11: Side-splitting of Illinois.

| Side | Direct Coef. | Direct Std. Err. | Indirect Coef. | Indirect Std. Err. | Difference Coef. | Difference Std. Err. | P>\|z\| | tau |
| --- | --- | --- | --- | --- | --- | --- | --- | --- |
| NMT CON | -0.1100006 | 0.3837143 | 0.8261935 | 35.34268 | -0.9361941 | 35.34477 | 0.979 | 0.2937177 |
| PT CON | 0.836159 | 0.2091438 | -1.056021 | 115.4347 | 1.89218 | 115.435 | 0.987 | 0.2937134 |
| CT ST | 0.9920753 | 0.3777402 | 0.2656741 | 0.3538576 | 0.7264012 | 0.5322978 | 0.172 | 0.2954831 |
| CT CON | 0.9575032 | 0.2018193 | 0.9463705 | 0.9828715 | 0.0111327 | 0.9924527 | 0.991 | 0.3526658 |
| ST CON | 0.4501367 | 0.116652 | -1.400273 | 0.5675237 | 1.850409 | 0.5863345 | 0.002 | 0.1311198 |

**Appendix 5: Network maps and forest plots of secondary outcomes**

**Figure S5.1:**Network map illustrating the effects on SJ performance, alongside a forest plot displaying network effect sizes relative to the control group. The size of each node is proportional to the total number of participants in the trials, while the thickness of the connecting lines corresponds to the number of studies evaluating each specific comparison.


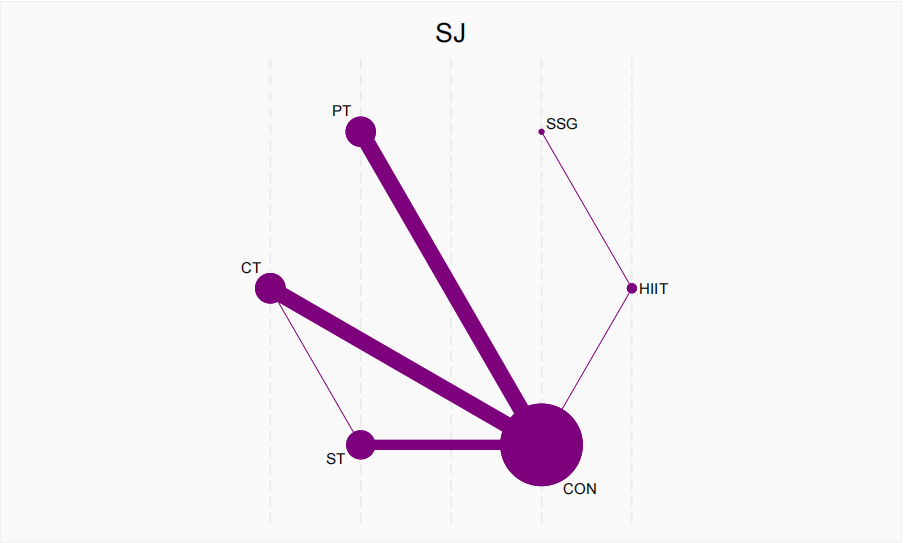


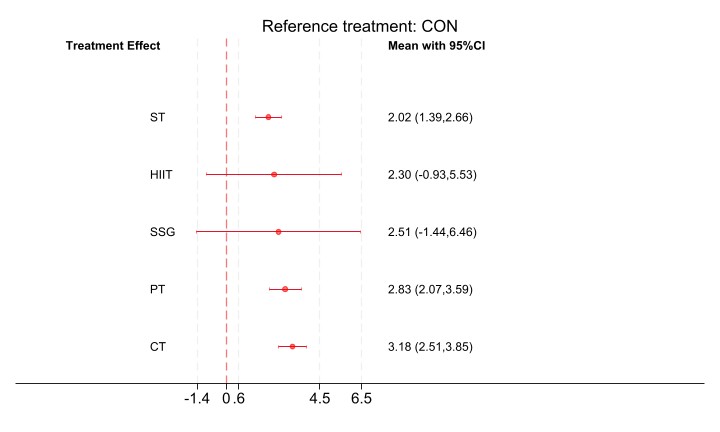


**Figure S5.2:**Network map illustrating the effects on SLJ performance, alongside a forest plot displaying network effect sizes relative to the control group. The size of each node is proportional to the total number of participants in the trials, while the thickness of the connecting lines corresponds to the number of studies evaluating each specific comparison.


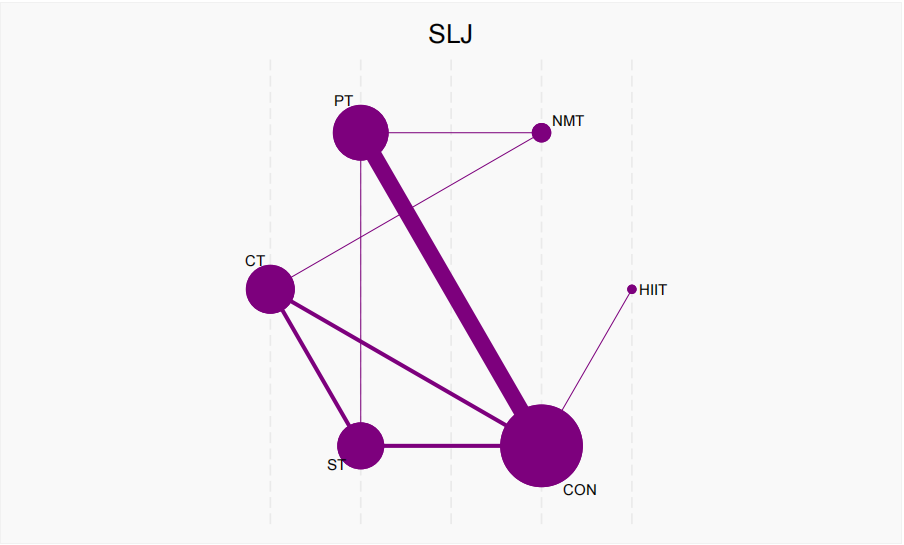

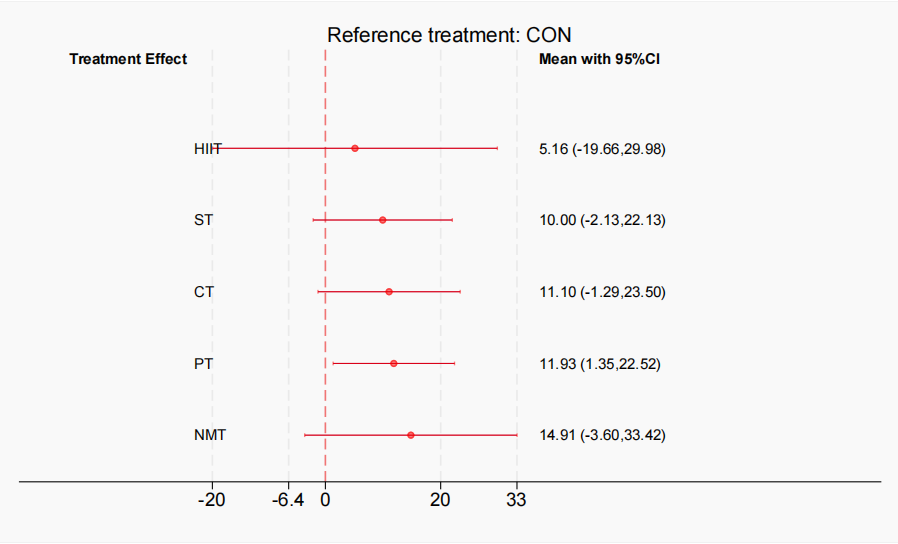


**Figure S5.3:**Network map illustrating the effects on Sprint 10m performance, alongside a forest plot displaying network effect sizes relative to the control group. The size of each node is proportional to the total number of participants in the trials, while the thickness of the connecting lines corresponds to the number of studies evaluating each specific comparison.


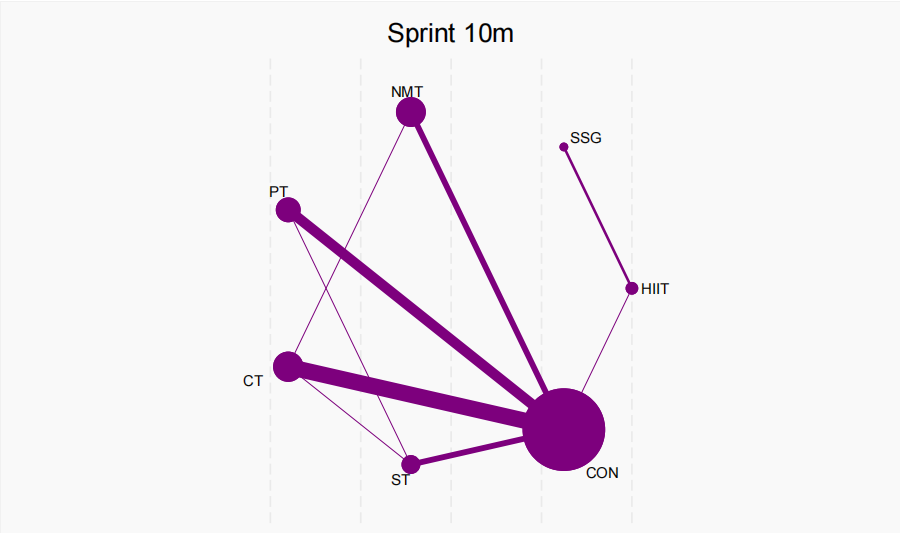


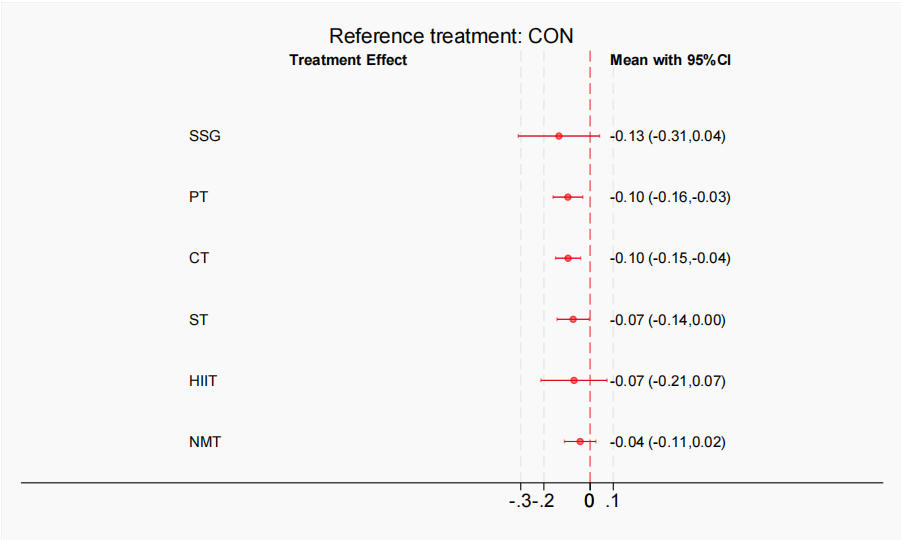


**Figure S5.4:**Network map illustrating the effects on Sprint 30m performance, alongside a forest plot displaying network effect sizes relative to the control group. The size of each node is proportional to the total number of participants in the trials, while the thickness of the connecting lines corresponds to the number of studies evaluating each specific comparison.


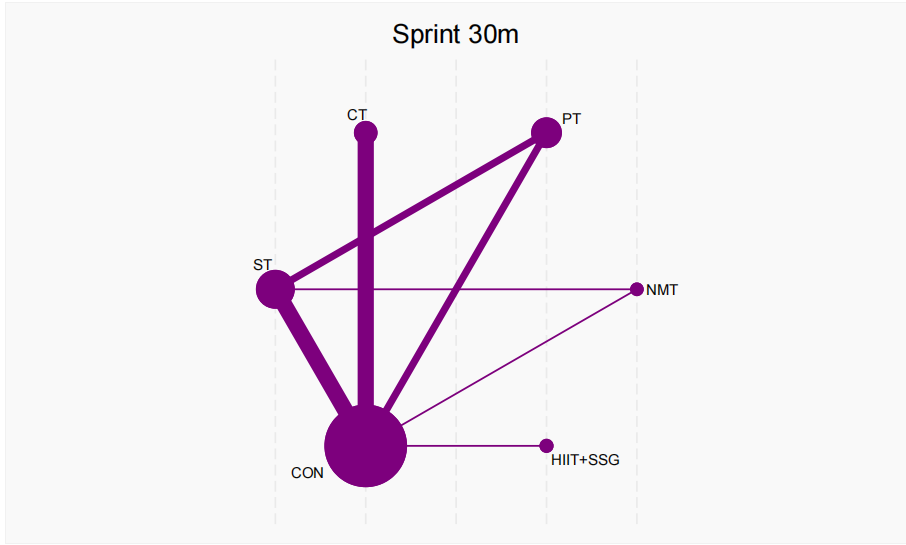

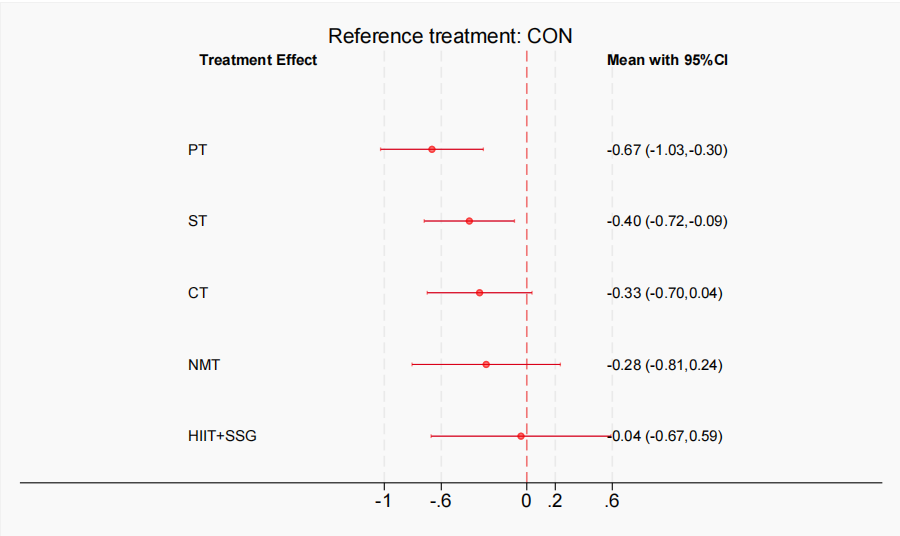


**Figure S5.5:**Network map illustrating the effects on Mod T test performance, alongside a forest plot displaying network effect sizes relative to the control group. The size of each node is proportional to the total number of participants in the trials, while the thickness of the connecting lines corresponds to the number of studies evaluating each specific comparison.


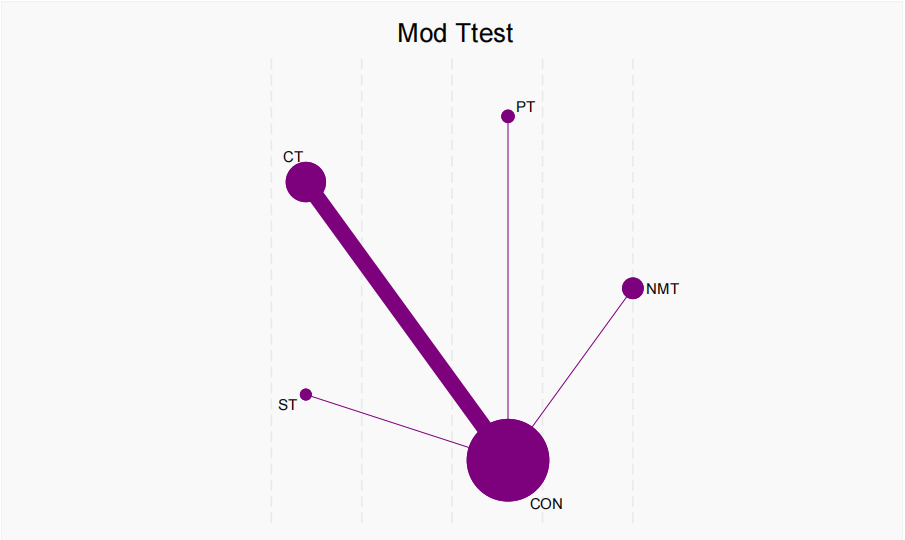

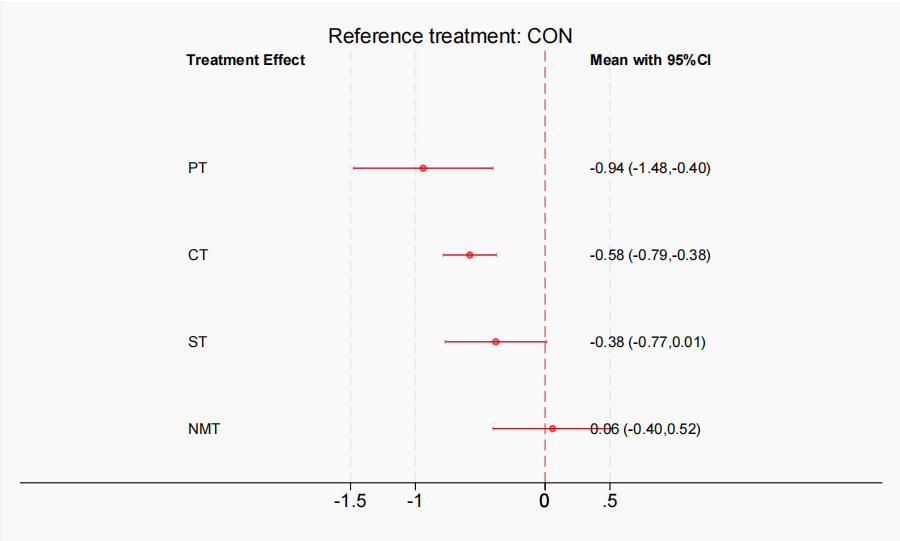


**Figure S5.6:**Network map illustrating the effects on Illinois performance, alongside a forest plot displaying network effect sizes relative to the control group. The size of each node is proportional to the total number of participants in the trials, while the thickness of the connecting lines corresponds to the number of studies evaluating each specific comparison.


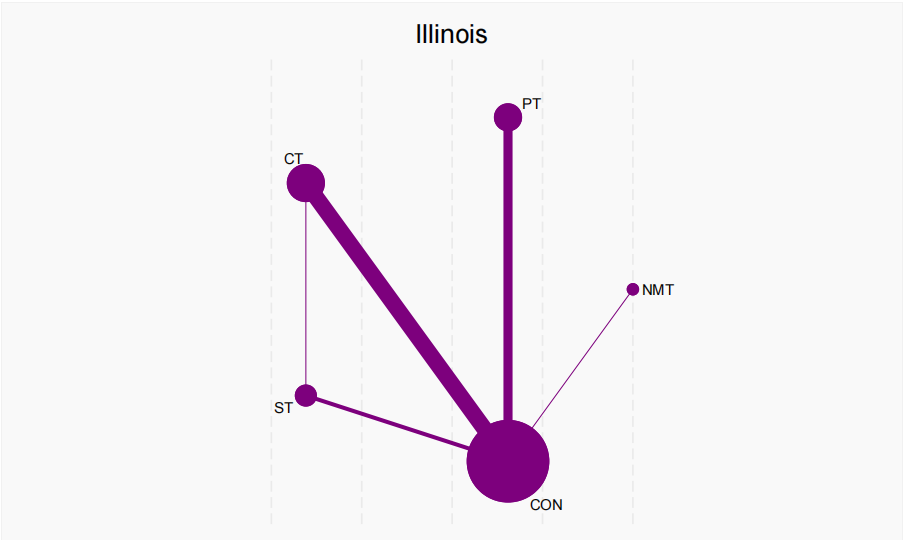

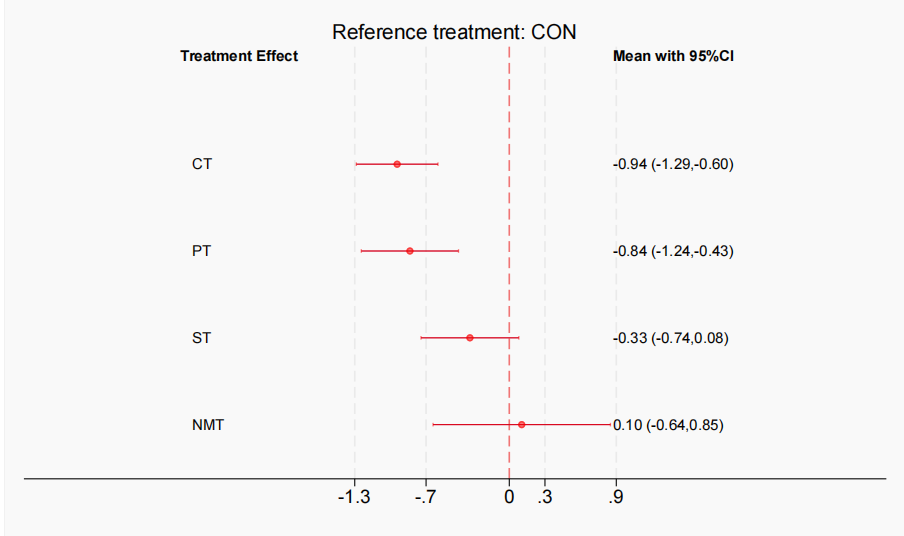


**Appendix 6:** SUCRA and cumulative probability plots

**Figure S6.1** Cumulative ranking curve plots of the different interventions for CMJ performance. A larger surface under the curve (SUCRA) reflects a higher probability of the intervention being the most effective for improving CMJ.

**
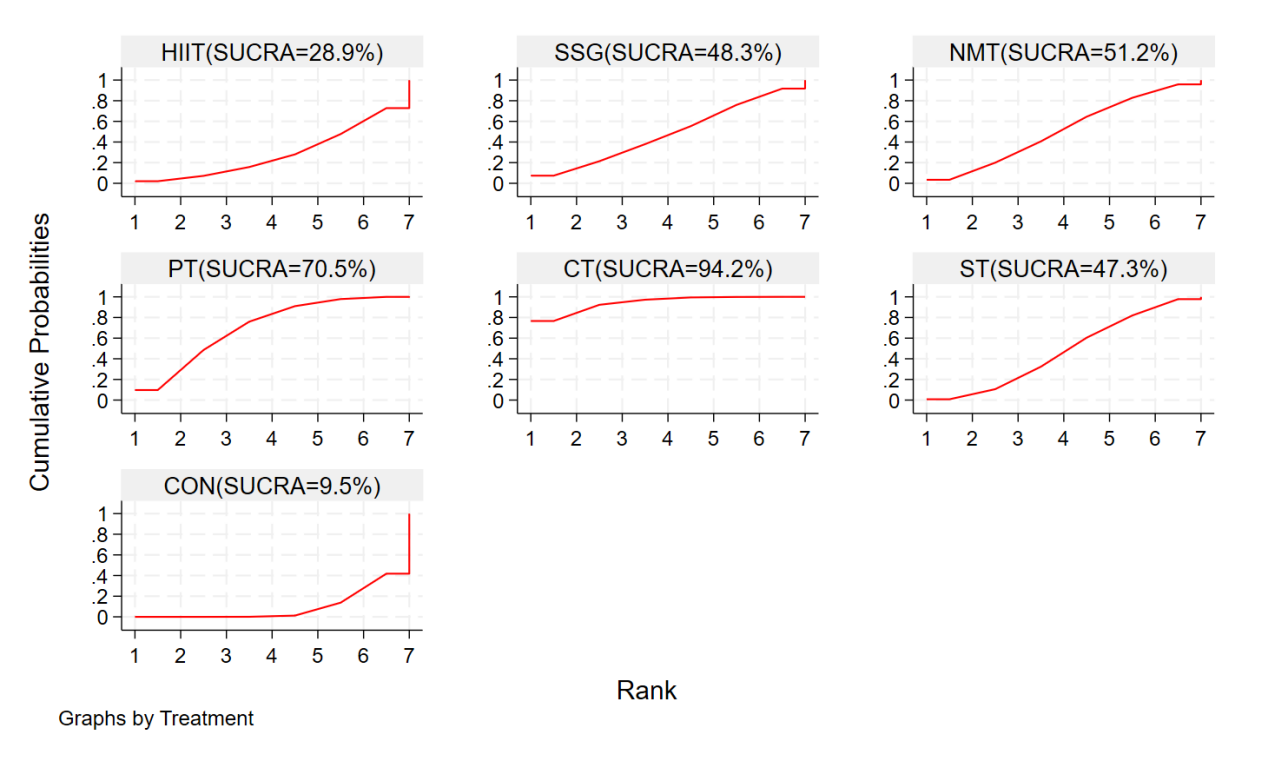
**

**Table S6.1:** SUCRA of the effects of various training interventions on CMJ.

| **Treatm~t** | **SUCRA** | **PrBest** | **MeanRank** |
| --- | --- | --- | --- |
| CT | 94.2 | 76.6 | 1.3 |
| PT | 70.5 | 9.7 | 2.8 |
| NMT | 51.2 | 3.4 | 3.9 |
| SSG | 48.3 | 7.4 | 4.1 |
| ST | 47.3 | 0.8 | 4.2 |
| HIIT | 28.9 | 2.1 | 5.3 |
| CON | 9.5 | 0 | 6.4 |

**Figure S6.2:** Cumulative ranking curve plots of the different interventions for SJ performance. A larger surface under the curve (SUCRA) reflects a higher probability of the intervention being the most effective for improving SJ.


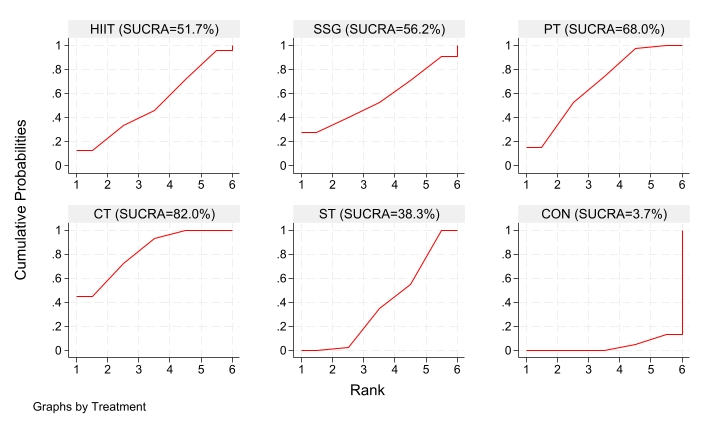


**Table S6.2:** SUCRA of the effects of various training interventions on SJ.

| **Treatm~t** | **SUCRA** | **PrBest** | **MeanRank** |
| --- | --- | --- | --- |
| CT | 82 | 44.9 | 1.9 |
| PT | 68 | 15.3 | 2.6 |
| SSG | 56.2 | 27.6 | 3.2 |
| HIIT | 51.7 | 12.2 | 3.4 |
| ST | 38.3 | 0 | 4.1 |
| CON | 3.7 | 0 | 5.8 |

**Figure S6.3:** Cumulative ranking curve plots of the different interventions for SLJ performance. A larger surface under the curve (SUCRA) reflects a higher probability of the intervention being the most effective for improving SLJ.


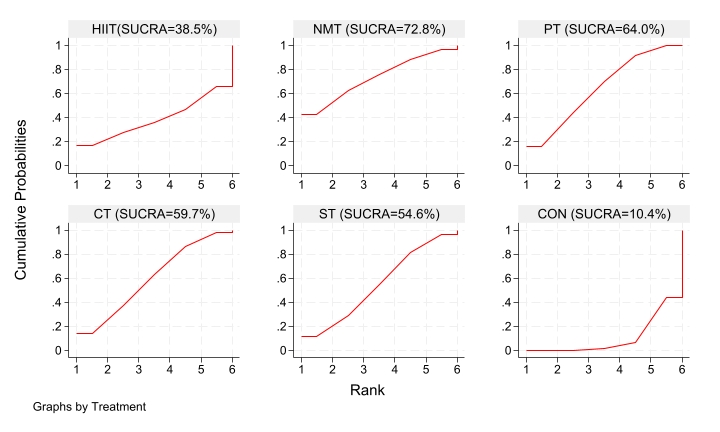


**Table S6.3:** SUCRA of the effects of various training interventions on SLJ.

| **Treatm~t** | **SUCRA** | **PrBest** | **MeanRank** |
| --- | --- | --- | --- |
| NMT | 72.8 | 42.2 | 2.4 |
| PT | 64 | 15.5 | 2.8 |
| CT | 59.7 | 13.9 | 3 |
| ST | 54.6 | 11.5 | 3.3 |
| HIIT | 38.5 | 16.9 | 4.1 |
| CON | 10.4 | 0 | 5.5 |

**Figure S6.4:** Cumulative ranking curve plots of the different interventions for Sprint 10m performance. A larger surface under the curve (SUCRA) reflects a higher probability of the intervention being the most effective for improving Sprint 10m.


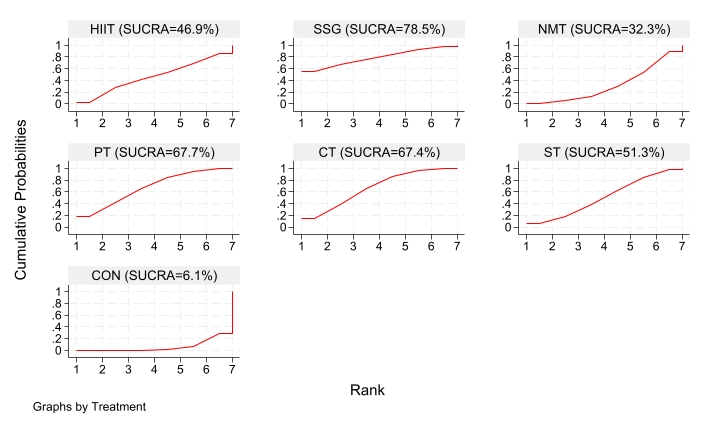


**Table S6.4:** SUCRA of the effects of various training interventions on Sprint 10m.

| **Treatment** | **SUCRA** | **PrBest** | **MeanRank** |
| --- | --- | --- | --- |
| SSG | 78.5 | 55.5 | 2.3 |
| PT | 67.7 | 18.9 | 2.9 |
| CT | 67.4 | 15.3 | 3 |
| ST | 51.3 | 6 | 3.9 |
| HIIT | 46.9 | 3 | 4.2 |
| NMT | 32.3 | 1.3 | 5.1 |
| CON | 6.1 | 0 | 6.6 |

**Figure S6.5:** Cumulative ranking curve plots of the different interventions for Sprint 20m performance. A larger surface under the curve (SUCRA) reflects a higher probability of the intervention being the most effective for improving Sprint 20m.


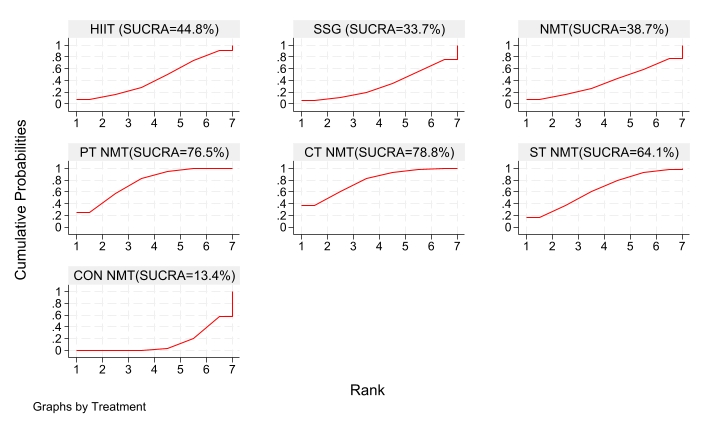


**Table S6.5:** SUCRA of the effects of various training interventions on Sprint 20m.

| Treatm~t | SUCRA | PrBest | MeanRank |
| --- | --- | --- | --- |
| CT | 78.8 | 36.7 | 2.3 |
| PT | 76.5 | 25.5 | 2.4 |
| ST | 64.1 | 16.2 | 3.2 |
| HIIT | 44.8 | 7.7 | 4.3 |
| NMT | 38.7 | 8.4 | 4.7 |
| SSG | 33.7 | 5.5 | 5 |
| CON | 13.4 | 0 | 6.2 |

**Figure S6.6:** Cumulative ranking curve plots of the different interventions for Sprint 30m performance. A larger surface under the curve (SUCRA) reflects a higher probability of the intervention being the most effective for improving Sprint 30m.


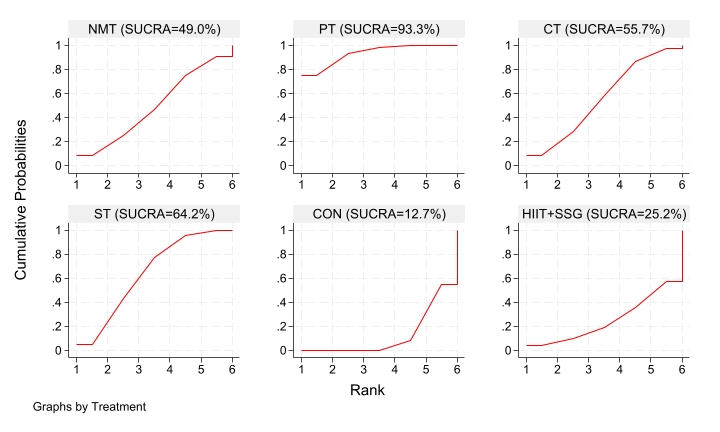


**Table S6.6:** SUCRA of the effects of various training interventions on Sprint 30m.

| **Treatment** | **SUCRA** | **PrBest** | **MeanRank** |
| --- | --- | --- | --- |
| PT | 93.2 | 74.7 | 1.3 |
| ST | 64.2 | 4.9 | 2.8 |
| CT | 55.7 | 8.1 | 3.2 |
| NMT | 49 | 8.6 | 3.5 |
| HIIT+SSG | 25.2 | 3.7 | 4.7 |
| CON | 12.7 | 0 | 5.4 |

**Figure S6.7:** Cumulative ranking curve plots of the different interventions for Mod T test performance. A larger surface under the curve (SUCRA) reflects a higher probability of the intervention being the most effective for improving Mod T test.


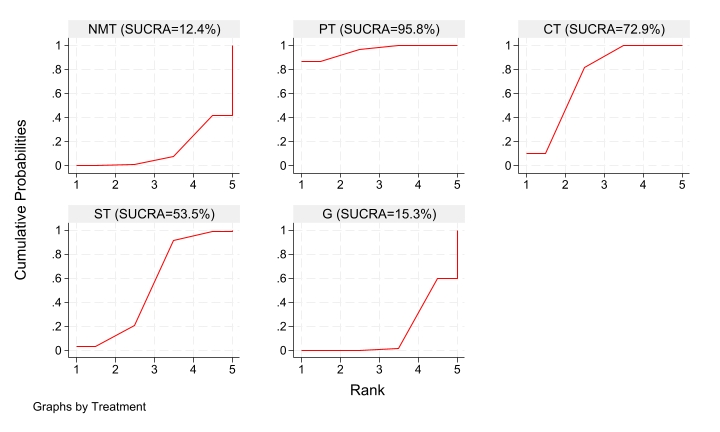


**Table S6.7:** SUCRA of the effects of various training interventions on Mod T test.

| **Treatm~t** | **SUCRA** | **PrBest** | **MeanRank** |
| --- | --- | --- | --- |
| PT | 95.8 | 86.7 | 1.2 |
| CT | 72.9 | 10 | 2.1 |
| ST | 53.5 | 3.2 | 2.9 |
| CON | 15.3 | 0 | 4.4 |
| NMT | 12.4 | 0.1 | 4.5 |

**Figure S6.8:** Cumulative ranking curve plots of the different interventions for T test performance. A larger surface under the curve (SUCRA) reflects a higher probability of the intervention being the most effective for improving T test.


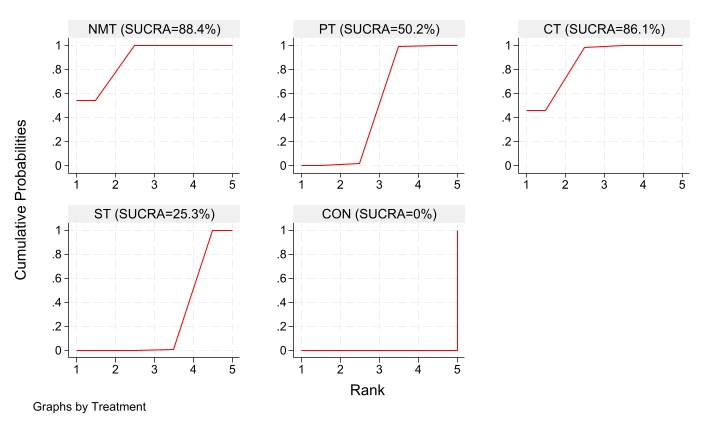


**Table S6.8:** SUCRA of the effects of various training interventions on T test.

| **Treatment** | **SUCRA** | **PrBest** | **MeanRank** |
| --- | --- | --- | --- |
| NMT | 88.4 | 53.8 | 1.5 |
| CT | 86.1 | 46.1 | 1.6 |
| PT | 50.2 | 0.1 | 3 |
| ST | 25.3 | 0 | 4 |
| CON | 0 | 0 | 5 |

**Figure S6.9:** Cumulative ranking curve plots of the different interventions for Illinois performance. A larger surface under the curve (SUCRA) reflects a higher probability of the intervention being the most effective for improving Illinois.

**
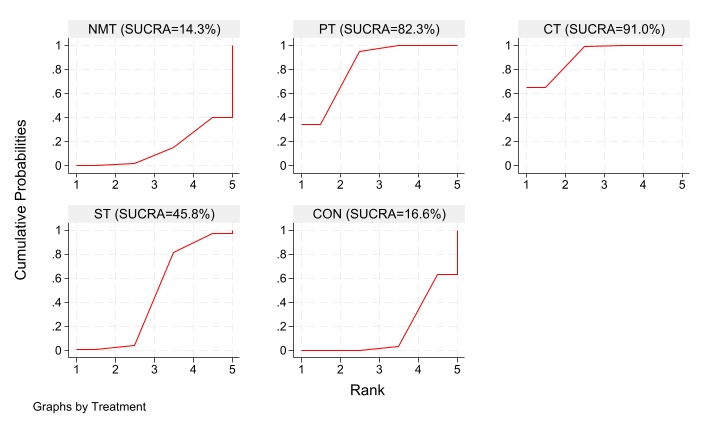
**

**Table S6.9:** SUCRA of the effects of various training interventions on Illinois.

| **Treatment** | **SUCRA** | **PrBest** | **MeanRank** |
| --- | --- | --- | --- |
| CT | 91 | 65 | 1.4 |
| PT | 82.3 | 34.4 | 1.7 |
| ST | 45.8 | 0.3 | 3.2 |
| CON | 16.6 | 0 | 4.3 |
| NMT | 14.3 | 0.3 | 4.4 |

**Appendix 7:** League tables of summary estimates for athletic performance indicators derived from network meta-analysis

**Table S7.1:** CMJ

The columns represent the comparison of the row intervention to the column intervention. The effect estimates are expressed as mean differences and 95% confidence intervals. For example, the mean difference in CMJ for SSG compared to HIIT is -1.36 (95% confidence interval -5.87 to 3.14). A mean difference < 0 favors the intervention in the column, and a mean difference > 0 favors the intervention in the row.

| **HIIT** |  |  |  |  |  |  |
| --- | --- | --- | --- | --- | --- | --- |
| -1.36 (-5.87,3.14) | **SSG** |  |  |  |  |  |
| -1.69 (-7.94,4.57) | -0.32 (-6.56,5.92) | **NMT** |  |  |  |  |
| -3.03 (-8.59,2.53) | -1.67 (-7.15,3.81) | -1.35 (-5.66,2.96) | **PT** |  |  |  |
| -5.40 (-11.50,0.70) | -4.04 (-10.13,2.05) | -3.71 (-8.19,0.76) | -2.37 (-6.46,1.73) | **CT** |  |  |
| -1.40 (-7.29,4.48) | -0.04 (-5.91,5.83) | 0.28 (-3.92,4.48) | 1.63 (-2.13,5.39) | **4.00 (0.05,7.94)** | ST |  |
| 1.23 (-3.91,6.36) | 2.59 (-2.53,7.70) | 2.91 (-0.67,6.49) | **4.26 (1.85,6.66)** | **6.62 (3.31,9.94)** | 2.63 (-0.26,5.52) | CON |

**Table S7.2:** SJ

The columns represent the comparison of the row intervention to the column intervention. The effect estimates are expressed as mean differences and 95% confidence intervals. For example, the mean difference in SJ for SSG compared to HIIT is -0.21 (95% confidence interval -2.49 to 2.07). A mean difference < 0 favors the intervention in the column, and a mean difference > 0 favors the intervention in the row.

| **HIIT** |  |  |  |  |  |
| --- | --- | --- | --- | --- | --- |
| -0.21 (-2.48,2.06) | **SSG** |  |  |  |  |
| -0.53 (-3.85,2.79) | -0.32 (-4.34,3.71) | **PT** |  |  |  |
| -0.88 (-4.18,2.42) | -0.67 (-4.68,3.34) | -0.35 (-1.37,0.66) | **CT** |  |  |
| 0.28 (-3.01,3.57) | 0.49 (-3.51,4.49) | 0.81 (-0.19,1.80) | **1.16 (0.29,2.03)** | **ST** |  |
| 2.30 (-0.93,5.53) | 2.51 (-1.44,6.46) | **2.83 (2.07,3.59)** | **3.18 (2.51,3.85)** | **2.02 (1.39,2.66)** | CON |

**Table S7.3:** SLJ

The columns represent the comparison of the row intervention to the column intervention. The effect estimates are expressed as mean differences and 95% confidence intervals. For example, the mean difference in SLJ for NMT compared to HIIT is -9.75 (95% confidence interval -40.59 to 21.09). A mean difference < 0 favors the intervention in the column, and a mean difference > 0 favors the intervention in the row.

| **HIIT** |  |  |  |  |  |
| --- | --- | --- | --- | --- | --- |
| -9.75 (-40.59,21.09) | **NMT** |  |  |  |  |
| -6.77 (-33.69,20.14) | 2.97 (-14.24,20.19) | **PT** |  |  |  |
| -5.94 (-33.64,21.75) | 3.80 (-14.81,22.41) | 0.83 (-13.74,15.39) | **CT** |  |  |
| -4.84 (-32.42,22.74) | 4.91 (-14.97,24.79) | 1.93 (-11.98,15.85) | 1.11 (-11.44,13.65) | **ST** |  |
| 5.16 (-19.66,29.98) | 14.91 (-3.60,33.42) | **11.93 (1.35,22.52)** | 11.10 (-1.29,23.50) | 10.00 (-2.13,22.13) | CON |

**Table S7.4:** Sprint 10m

The columns represent the comparison of the row intervention to the column intervention. The effect estimates are expressed as mean differences and 95% confidence intervals. For example, the mean difference in Sprint 10m time for SSG compared to HIIT is 0.07 (95% confidence interval -0.04 to 0.17). A mean difference < 0 favors the intervention in the row, and a mean difference > 0 favors the intervention in the column.

| **HIIT** |  |  |  |  |  |  |
| --- | --- | --- | --- | --- | --- | --- |
| 0.07 (-0.04,0.17) | **SSG** |  |  |  |  |  |
| -0.03 (-0.19,0.13) | -0.09 (-0.28,0.10) | **NMT** |  |  |  |  |
| 0.03 (-0.13,0.18) | -0.04 (-0.23,0.15) | 0.05 (-0.04,0.15) | **PT** |  |  |  |
| 0.03 (-0.13,0.18) | -0.04 (-0.22,0.14) | 0.05 (-0.03,0.13) | -0.00 (-0.08,0.08) | **CT** |  |  |
| 0.00 (-0.16,0.16) | -0.06 (-0.25,0.13) | 0.03 (-0.07,0.13) | -0.02 (-0.11,0.06) | -0.02 (-0.10,0.06) | ST |  |
| -0.07 (-0.21,0.07) | -0.13 (-0.31,0.04) | -0.04 (-0.11,0.02) | **-0.10 (-0.16,-0.03)** | **-0.10 (-0.15,-0.04)** | **-0.07 (-0.14,-0.00)** | CON |

**Table S7.5:** Sprint 20m

The columns represent the comparison of the row intervention to the column intervention. The effect estimates are expressed as mean differences and 95% confidence intervals. For example, the mean difference in Sprint 20m time for SSG compared to HIIT is -0.04 (95% confidence interval -0.29 to 0.20). A mean difference < 0 favors the intervention in the row, and a mean difference > 0 favors the intervention in the column.

| HIIT |  |  |  |  |  |  |
| --- | --- | --- | --- | --- | --- | --- |
| -0.04 (-0.29,0.20) | SSG |  |  |  |  |  |
| -0.03 (-0.35,0.29) | 0.02 (-0.33,0.36) | NMT |  |  |  |  |
| 0.10 (-0.12,0.32) | 0.14 (-0.11,0.40) | 0.13 (-0.14,0.40) | PT |  |  |  |
| 0.11 (-0.14,0.36) | 0.15 (-0.13,0.43) | 0.14 (-0.11,0.38) | 0.01 (-0.17,0.18) | CT |  |  |
| 0.07 (-0.19,0.32) | 0.11 (-0.18,0.40) | 0.10 (-0.19,0.38) | -0.03 (-0.20,0.14) | -0.04 (-0.24,0.16) | ST |  |
| -0.10 (-0.31,0.11) | -0.06 (-0.30,0.19) | -0.07 (-0.32,0.17) | **-0.20 (-0.32,-0.08)** | **-0.21 (-0.34,-0.07)** | **-0.17 (-0.33,0.00)** | CON |

**Table S7.6:** Sprint 30m

The columns represent the comparison of the row intervention to the column intervention. The effect estimates are expressed as mean differences and 95% confidence intervals. For example, the mean difference in Sprint 30m time for PT compared to NMT is 0.38 (95% confidence interval -0.23 to 0.99). A mean difference < 0 favors the intervention in the row, and a mean difference > 0 favors the intervention in the column.

| **NMT** |  |  |  |  |  |
| --- | --- | --- | --- | --- | --- |
| 0.38 (-0.23,0.99) | **PT** |  |  |  |  |
| 0.05 (-0.59,0.68) | -0.33 (-0.85,0.18) | **CT** |  |  |  |
| 0.12 (-0.44,0.68) | -0.26 (-0.63,0.10) | 0.07 (-0.41,0.56) | **ST** |  |  |
| -0.28 (-0.81,0.24) | **-0.67 (-1.03,-0.30)** | -0.33 (-0.70,0.04) | **-0.40 (-0.72,-0.09)** | **CON** |  |
| -0.24 (-1.06,0.57) | -0.62 (-1.35,0.10) | -0.29 (-1.02,0.44) | -0.36 (-1.07,0.34) | 0.04 (-0.59,0.67) | HIIT+SSG |

**Table S7.7:** Mod T test

The columns represent the comparison of the row intervention to the column intervention. The effect estimates are expressed as mean differences and 95% confidence intervals. For example, the mean difference in Mod T test time for PT compared to NMT is 1.00 (95% confidence interval 0.29 to 1.70). A mean difference < 0 favors the intervention in the row, and a mean difference > 0 favors the intervention in the column.

| **NMT** |  |  |  |  |
| --- | --- | --- | --- | --- |
| 1.00 (0.29,1.70) | **PT** |  |  |  |
| 0.64 (0.14,1.14) | -0.36 (-0.93,0.22) | **CT** |  |  |
| 0.44 (-0.17,1.04) | -0.56 (-1.22,0.10) | -0.20 (-0.64,0.24) | **ST** |  |
| 0.06 (-0.40,0.52) | **-0.94 (-1.48,-0.40)** | **-0.58 (-0.79,-0.38)** | -0.38 (-0.77,0.01) | **CON** |

**Table S7.8:** T test

The columns represent the comparison of the row intervention to the column intervention. The effect estimates are expressed as mean differences and 95% confidence intervals. For example, the mean difference in T test time for PT compared to NMT is -0.58 (95% confidence interval -0.98 to -0.17). A mean difference < 0 favors the intervention in the row, and a mean difference > 0 favors the intervention in the column.

| **NMT** |  |  |  |  |
| --- | --- | --- | --- | --- |
| **-0.58 (-0.98,-0.17)** | **PT** |  |  |  |
| -0.02 (-0.44,0.41) | 0.56 (0.06,1.06) | **CT** |  |  |
| **-0.87 (-1.21,-0.54)** | -0.30 (-0.55,-0.05) | **-0.86 (-1.30,-0.42)** | **ST** |  |
| **-1.14 (-1.46,-0.82)** | **-0.57 (-0.82,-0.32)** | **-1.13 (-1.57,-0.69)** | **-0.27 (-0.39,-0.15)** | **CON** |

**Table S7.9** Illinois

The columns represent the comparison of the row intervention to the column intervention. The effect estimates are expressed as mean differences and 95% confidence intervals. For example, the mean difference in Illinois test time for PT compared to NMT is 0.94 (95% confidence interval 0.09 to 1.79). A mean difference < 0 favors the intervention in the row, and a mean difference > 0 favors the intervention in the column.

| **NMT** |  |  |  |  |
| --- | --- | --- | --- | --- |
| 0.94 (0.09,1.79) | **PT** |  |  |  |
| 1.05 (0.23,1.87) | 0.11 (-0.43,0.64) | **CT** |  |  |
| 0.44 (-0.42,1.29) | -0.50 (-1.09,0.08) | **-0.61 (-1.11,-0.11)** | **ST** |  |
| 0.10 (-0.64,0.85) | **-0.84 (-1.24,-0.43)** | **-0.94 (-1.29,-0.60)** | -0.33 (-0.74,0.08) | CON |

**Appendix 8:** CINeMA Assessment

We use the CINeMA framework to evidence certainty, assessing it for each network estimate based on the following criteria:

**A: Within study bias:** Risk of bias was assessed across five domains using the Cochrane RoB 2 tool. We classified the overall risk of bias for each study as low if all domains were at low risk, some concerns if at least one domain raised some concerns but none were at high risk, and high if one or more domains were at high risk. See Appendix 3 for the bias assessment. The risk of bias for a pairwise comparison of each exercise-based intervention is shown in figures S8.1-S8.6.

**Figure S8.1:**Risk of bias contribution by intervention group for Jump Performance (CMJ).


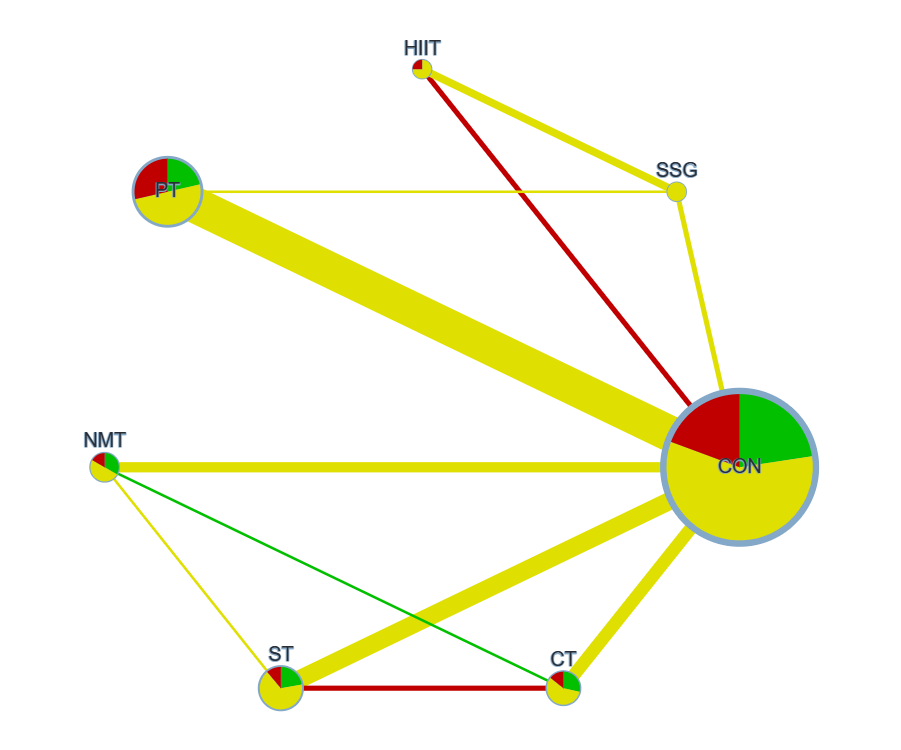


**Figure S8.2:** Overall risk of bias by intervention comparison in Jump Performance (CMJ).


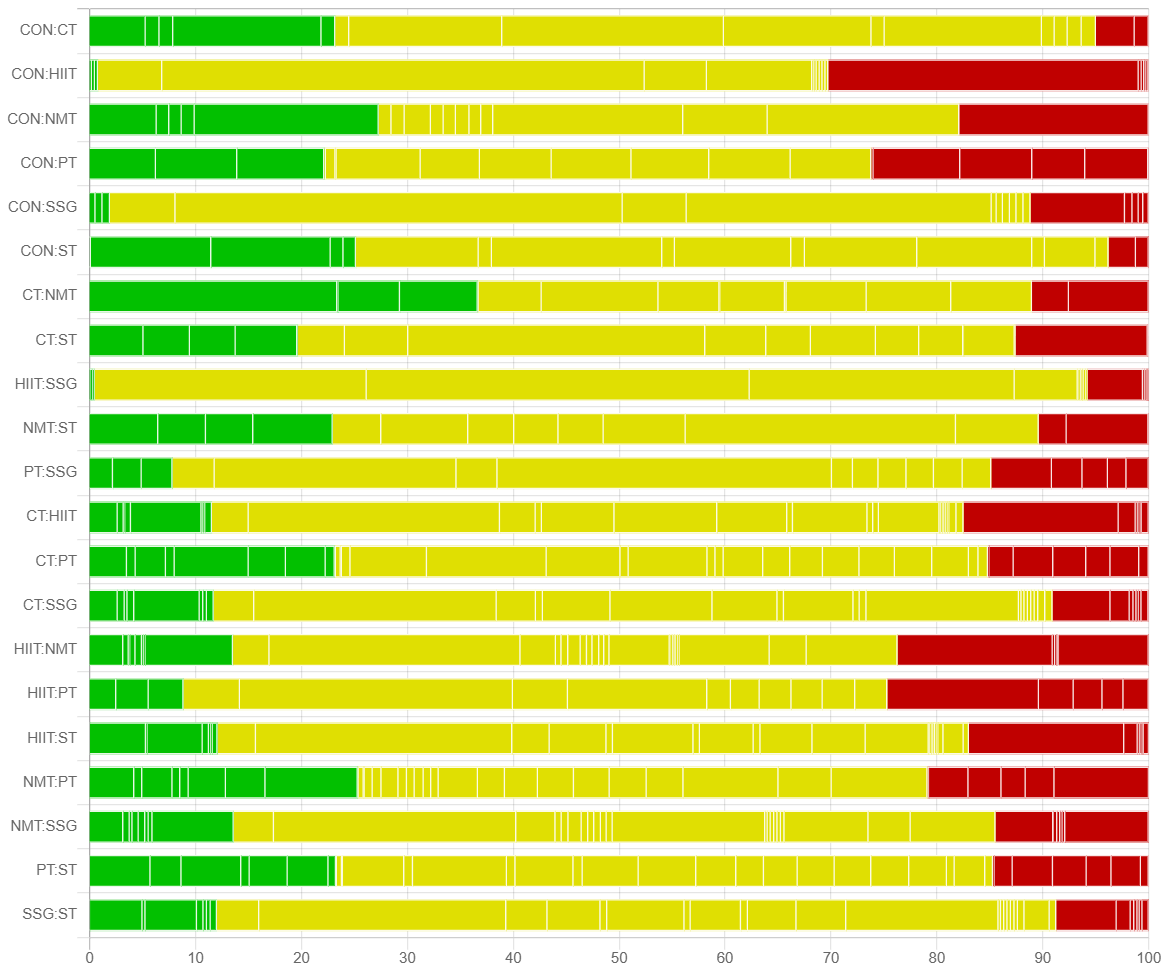


**Figure S8.3:**Risk of bias contribution by intervention group for Linear Sprint Performance (Sprint 20m).


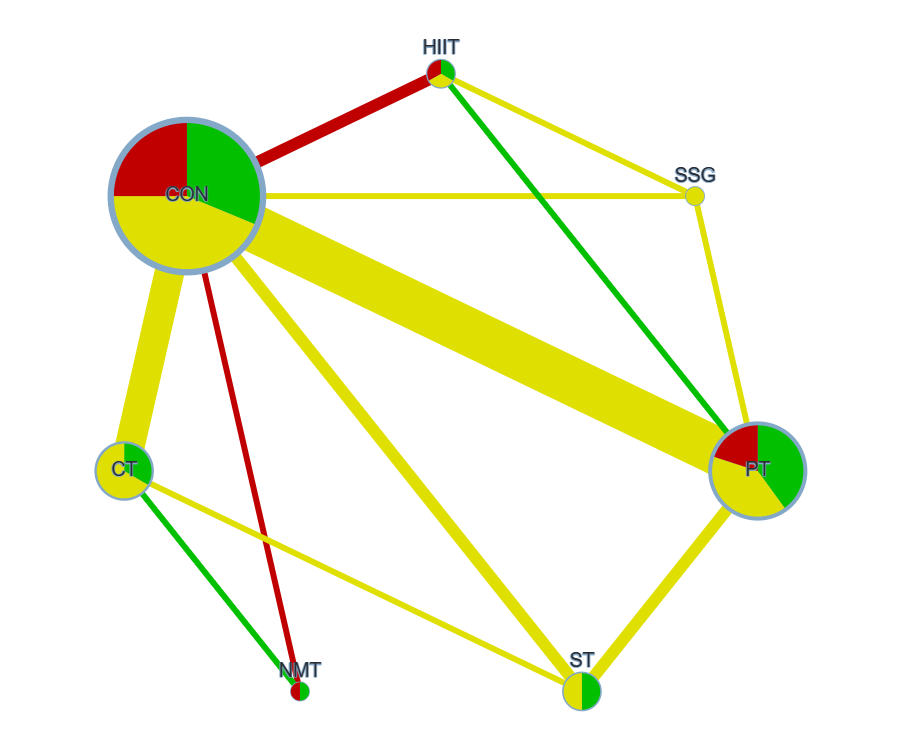


**Figure S8.4:** Overall risk of bias by intervention comparison in Linear Sprint Performance (Sprint 20m).


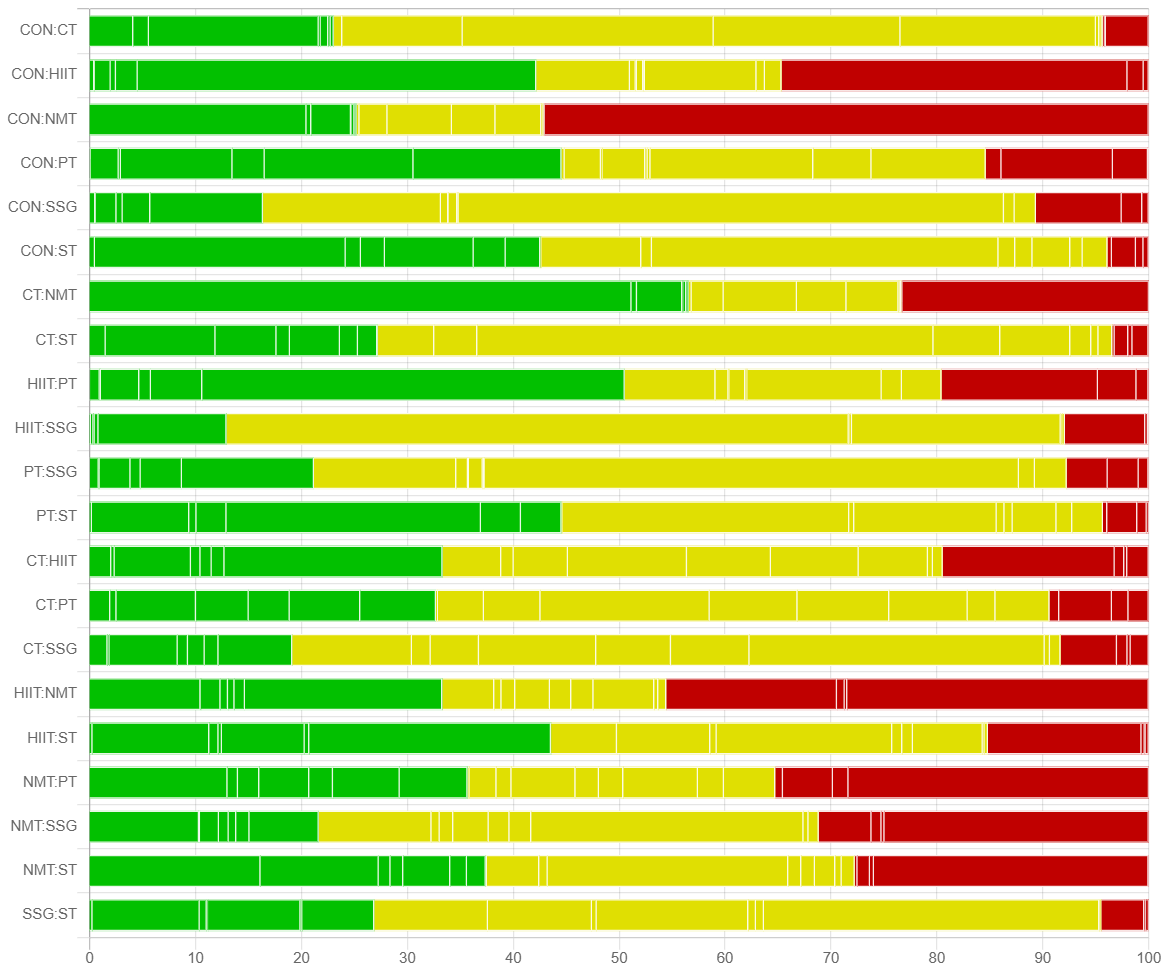


**Figure S8.5:**Risk of bias contribution by intervention group for Change-of-Direction Performance (Standard T-test）


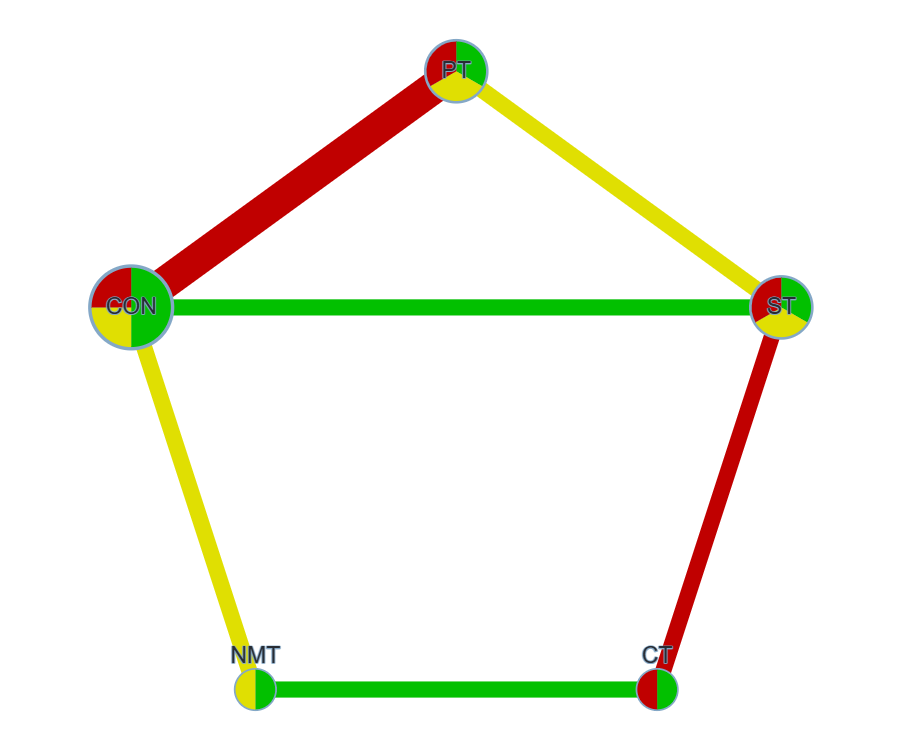


**Figure S8.6:** Overall risk of bias by intervention comparison in Change-of-Direction Performance (Standard T-test).


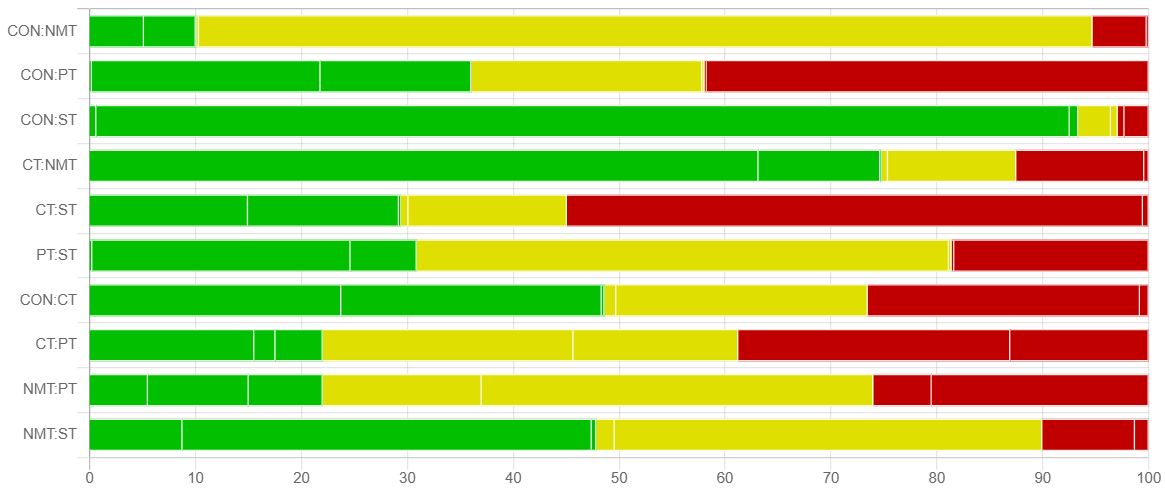


**Table S8.1:** Transitivity (Indirectness) Assessment.

|  | **Baseline variable (Mean ± SD)** | |
| --- | --- | --- |
| **Intervention** | **Age(year)** | **Weight（kg）** |
| HIIT | 16.02 ± 2.03 | 56.62 ± 8.11 |
| SSG | 17.12 ± 1.73 | 56.54 ± 7.60 |
| NMT | 13.74 ± 2.17 | 54.69 ± 9.30 |
| PT | 15.13 ± 1.74 | 57.25 ± 9.62 |
| CT | 16.24 ± 0.61 | 61.58 ± 6.92 |
| ST | 15.35 ± 2.10 | 58.06 ± 11.58 |
| HIIT+SSG | 12.18 ± 1.25 | 56.96 ± 8.28 |
| CON | 14.68 ± 2.09 | 56.05 ± 9.86 |

**Imprecision:** We use the CINeMA website to grade the precision of each comparison.

**Heterogeneity:** We assessed the degree of worry by comparing practical reasoning based on 95% confidence intervals (CIs) while applying the same practical reasoning framework as for imprecision. In particular, we judged the consistency of our findings based on the confidence and prediction intervals associated with practically important effect sizes. And we used the same thresholds of practical significance as described above and followed the recommendations automatically provided by CINeMA (https://cinema.ispm.unibe.ch/).

**Inconsistency:** For inconsistency, we looked at the results for node splitting (Appendix 4) and we saw major problems when p<0.10, but otherwise no problems.

Table S8.2:

CINeMA Results of CMJ.

| **Comparison** | **Within-study bias** | **Reporting bias** | **Indirectness** | **Imprecision** | **Heterogeneity** | **Incoherence** | **Confidence rating** |
| --- | --- | --- | --- | --- | --- | --- | --- |
| CON:CT | Some concerns | Low risk | No concerns | No concerns | Major concerns | No concerns | Moderate |
| CON:HIIT | Some concerns | Low risk | No concerns | Major concerns | No concerns | No concerns | Low |
| CON:NMT | Some concerns | Low risk | No concerns | Major concerns | No concerns | No concerns | Low |
| CON:PT | Some concerns | Low risk | No concerns | No concerns | Major concerns | No concerns | Moderate |
| CON:SSG | Some concerns | Low risk | No concerns | Major concerns | No concerns | No concerns | Low |
| CON:ST | Some concerns | Low risk | No concerns | Major concerns | No concerns | No concerns | Low |
| CT:NMT | Some concerns | Low risk | No concerns | Major concerns | No concerns | No concerns | Low |
| CT:ST | Some concerns | Low risk | No concerns | Major concerns | No concerns | No concerns | Low |
| HIIT:SSG | Some concerns | Low risk | No concerns | Major concerns | No concerns | No concerns | Low |
| NMT:ST | Some concerns | Low risk | No concerns | Major concerns | No concerns | No concerns | Low |
| PT:SSG | Some concerns | Low risk | No concerns | Major concerns | No concerns | No concerns | Low |
| CT:HIIT | Some concerns | Low risk | No concerns | Major concerns | No concerns | Major concerns | Very low |
| CT:PT | Some concerns | Low risk | No concerns | Major concerns | No concerns | Major concerns | Very low |
| CT:SSG | Some concerns | Low risk | No concerns | Major concerns | No concerns | Major concerns | Very low |
| HIIT:NMT | Some concerns | Low risk | No concerns | Major concerns | No concerns | Major concerns | Very low |
| HIIT:PT | Some concerns | Low risk | No concerns | Major concerns | No concerns | Major concerns | Very low |
| HIIT:ST | Some concerns | Low risk | No concerns | Major concerns | No concerns | Major concerns | Very low |
| NMT:PT | Some concerns | Low risk | No concerns | Major concerns | No concerns | Major concerns | Very low |
| NMT:SSG | Some concerns | Low risk | No concerns | Major concerns | No concerns | Major concerns | Very low |
| PT:ST | Some concerns | Low risk | No concerns | Major concerns | No concerns | Major concerns | Very low |
| SSG:ST | Some concerns | Low risk | No concerns | Major concerns | No concerns | Major concerns | Very low |

Table S8.3:

CINeMA Results of Sprint 20m.

| **Comparison** | **Within-study bias** | **Reporting bias** | **Indirectness** | **Imprecision** | **Heterogeneity** | **Incoherence** | **Confidence rating** |
| --- | --- | --- | --- | --- | --- | --- | --- |
| CON:CT | Some concerns | Low risk | No concerns | No concerns | Major concerns | No concerns | Low |
| CON:HIIT | No concerns | Low risk | No concerns | Major concerns | No concerns | No concerns | Low |
| CON:NMT | Major concerns | Low risk | No concerns | Major concerns | No concerns | No concerns | Very low |
| CON:PT | No concerns | Low risk | No concerns | No concerns | Major concerns | No concerns | Moderate |
| CON:SSG | Some concerns | Low risk | No concerns | Major concerns | No concerns | No concerns | Low |
| CON:ST | Some concerns | Low risk | No concerns | No concerns | Major concerns | No concerns | Low |
| CT:NMT | No concerns | Low risk | No concerns | Major concerns | No concerns | No concerns | Low |
| CT:ST | Some concerns | Low risk | No concerns | Major concerns | No concerns | No concerns | Low |
| HIIT:PT | No concerns | Low risk | No concerns | Major concerns | No concerns | No concerns | Low |
| HIIT:SSG | Some concerns | Low risk | No concerns | Major concerns | No concerns | No concerns | Low |
| PT:SSG | Some concerns | Low risk | No concerns | Major concerns | No concerns | No concerns | Low |
| PT:ST | Some concerns | Low risk | No concerns | Major concerns | No concerns | No concerns | Low |
| CT:HIIT | Some concerns | Low risk | No concerns | Major concerns | No concerns | Major concerns | Low |
| CT:PT | Some concerns | Low risk | No concerns | Major concerns | No concerns | Major concerns | Low |
| CT:SSG | Some concerns | Low risk | No concerns | Major concerns | No concerns | Major concerns | Low |
| HIIT:NMT | Major concerns | Low risk | No concerns | Major concerns | No concerns | Major concerns | Very low |
| HIIT:ST | No concerns | Low risk | No concerns | Major concerns | No concerns | Major concerns | Very low |
| NMT:PT | No concerns | Low risk | No concerns | Major concerns | No concerns | Major concerns | Very low |
| NMT:SSG | Some concerns | Low risk | No concerns | Major concerns | No concerns | Major concerns | Very low |
| NMT:ST | No concerns | Low risk | No concerns | Major concerns | No concerns | Major concerns | Very low |
| SSG:ST | Some concerns | Low risk | No concerns | Major concerns | No concerns | Major concerns | Very low |

Table S8.4: CINeMA Results of T-test.

| **Comparison** | **Within-study bias** | **Reporting bias** | **Indirectness** | **Imprecision** | **Heterogeneity** | **Incoherence** | **Confidence rating** |
| --- | --- | --- | --- | --- | --- | --- | --- |
| CON:NMT | Some concerns | Low risk | No concerns | No concerns | No concerns | No concerns | Moderate |
| CON:PT | Major concerns | Low risk | No concerns | No concerns | No concerns | No concerns | Low |
| CON:ST | No concerns | Low risk | No concerns | No concerns | No concerns | No concerns | High |
| CT:NMT | No concerns | Low risk | No concerns | Major concerns | No concerns | No concerns | Low |
| CT:ST | Major concerns | Low risk | No concerns | No concerns | No concerns | No concerns | Low |
| PT:ST | Some concerns | Low risk | No concerns | No concerns | Major concerns | No concerns | Low |
| CON:CT | No concerns | Low risk | No concerns | No concerns | No concerns | No concerns | High |
| CT:PT | Some concerns | Low risk | No concerns | No concerns | Major concerns | No concerns | Low |
| NMT:PT | Some concerns | Low risk | No concerns | No concerns | Major concerns | No concerns | Low |
| NMT:ST | No concerns | Low risk | No concerns | No concerns | No concerns | No concerns | High |

**Appendix 9: Funnel plots**

**Figure S9.1:** Funnel plot of CMJ


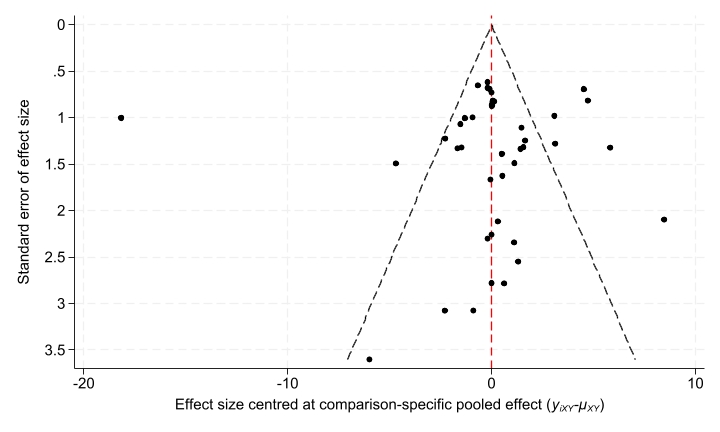


**Figure S9.2:** Funnel plot of SJ


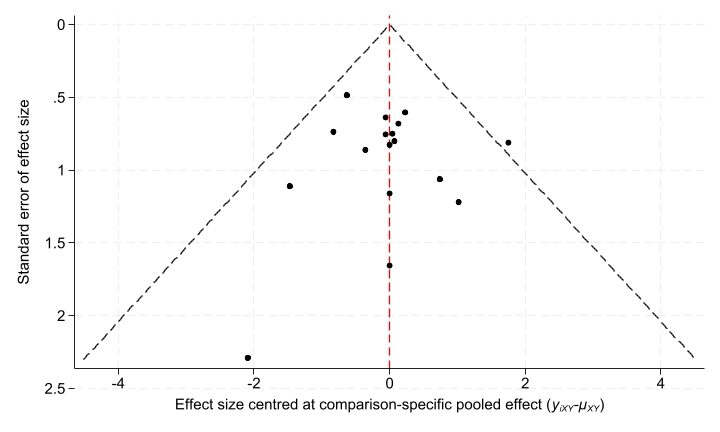


**Figure S9.3:** Funnel plot of SLJ


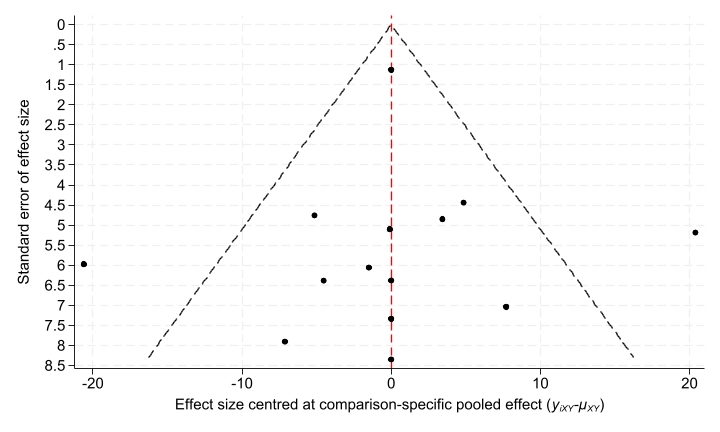


**Figure S9.4:** Funnel plot of Sprint 10m


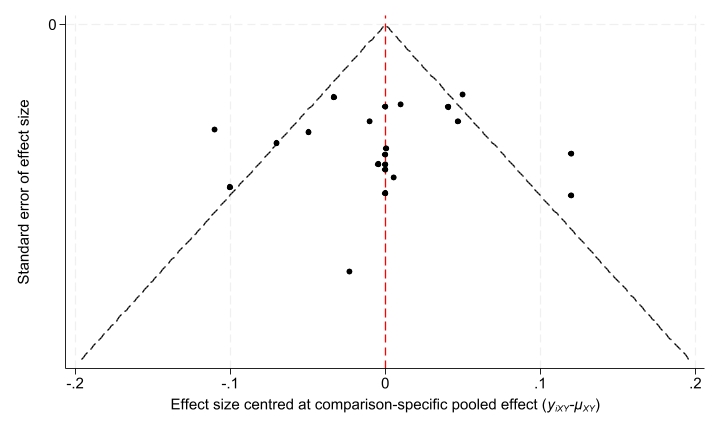


**Figure S9.5:** Funnel plot of Sprint 20m.


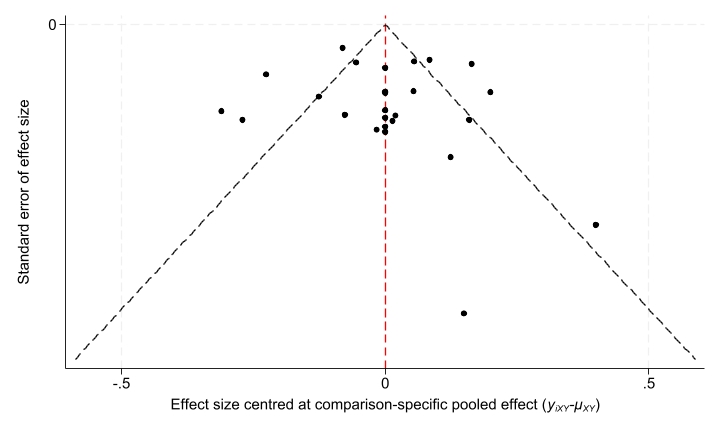


**Figure S9.6:** Funnel plot of Sprint 30m.


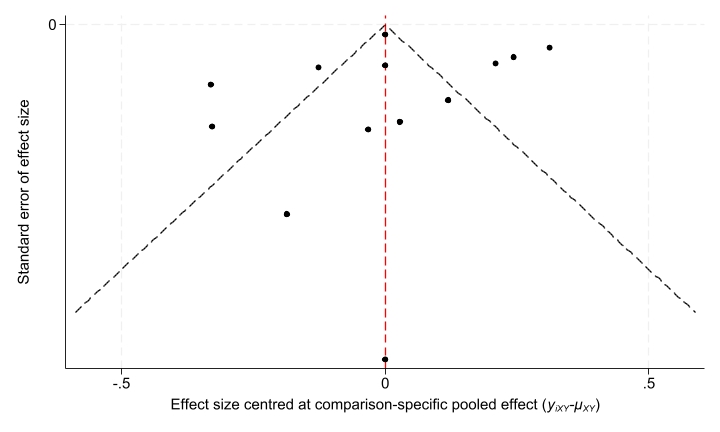


**Figure S9.7:** Funnel plot of Mod T Test.


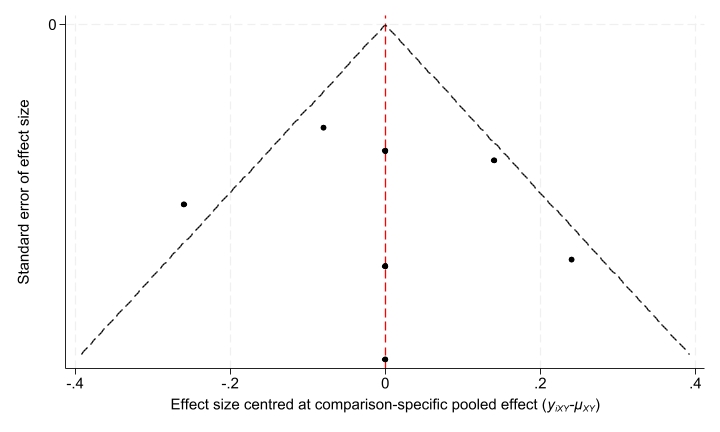


**Figure S9.8:** Funnel plot of T Test.


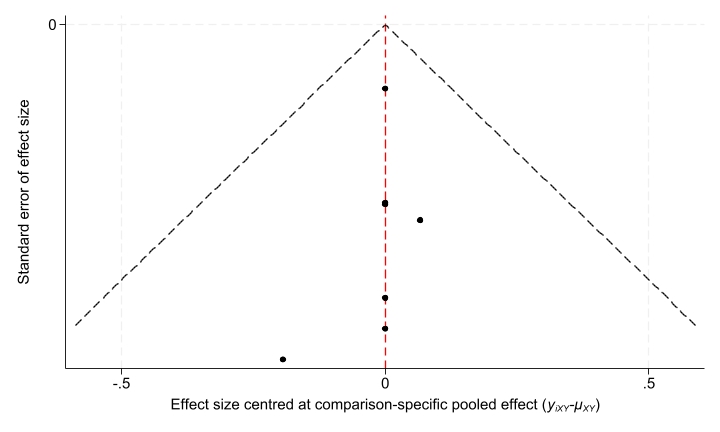


**Figure S9.9:** Funnel plot of Illinois.


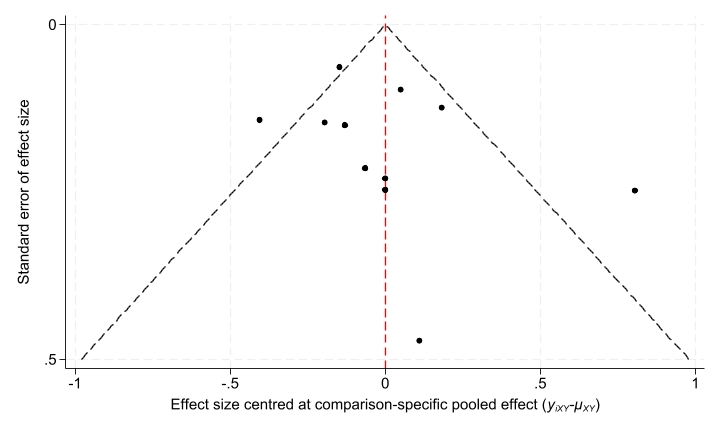


**Appendix 10:** Sensitivity analyses

**Table S10.1:** Sensitivity analyses of primary outcomes

Sensitivity analyses were performed after excluding studies assessed as having a high risk of bias according to the Cochrane RoB 2 tool.

| **Intervention** | **CMJ** | | **Sprint 20m** | | **T Test** | |
| --- | --- | --- | --- | --- | --- | --- |
|  | **Main estimate** | **Sensitivity analyses** | **Main estimate** | **Sensitivity analyses** | **Main estimate** | **Sensitivity analyses** |
| HIIT | 1.23 (-3.91,6.36) | 1.11 (-5.36,7.58) | -0.10 (-0.31,0.11) | -0.14 (-0.39,0.12) | NA | NA |
| SSG | 2.59 (-2.53,7.70) | 2.56 (-3.15,8.28) | -0.06 (-0.30,0.19) | -0.08 (-0.33,0.17) | NA | NA |
| NMT | 2.91 (-0.67,6.49) | 3.43 (-0.59,7.46) | -0.07 (-0.31,0.17) | -0.23 (-0.60,0.13) | -1.14 (-1.46,-0.82) | -1.11 (-1.46,-0.76) |
| PT | 4.26 (1.85,6.66) | 4.69 (1.83,7.55) | -0.20 (-0.31,-0.08) | -0.23 (-0.36,-0.11) | -0.57 (-0.82,-0.32) | -0.57 (-0.82,-0.32) |
| CT | 6.62 (3.31,9.94) | 6.70 (3.31,10.09) | -0.21 (-0.34,-0.07) | -0.23 (-0.37,-0.09) | -1.13 (-1.57,-0.69) | -1.01 (-1.65,-0.37) |
| ST | 2.63 (-0.26,5.52) | 2.70 (-0.26,5.66) | -0.17 (-0.33,0.00) | -0.19 (-0.35,-0.02) | -0.27 (-0.39,-0.15) | -0.27 (-0.39,-0.15) |

**Appendix 11:** Subgroup analyses of the effects of exercise-based interventions on Sports performance

**Table S11.1:** Comparison of the effects of exercise-based interventions on athletic performance with different intervention durations. Studies were stratified into two subgroups according to intervention duration: ≤8 weeks and >8 weeks.

| **Treatment** | **intervention duration** | **CMJ** | **Sprint 20m** | **T test** |
| --- | --- | --- | --- | --- |
| HIIT | ≤8 | 1.51 (-1.72,4.75) | -0.10 (-0.36,0.16) | NA |
|  | ＞8 | NA | NA | NA |
| SSG | ≤8 | 3.04 (-0.51,6.59) | -0.11 (-0.55,0.34) | NA |
|  | ＞8 | 2.14 (-7.72,12.00) | -0.05 (-0.27,0.17) | NA |
| NMT | ≤8 | 2.29 (-0.49,5.07) | -0.06 (-0.51,0.40) | -0.37 (-22.79,22.06) |
|  | ＞8 | 1.71 (-5.72,9.14) | 0.05 (-0.16,0.26) | -1.11 (-1.46,-0.76) |
| PT | ≤8 | 3.53 (1.38,5.68) | -0.11 (-0.36,0.14) | -0.66 (-0.99,-0.33) |
|  | ＞8 | 5.03 (0.88,9.19) | -0.27 (-0.37,-0.17) | -0.45 (-0.82,-0.08) |
| CT | ≤8 | 3.26 (0.18,6.35) | -0.06 (-0.35,0.23) | -0.27 (-22.69,22.16) |
|  | ＞8 | 9.03 (3.69,14.38) | -0.35 (-0.46,-0.23) | -1.22 (-1.83,-0.61) |
| ST | ≤8 | 0.36 (-3.34,4.05) | -0.13 (-0.63,0.36) | NA |
|  | ＞8 | 3.67 (-0.41,7.75) | -0.20 (-0.31,-0.09) | -0.26 (-0.39,-0.13) |

**Table S11.2:** Comparison of the effects of exercise-based interventions on athletic performance across different biomechanical profiles. Included sports were classified into jump- and landing-dominant sports (JLD sports) and multidirectional and change-of-direction-dominant sports (MCD sports) based on their primary movement demands and biomechanical characteristics.

| **Treatment** | **Biomechanical Profile** | **CMJ** | **Sprint 20m** | **T test** |
| --- | --- | --- | --- | --- |
| HIIT | JLD sports | 1.57 (-1.69,4.83) | -0.18 (-0.71,0.35) | NA |
|  | MCD sports | 1.53 (-7.83,10.89) | -0.06 (-0.41,0.28) | NA |
| SSG | JLD sports | 1.55 (-2.87,5.98) | 0.00 (-0.53,0.53) | NA |
|  | MCD sports | 3.09 (-6.37,12.55) | -0.07 (-0.55,0.40) | NA |
| NMT | JLD sports | 2.58 (1.32,3.83) | NA | -1.11 (-1.46,-0.76) |
|  | MCD sports | 3.69 (-2.96,10.35) | -0.07 (-0.32,0.18) | -0.17 (-9.68,9.35) |
| PT | JLD sports | 3.54 (2.00,5.08) | -0.19 (-0.55,0.16) | -0.47 (-1.13,0.18) |
|  | MCD sports | 4.48 (0.55,8.41) | -0.19 (-0.34,-0.04) | -0.73 (-1.11,-0.35) |
| CT | JLD sports | 4.54 (2.19,6.88) | -0.11 (-0.67,0.45) | -1.24 (-2.19,-0.29) |
|  | MCD sports | 7.45 (2.59,12.31) | -0.20 (-0.36,-0.04) | -0.07 (-9.60,9.46) |
| ST | JLD sports | 3.85 (1.43,6.28) | -0.08 (-0.75,0.58) | -0.28 (-1.02,0.46) |
|  | MCD sports | 2.40 (-1.70,6.49) | -0.15 (-0.35,0.05) | -0.26 (-0.39,-0.13) |
